# Apolipoproteins, lipids, lipid-lowering drugs and risk of amyotrophic lateral sclerosis and frontotemporal dementia: a meta-analysis and Mendelian randomisation study

**DOI:** 10.1007/s00415-024-12665-x

**Published:** 2024-09-04

**Authors:** Christos V. Chalitsios, Harriet Ley, Jiali Gao, Martin R. Turner, Alexander G. Thompson

**Affiliations:** Nuffield Department of Clinical Neurosciences, University of Oxford, John Radcliffe Hospital, Level 6, West Wing, Oxford, OX3 9DU UK

**Keywords:** Lipids, Amyotrophic lateral sclerosis, Frontotemporal dementia, ALS, FTD

## Abstract

**Background:**

Amyotrophic lateral sclerosis (ALS) and frontotemporal dementia (FTD) have clinical, pathological and genetic overlapping. Lipid pathways are implicated in ALS. This study examined the effect of blood lipid levels on ALS, FTD risk, and survival in ALS.

**Methods:**

A systematic review and meta-analysis of high and low-density lipoprotein cholesterol (HDL-c and LDL-c), total cholesterol, triglycerides, apolipoproteins B and A1 levels with ALS was performed. Two-sample Mendelian randomisation (MR) analysis sought the causal effects of these exposures on ALS, FTD, and survival in ALS. The effect of lipid-lowering drugs was also examined using genetic proxies for targets of lipid-lowering medications.

**Results:**

Three cohort studies met the inclusion criteria for meta-analysis. Meta-analysis indicated an association between higher LDL-c (HR_per mmol/L_ = 1.07, 95%CI:1.02–1.12; ${I}^{2}$=18%) and lower HDL-c (HR_per mmol/L_ = 0.83, 95%CI:0.74–0.94; ${I}^{2}$=0%) with an increased risk of ALS. MR suggested causal effects of higher LDL-c (OR_IVW_ = 1.085, 95%:CI 1.008–1.168, p_FDR_ = 0.0406), total cholesterol (OR_IVW_ = 1.081, 95%:CI 1.013–1.154, p_FDR_ = 0.0458) and apolipoprotein B (OR_IVW_ = 1.104, 95%:CI 1.041–1.171, p_FDR_ = 0.0061) increasing ALS risk, and higher apolipoprotein B level increasing FTD risk (OR_IVW_ = 1.424, 95%CI 1.072–1.829, p_FDR_ = 0.0382). Reducing LDL-c through *APOB* inhibition was associated with lower ALS (OR_IVW_ = 0.84, 95%CI 0.759–0.929, p_FDR_ = 0.00275) and FTD risk (OR_IVW_ = 0.581, 95%CI 0.387–0.874, p_FDR_ = 0.0362).

**Conclusion:**

These data support the influence of LDL-c and total cholesterol on ALS risk and apolipoprotein B on the risk of ALS and FTD. Potential *APOB* inhibition might decrease the risk of sporadic ALS and FTD. Further work in monogenic forms of ALS and FTD is necessary to determine whether blood lipids influence penetrance and phenotype.

**Supplementary Information:**

The online version contains supplementary material available at 10.1007/s00415-024-12665-x.

## Introduction

Amyotrophic lateral sclerosis (ALS) is a neurodegenerative disease of the corticomotoneuronal system associated with progressive loss of motor neurons, secondary muscle weakness, and death, typically from neuromuscular respiratory failure, within three years of first symptom onset [1]. Frontotemporal dementia (FTD) is characterised by behavioural change or language problems, with a longer disease course when it occurs in isolation [2]. Neither ALS nor FTD has highly effective disease-modifying therapy. ALS and FTD are related in clinical, histopathological and genetic domains. Up to 15% of people with ALS will fulfil the criteria for FTD, but a larger proportion (up to 50%) will have detectable cognitive or behavioural dysfunction on testing [3]. Insoluble neuronal and glial cytoplasmic inclusions of the ubiquitinated protein TDP-43 are the pathological hallmarks of 97% of ALS and 50% of FTD cases [4]. Variants in several genes have been implicated in both ALS and FTD. Of these, an intronic hexanucleotide repeat expansion (HRE) in *C9orf72* is the commonest cause of ALS and FTD, inherited in an autosomal dominant pattern [5]. *C9orf72* HRE can manifest as ALS, FTD or both in the same family [6]. Rare variants in several other genes have been implicated in causing both ALS and FTD [7]. An increasing number of relatives of people affected by monogenetic forms of ALS and FTD are aware of their potentially higher risk of developing ALS or FTD through asymptomatic gene variant carrier status although penetrance is variable and poorly understood. Therefore, the search for modifiable factors influencing the risk of ALS and FTD has become more relevant, with an urgent imperative to provide evidence-based guidance.

Several lifestyle and metabolic factors have been implicated in influencing ALS risk, including body mass index, strenuous exercise and smoking [8, 9]. Large population-based cohort studies indicate that higher levels of low-density lipoprotein cholesterol (LDL-c) and its primary apolipoprotein, apolipoprotein B (ApoB) [10] and lower levels of high-density lipoprotein cholesterol (HDL-c) and its primary apolipoprotein, apolipoprotein A1 (ApoA1) [11] are associated with ALS. Genetic epidemiological techniques which can circumvent the confounding that limits the causal interpretation of observational studies [12], specifically Mendelian randomisation (MR), suggest that high LDL-c and ApoB directly increase the risk of ALS [13]. Observational studies exploring the effect of lipid biomarkers on survival in people with ALS have been somewhat inconsistent, variably indicating potential relationships between lower HDL-c [14], higher total cholesterol (TC), triglyceride (TG), and LDL-c, and improved survival [15–17]. A meta-analysis of observational studies did not support a relationship between biomarkers of lipid metabolism and survival in ALS [18], but a large cohort study demonstrated that increased HDL-c is associated with worse survival [18].

The evidence regarding the impact of lipid-lowering medication use on ALS risk is conflicted, with some studies indicating an increased risk of ALS following statin initiation and others indicating no association [19, 20]. MR methods studying the effect of lipid-lowering drugs on ALS risk, using genetic proxies for the targets of lipid-lowering drugs, suggest a protective effect of HMG-CoA reductase and APOB inhibition. Similar research relating lipid biomarkers and lipid-lowering therapies to the risk of FTD, using either classical epidemiological approaches or genetic epidemiological techniques, is limited.

This study aimed to summarise the current literature surrounding the impact of lipids on ALS and FTD risk using a meta-analysis of observational studies and to study the effect of lipid biomarkers on the risk of ALS and FTD and survival in ALS using MR analysis. Additionally, the potential effect of lipid-lowering drugs on ALS and FTD risk and ALS survival was explored using a genetic proxy approach.

## Methods

### Study design

**Fig. 1:**
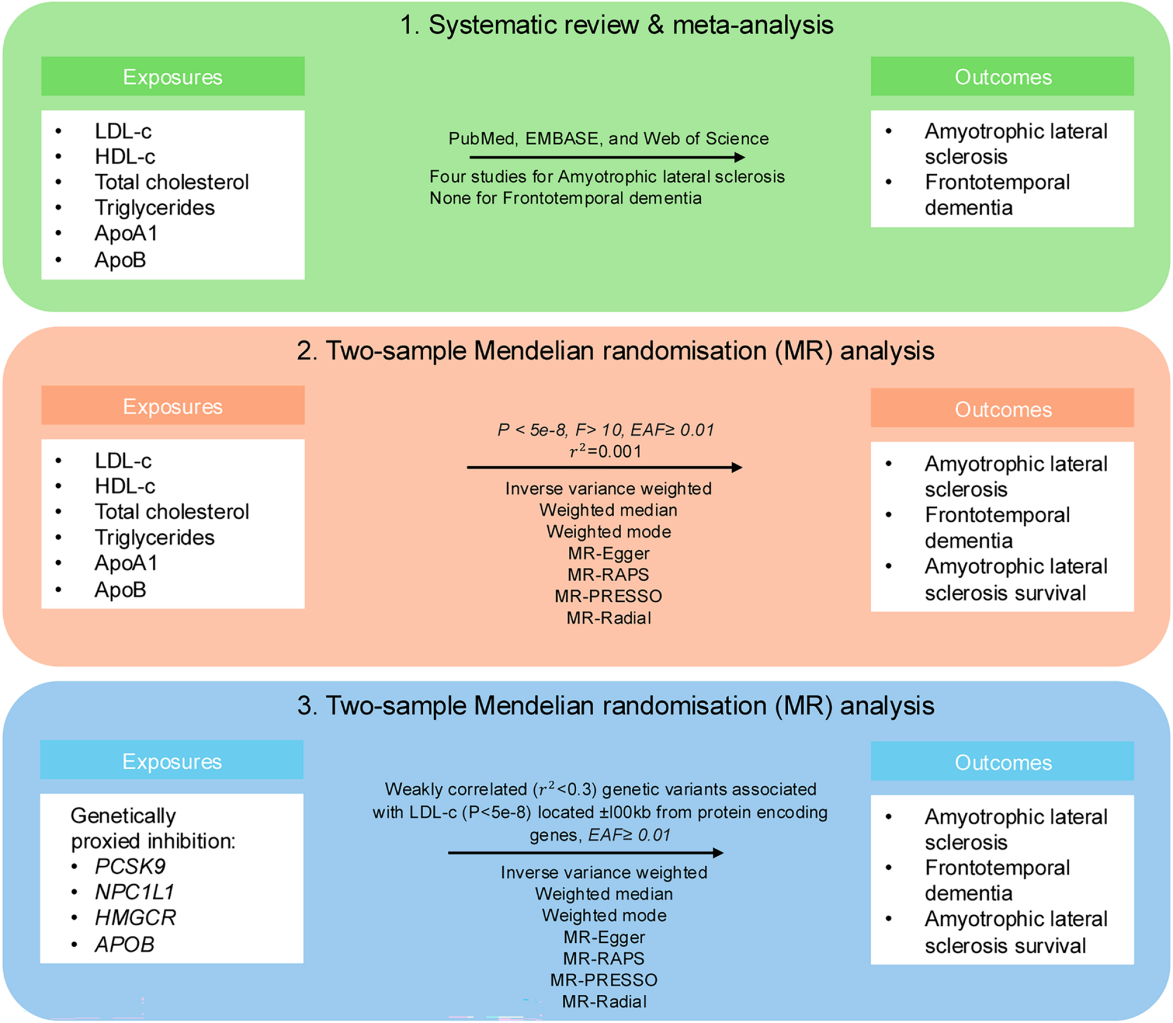
Study overview

First, a systematic review and meta-analysis was conducted to summarise the current literature on the effect of different lipids (HDL-c, LDL-c, TC, and TG) and apolipoproteins (ApoA1, ApoB) on the risk of ALS and FTD. Second, a two-sample MR (Figure S1) was performed to analyse the overall effect of different lipid (HDL-c, LDL-c, TC, and TG) or apolipoprotein (ApoA1, ApoB) traits on the risk of ALS and FTD and survival in ALS. Third, the effect of genetic proxies of lipid-lowering drugs on the outcomes was analysed through drug-target MR. A study design overview is shown in Fig. 1.

### Systematic review

The PRISMA (Preferred Reporting Items for Systematic Reviews and Meta-Analyses) [21] and MOOSE (Meta-analysis of Observational Studies in Epidemiology) guidelines [22] were used. The protocol was registered with the PROSPERO database (CRD42024508035).

#### Eligibility criteria and outcomes

Population-Exposure/Intervention-Comparator-Outcome-Study Design framework was used throughout the review process.

*Participants*: All adults (≥ 18 yrs. old) being at risk of ALS or FTD (i.e. without a pre-existing diagnosis).

*Exposure*: The levels of TC, HDL-c, LDL-c, TG, ApoA1, and ApoB that were obtained before the onset/diagnosis of ALS and FTD.

*Comparator*: The comparator group consisted of people who did not have any diagnosis of ALS or FTD.

*Outcome*: The outcome of interest was a diagnosis of ALS or FTD.

*Design*: All observational studies (cross-sectional, case–control, and cohort) reporting an odds ratio (OR) or hazard ratio (HR) with 95% confidence intervals (CI) of ALS or FTD risk with regard to lipid levels were eligible for inclusion.

Prespecified exclusion criteria were non-human or in vitro studies, non-original research (reviews, editorials, protocols), case reports and case series, letters to editors and foreign language studies.

#### Search and study selection

Three bibliographic databases were searched (PubMed, EMBASE, and Web of Science) from their inception to February 2024 (Table S1). The reference list of included studies and existing systematic reviews was also used to identify additional potentially relevant articles. The results of the searches were imported to Rayyan QCRI [23], and duplicates were removed. Two reviewers (C.V.C and H.L) independently screened the titles and abstracts, and any conflicts were resolved by discussion. Duplicates and records that did not meet eligibility criteria were excluded at this stage. All relevant studies were obtained, and the full text was screened independently by two reviewers (C.V.C, H.L). Any disagreements were resolved through discussion.

Two review authors (C.V.C and H.L) independently extracted data and cross-checked the extracted information. Variables of interest included the author, year of study, study design, country, data source, reference population, type/dose/years of steroid exposure, outcome, demographic of study population, number of people recruited, and adjustment for confounders. When study data were ambiguous or not reported in a form that could be used for formal comparison, the corresponding author of the original publication was contacted via email.

#### Assessment of risk of bias

Two review authors (C.V.C and H.L.) independently assessed the risk of bias for each study. Any disagreements were resolved through discussion. The risk of bias in observational studies was evaluated by incorporating the Newcastle–Ottawa Scale [24]. High quality was defined as a grade of ≥ 7. Both case–control and cohort studies had a maximum score of 9.

### Mendelian randomisation

This study is reported as per the Strengthening the Reporting of Observational Studies in Epidemiology (STROBE) guideline, specific for Mendelian randomisation [25].

#### Genetic variant selection

Independent genetic variants (linkage disequilibrium [LD] clumping threshold of *r*^2^ < 0.001, using a reference panel consisting of individuals of European ancestry from the 1000 Genomes Project Consortium [26], within a 10,000 kb window) associated with LDL-c, HDL-c, TC, TG, ApoA1, and ApoB at genome-wide significance (*p* < 5 × 10^−8^) were identified from a GWAS meta-analysis from the Global Lipids Genetics Consortium (GLGC) using data from up to 146 cohorts and 1,320,016 European participants [27] and from a GWAS that used nuclear magnetic resonance metabolomics to quantify circulating metabolic traits in up to 24,924 European individuals (Table S1) [28]. The F statistics for each variant–trait association was calculated to evaluate instrument strength and potential violation of the first MR assumption (i.e. the IV must be associated with the exposure), and only the genetic variants with an F statistics > 10 were included [12, 29]. Genetic instruments with an effect allele frequency ≥ 0.01 were included.

Lipid-lowering drug classes statins, ezetimibe, PCSK9 inhibitors, and mipomersen were selected based on recent guidelines for managing dyslipidaemia [30, 31] (Table 1). Genes encoding pharmacologic targets of these drugs were identified using the DrugBank database (https://go.drugbank.com). To create genetic instruments, single-nucleotide polymorphisms (SNPs) that demonstrated genome-wide significant association (*p* < 5E-08 $$)$$ with LDL-c levels were first selected [27]. Using previously published methodology for selecting genetic proxies of lipid-lowering drugs [32, 33], these SNPs were further filtered to those within 100 kb of the corresponding gene region (Table 1). Then, they were clumped to an LD threshold of *r*^2^ < 0.3, using a reference panel of individuals of European ancestry from the 1000 Genomes Project Consortium [26]. Four drug-targeting mechanisms were included in the study: HMG-CoA reductase (*HMGCR*) inhibition (statins), Niemann–Pick C1-like protein 1 (*NPC1L1*) inhibition (ezetimibe), *PCSK9* inhibition (Alirocumab and Evolocumab) and Apolipoprotein B-100 (*APOB*) inhibition (Mipomersen).

**Table 1:** Lipid-lowering drug classes, substances, and target genes

Primary pharmacological action	Drug class	Substance	Drug targets	Target genes	Gene region (GRCh37/hg19 by Ensembl)
Reduced LDL-c	HMG-CoA reductase inhibitors	Pravastatin, Simvastatin, Lovastatin, Fluvastatin, Atorvastatin, Rosuvastatin	HMG-CoA reductase	*HMGCR*	chr5:74,632,154–74,657,929
	Cholesterol absorption inhibitors	Ezetimibe	Niemann-Pick C1-like protein 1	*NPC1L1*	chr7:44,552,134–44,580,914
	Proprotein convertase subtilisin/kexin type 9 inhibitors	Alirocumab, Evolocumab	Proprotein convertase subtilisin/kexin type 9	*PCSK9*	chr1:55,505,221–55,530,525
	Antisense oligonucleotide targeting ApoB-100 mRNA	Mipomersen	Apolipoprotein B-100	*APOB*	chr2:21,224,301–21,266,945

*SNPs* single-nucleotide polymorphisms, *chr* chromosome, *mRNA* messenger ribonucleic acid, *LDL*-*c* low-density lipoprotein cholesterol

#### Outcomes

The primary outcomes were ALS, ALS survival, and FTD. For ALS, summary statistics were obtained from the largest available GWAS [34], including 27,205 cases and 110,881 controls of European ancestry from Project MinE. All patients with ALS were diagnosed and ascertained through specialised motor neuron diseases (MND) clinics, where they were diagnosed with ALS according to the (revised) El Escorial Criteria [35] by neurologists specialising in ALS. ALS survival summary statistics were retrieved from a GWAS [36] consisting of 4,256 people with ALS, of whom 3,125 (73.4%) had died with a median survival of 32.8 months. For FTD, summary statistics were obtained from the largest GWAS [37], including 2,154 cases and 4,308 controls of European ancestry. Patients diagnosed according to Neary criteria [38] with behavioural variant FTD, semantic dementia, progressive non-fluent aphasia, and FTD overlapping with motor neuron disease were included in the GWAS.

#### Statistical power

An online tool available at https://shiny.cnsgenomics.com/mRnd/ was employed to perform statistical power calculations [39]. For a type 1 error of 5% and the corresponding variance explained of each exposure trait, the minimum detectable effect size at a range of power thresholds was calculated (Figures S2–S7).

#### Positive control analysis

Positive control analysis was performed with coronary artery disease (CAD) to validate the selection of drug-target genetic variants, given the recognised benefits of lipid-lowering drugs in this context. Summary statistics for CAD were obtained from the coronary artery disease genome-wide Replication and Meta-analysis plus the Coronary Artery Disease Genetics Consortium (CARDIo-GRAMplusC4D) [40].

### Statistical analysis

#### Meta-analysis

Narrative synthesis of evidence was conducted for all included studies. Meta-analyses of each lipid and apolipoprotein were performed based on the DerSimonian–Laird estimator and random effects model to summarise the estimated effect sizes, which were acquired from the included references based on the maximally adjusted models. The results were visually shown in forest plots. The percentage of variability in the effect sizes not caused by sampling error was tested using the Higgins’ ${I}^{2}$ test, and the significance of heterogeneity was examined using the chi-squared statistic. Standardised HR was back-transformed to mmol/l or g/l by dividing by the study standard deviation. For the meta-analysis, we considered an α level of 0.05 as statistically significant. Statistical analysis and meta-analysis were conducted in R version 4.3.1 using the “meta” package.

#### Mendelian randomisation

The primary analysis was random effects inverse variance weighted (IVW) MR [41]. To account for potential horizontal pleiotropy, several MR sensitivity analyses (MR-Egger [42], weighted median [43], and weighted mode [44]) were performed, each providing a valid MR estimate under different combinations of assumptions. To detect potential outlying IVs, we implemented the MR pleiotropy residual sum and outlier test (MR-PRESSO), which identifies and excludes outliers, applying a random effects IVW model [45]. In addition, MR, using a robust-adjusted profile score (MR-RAPS) [46], was used to control for pleiotropy through a random effects model, considering the variance in instrument effect sizes. When there was evidence of heterogeneity (Cochran’s Q statistic *p* value > 0.05), Radial MR analysis was performed [47] in the two-sample analyses to identify outliers with the most weight in the MR analysis and the largest contribution to Cochran’s Q statistic for heterogeneity, which were then removed and the data reanalysed. Diagnostic scatter plots were generated to assess the presence of pleiotropy further graphically. A full description and rationale for selecting sensitivity analysis can be found in supplementary methods. MR analysis was performed with R v4.3.1 using the “TwoSampleMR” and “MR.raps” packages. False-discovery rate (FDR) was used to correct for multiple testing (P_Lipids_-_FDR_ = 0.035 and P_drug proxy-FDR_ = 0.013).

## Results

### Systematic review and meta-analysis

The searches yielded 7503 citations after removing 1741 duplicates. After reviewing the titles and abstracts, 7491 articles were excluded (Figure S8). Of the remaining 12 studies, eight were removed after full-text screening. Four studies (three cohorts [10, 11, 48] and one case–control [49]) investigating the influence of lipids and apolipoproteins on the risk of developing ALS were included, and none related to FTD (Table 2).

**Table 2:** Overview of included studies in the systematic review and meta-analysis

Author	Year	Country	Study Design	No of patients	Age of cases	Exposure	Outcome	Adjustments	Risk of bias score*
Mariosa, D	2017	Sweden	Cohort	636,132	Mean (SD): 53 (67)	LDL, HDL, TC, TG, ApoA1, ApoB	ALS	Sex, age at first blood sampling, fasting status, occupation, country of birth	9
Thompson, A	2022	UK	Cohort	427,427 to 469,710	Median (IQR): 62 (56.8–66)	LDL, HDL, TC, TG, ApoA1, ApoB	ALS	Age, sex, BMI, smoking, physical activity, statin use, cerebrovascular and cardiovascular diseases, creatinine, triglycerides, and HbA1c	8
Vaage, AM	2023	Norway	Cohort	353,673 to 626,538	Mean (SD): 44.2 (8.8)	LDL, HDL, TC, TG	ALS	Sex, age, birth cohort, health survey, other lipids, BMI, physical activity, and smoking status	8
Bjornevik, K	2020	USA	Case–control	547 (controls) 275 (cases)	Mean (SD): 64.6 (7.2)	LDL, HDL, TC, TG	ALS	Age, sex, fasting status, and time of blood draw, BMI, physical activity, smoking, alcohol intake, plasma urate levels, and use of cholesterol lowering drugs	7

*ALS* Amyotrophic lateral sclerosis, *LDL* Low-density lipoprotein, *HDL* High-density lipoprotein, *TC* Total cholesterol, *TG* Triglycerides, *ApoA1* Apolipoprotein A1, *ApoB* Apolipoprotein B

^*^The quality of each study was rated using the following scoring algorithms: ≥ 7 points were considered as “good”, 2 to 6 points were considered as “fair”, and ≤ 1 point was considered as “poor” quality

**Fig. 2:**
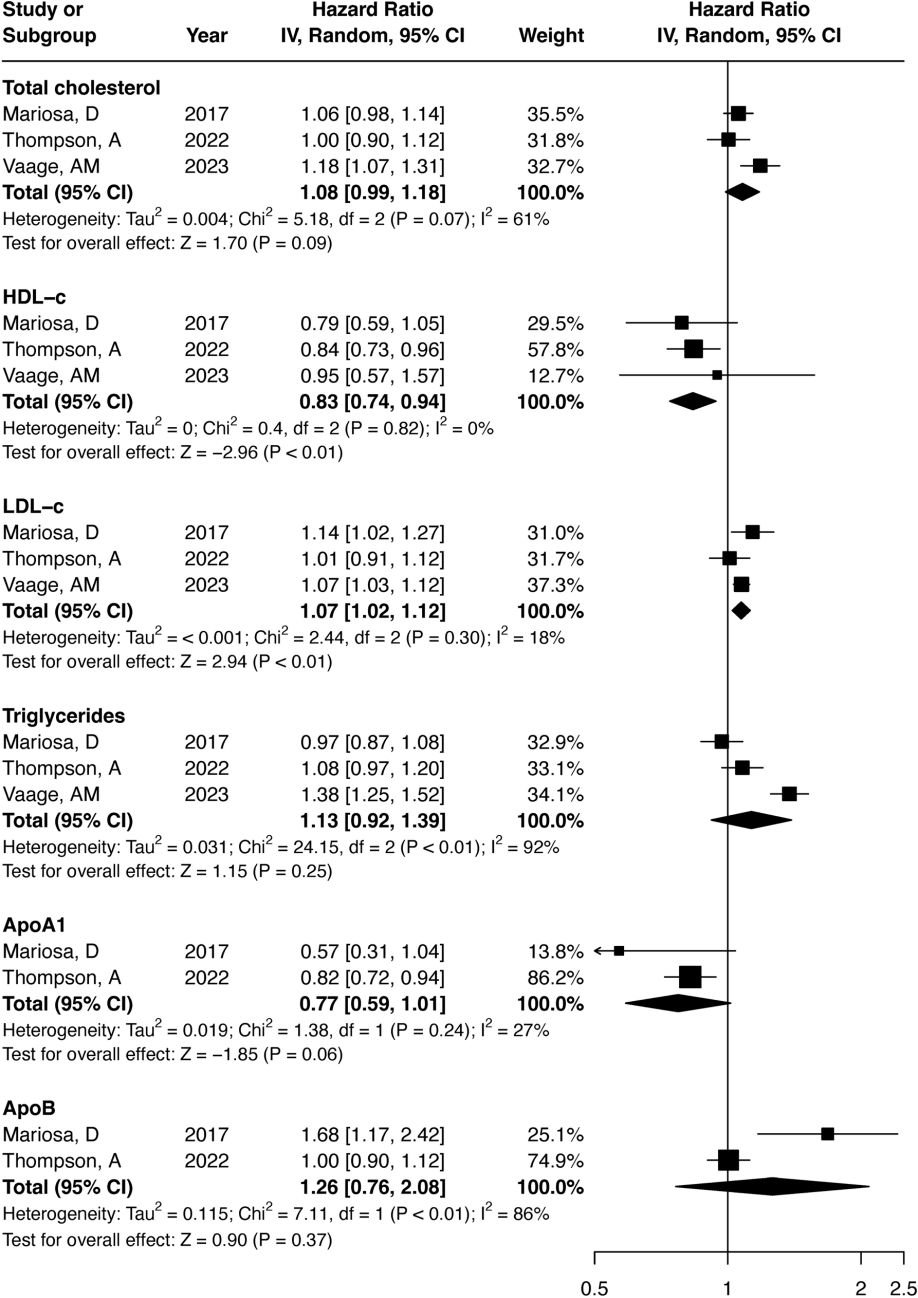
Meta-analysis of the reported HR across cohort studies in the literature for the association between lipids and apolipoproteins and the risk of ALS. The overall HR reflects the pooled HR for an increase of 1 mmol/L for lipids and 1 g/L for apolipoproteins

Amongst the cohort studies examining the impact of lipids on ALS, it was found that increased LDL-c levels were associated with a higher risk of ALS (HR_per 1 mmol/L_ = 1.07, 95%CI 1.02–1.12; ${I}^{2}=18\%$), whereas elevated levels of HDL-c were associated with a lower risk of ALS (HR_per 1 mmol/L_ = 0.83, 95%CI 0.74–0.94; ${I}^{2}=0\%$) (Fig. 2). No evidence for an association was found for the other lipids and apolipoproteins. The single case–control study reported a higher risk of ALS with higher levels of HDL-c (OR_per 1 SD_ = 1.22, 95%CI 1.04–1.43).

### Mendelian randomisation

#### Apolipoproteins, lipids, and risk of ALS and FTD

Three hundred and twenty-four independent SNPs associated with LDL-c, 290 SNPs associated with HDL-c, 333 SNPs associated with TC, 298 SNPs associated with TG, 11 SNPs associated with ApoA1, and 21 SNPs associated with ApoB were identified as IVs for lipid and apolipoprotein traits (Table S2).

**Fig. 3:**
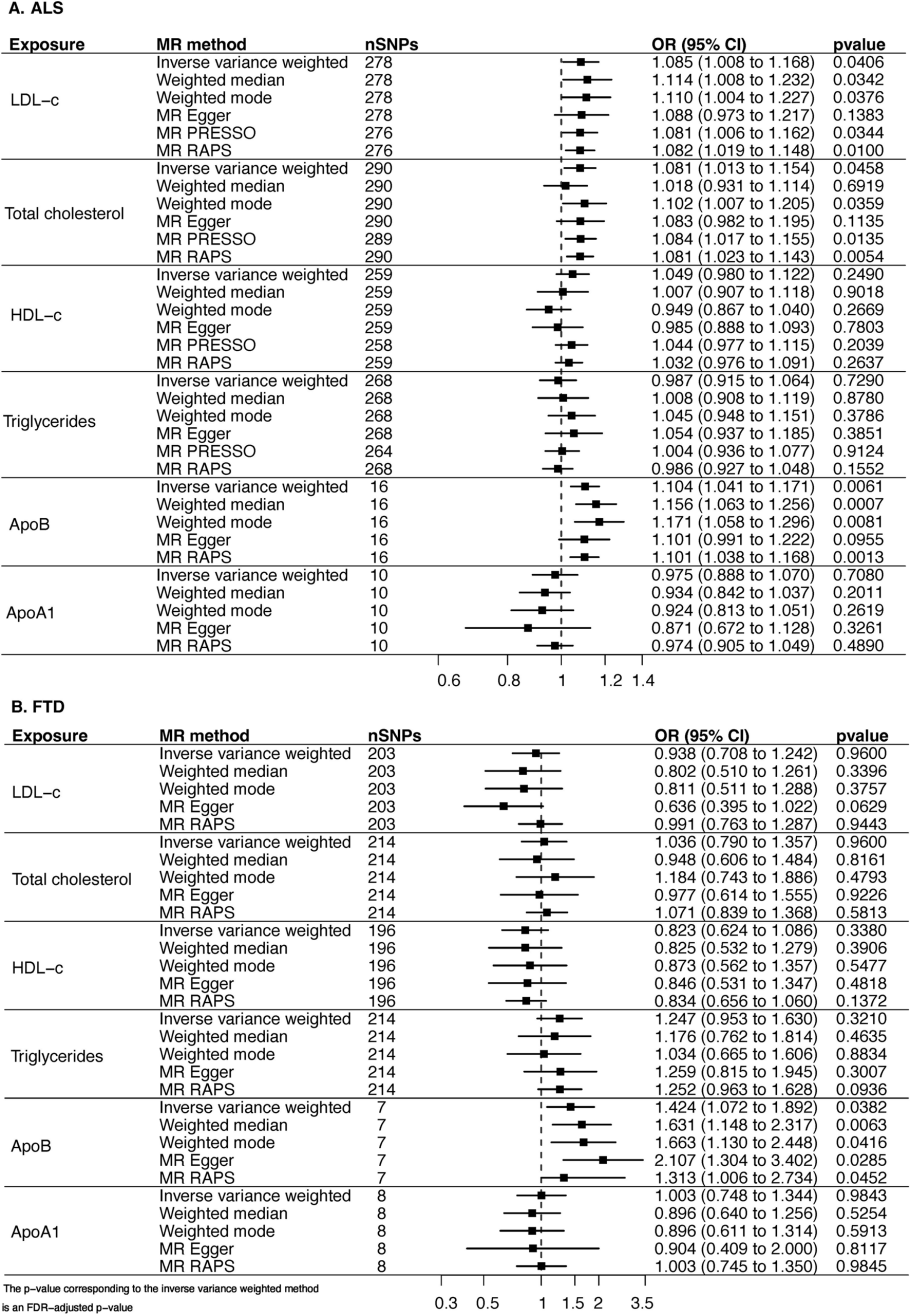
Univariate MR of the association of genetically predicted lipids and apolipoproteins levels with (**A**) ALS and (**B**) FTD. Forest plot of the association between a 1-SD change in the lipid and apolipoprotein levels with ALS and FTD risk. An effect size of > 1.00 suggests an increased risk of disease associated with lipid and apolipoprotein levels. The *p* value corresponding to the IVW method is an FDR-adjusted *p* value. *LDL-c* low-density lipoprotein cholesterol, *HDL-c* high-density lipoprotein cholesterol, *OR* odds ratio, *SNP* single-nucleotide polymorphisms, *ApoB* Apolipoprotein B, *ApoA1* Apolipoprotein A1

Increase in genetically proxied LDL-c (OR_IVW_ = 1.085, 95% CI 1.008–1.168, p_FDR_ = 4.06E-02) and TC (OR_IVW_ = 1.081, 95% CI 1.013–1.154, p_FDR_ = 4.58E-02) levels was associated with an increased risk of ALS (Fig. 3, Figures S9–S12). These findings were consistent in the weighted mode, MR-RAPS, and MR-PRESSO analyses. The weighted median analysis supported a potential causal effect of LDL-c on ALS but not TC although the effect size was in the same direction as IVW. MR-PRESSO identified one outlier SNP with horizontal pleiotropy. Reanalysis excluding this SNP was consistent with the primary analysis. Due to evidence of heterogeneity (Table S3), potentially indicating violations of MR assumptions, we used radial plots to aid in detecting outlying variants (Figures S13–S16). Radial MR analysis identified 30 outliers for LDL-c, 27 for HDL-c, 24 for TC, and 23 for TG in inverse variance weighted (Table S4). Results were unchanged following the exclusion of these SNPs. MR-Egger was consistent with IVW in direction, but the estimate was not significant; the Egger intercept was not significantly different from zero.

A similar relationship was observed for genetically predicted ApoB on ALS (OR_IVW_ = 1.104, 95% CI 1.041–1.171, p_FDR_ = 6.12E-03). MR-Egger showed a direction of effect consistent with IVW, but the estimate was not significant. Egger intercept was not significantly different from zero. All other sensitivity analyses agreed (Fig. 3, Figures S17–S18).

No association was found between the examined lipid traits and ALS survival (Figure S19, Figures S20–S25); however, this analysis had 80% power to detect a relatively large minimum effect of OR = 1.40 (or 0.70).

Amongst the examined lipids and apolipoproteins, only genetically proxied ApoB level was positively associated with FTD (Fig. 3, Figures S26–S31), supported by both the primary analysis (OR_IVW_ = 1.424, 95% CI 1.072–1.829, p_FDR_ = 3.82E-02) and sensitivity analyses.

#### Lipid-lowering drug targets and risk of ALS and FTD

The positive control analyses identified significant associations between genetically proxied drug targets and a decreased risk of CAD, ensuring the efficacy of the genetic instruments (Table S5). F statistics for the respective genetic instruments ranged from 10.56 to 6,388.6, suggesting that weak instrument bias was unlikely to affect the analyses (Table S2).

**Fig. 4:**
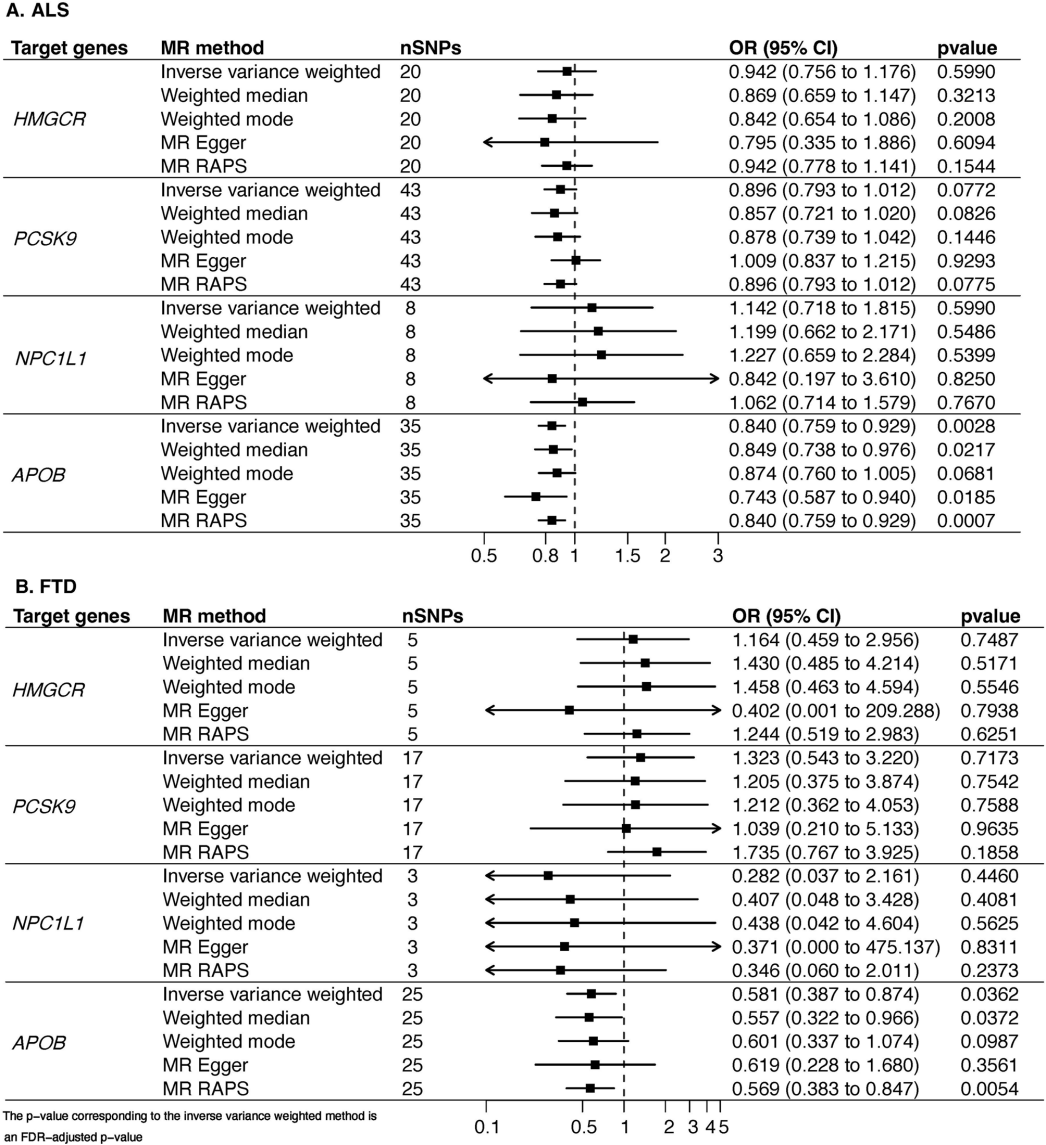
Univariate MR of the association of genetically proxied lipid-lowering drug targets with (**A**) ALS and (**B**) FTD. Forest plot of the association between a 1-SD change in the LDL-c levels of four lipid-lowering drug targets with ALS and FTD risk. An effect size of < 1.00 suggests a decreased risk of disease associated with lipid-lowering drug treatment. The *p* value corresponding to the IVW method is an FDR-adjusted *p* value. *LDL-c* low-density lipoprotein cholesterol, *OR* odds ratio, *HR* hazard ratio, *SNP* single-nucleotide polymorphisms, *HMGCR* HMG-CoA reductase, *NPC1L1* Niemann–Pick C1-like protein 1, *PCSK9* proprotein convertase subtilisin/kexin type 9, *APOB* Apolipoprotein B-100

Reducing LDL-c through targeting *APOB* was significantly associated with lower risk of ALS (OR_IVW_ = 0.84, 95%CI 0.759–0.929, p_FDR_ = 2.75E-03) and FTD (OR_IVW_ = 0.581, 95%CI 0.387–0.874, p_FDR_ = 3.62E-02) (Fig. 4, Figures S32–S43). The results of sensitivity analyses using other methods were consistent, generating similar effect estimates for ALS (OR_WM_ = 0.849, 95% CI 0.738 to 0.929, *p* = 2.17E-02; OR_Egger_ = 0.743, 95% CI 0.759 to 0.940, *p* = 1.85E-02, OR_RAPS_ = 0.84, 95%CI 0.759–0.929, *p* = 6.90E-04) and FTD (OR_WM_ = 0.557, 95% CI 0.322–0.966, *p* = 3.72E-02; OR_RAPS_ = 0.569, 95% CI 0.383–0.847, *p* = 5.40E-03). MR-PRESSO did not identify any outlying instrumental variables, and the intercepts of MR-Egger regression did not indicate evidence of horizontal pleiotropy for all analyses (Table S3). There was no evidence of any effect on ALS survival (Figure S44).

## Discussion

Meta-analysis of three cohort studies indicated that elevations in LDL-c and HDL-c are associated with increased and decreased risk of ALS, respectively. No observational studies examining lipid traits and FTD risk were found. In support of a causal role for the observed association between LDL-c and ALS risk, two-sample MR analysis provided evidence of a potential causal association between genetically predicted higher levels of LDL-c, TC and ApoB and risk of ALS, and of higher ApoB levels and FTD risk. However, no association was identified for HDL-c or ApoA1 in relation to ALS or FTD. Using genetic proxies for lipid-lowering therapies targeting four individual genes, this study suggests that reducing LDL-c levels through targeting of *APOB* (a genetic proxy for Mipomersen treatment) could reduce the risk of ALS and FTD. All findings were robust to extensive sensitivity analyses. In relation to the effects of lipid biomarkers on the aggressiveness of ALS, no evidence of a causal association between lipid biomarkers or cholesterol-targeting therapies and survival in ALS was found.

The extant literature examining the role of lipids and apolipoproteins influencing the development of ALS and FTD is limited. One finding of our meta-analysis, that elevated levels of LDL-c are associated with increased risk of ALS, is supported by causal evidence from our MR study. Previous MR studies have reported similar results, highlighting the negative effect of LDL-c [13, 50]. We did not find a statistically significant association between ApoB and ALS in the meta-analysis of two cohort studies, which contrasts with our MR analysis and previous genetic epidemiological studies [50]. This might be explained by the high heterogeneity in the meta-analysis (86%), the relationship between ApoB and LDL-c—since ApoB is the major apolipoprotein constituent of a range of circulating lipid particles beyond LDL-c—or potential alterations in lipid biomarkers that occur before the onset of symptomatic ALS, influencing the associations in observational studies [51].

The second finding of our meta-analysis, that lower levels of HDL-c are associated with a higher risk of ALS, was not supported by causal evidence from MR. One case–control study [49] also identified an inverse association, where higher HDL-c increased the risk of ALS, which could be explained by reverse causation, as case–control studies are more susceptible to this, along with variations in population characteristics, measurement methods, and confounding factors that may contribute to the conflicting findings. This is in keeping with prior genetic epidemiological studies, which have also failed to show evidence of a causal role for HDL-c in ALS risk [13]. This parallels findings in cardiovascular disease prevention, in which the robust association between lower HDL-c and higher risk of cardiovascular disease using classical epidemiological methods has not been supported by genetic epidemiological analysis or randomised control trials of treatments aiming to increase HDL-c levels [52, 53]. There is some evidence for very high levels of HDL-c increasing the risk of cardiovascular disease [54], but it is not clear that this explains the discrepancy between established observational and genetic epidemiological findings, the cause of which remains enigmatic. It may, therefore, indicate that HDL-c levels have a role in the prediction of ALS risk but do not themselves mediate that association.

Though no observational studies were identified that examine FTD risk in relation to lipid biomarkers, our MR analysis provides evidence that elevated levels of ApoB increase the risk of FTD. Despite the high correlation between ApoB and LDL-c levels, no causal association was identified between LDL-c and FTD risk. Although all MR analyses of FTD risk were of lower power than analyses of ALS due to the much smaller sample size and SNP coverage in the FTD GWAS, the near-zero association of LDL-c and FTD risk and higher estimated power to detect an effect for LDL-c suggest that insufficient power does not explain this discrepancy.

Multiple observational studies have tied metabolic factors during the course of symptomatic ALS with differences in disease progression and survival, including an association between lower levels of HDL-c and shorter survival, higher levels of TG, TC and LDL-c and longer survival [14–18]. Our MR analysis did not find evidence of a causal relationship between any of the lipid species investigated and survival in ALS. This might be attributable to the lower power to detect a small effect as a consequence of the small sample size of the outcome GWAS of survival in people with ALS, as well as that ALS survival may not be very heritable and that the genetic determinants of lipid metabolism in health may differ from those in disease. However, our findings are in accordance with previous genetic epidemiological work in which polygenic scores for lipid levels were not associated with survival in ALS patients [18]. Observational associations between lipids and survival might be explicable due to systemic metabolic alterations occurring in more aggressive diseases or relate to dietary changes occurring because of the disease process, as opposed to directly influencing survival. Studies of the effect of lipids on the risk of FTD are limited. One previous genetic epidemiological study exploring this relationship did not find an association between lipids and risk of FTD (specifically FTD with TDP-43 pathology), perhaps owing to the smaller sample size and lower genomic coverage of the outcome GWAS [55].

Using genetic proxies for drug targets, we identified a potential effect of targeting APOB as a means to reduce the LDL-c levels and the risk of ALS and FTD. APOB is the major protein constituent of LDL-c, and levels of the two are highly correlated, but it is also the main protein constituent of other lipoprotein particles, including very low-density lipoprotein cholesterol and chylomicrons. APOB acts as a ligand for the activation of the LDL receptor [56]. The discrepancy between causal effects for LDL-c and ApoB levels on FTD risk is, therefore, somewhat unexpected, particularly given that estimates of statistical power (Figure S4) indicate higher power to detect a given effect for LDL-c compared with ApoB. A potential explanation for this discrepancy is that direct measurement of ApoB represents a more accurate measure of the concentration of lipoprotein particles, independent of the amount of cholesterol or other lipids per particle [57, 58]; again, this parallels cardiovascular disease in which the LDL particle number and ApoB levels more accurately reflect disease risk [58].

As a major constituent of the central nervous system with crucial roles in its normal functioning, there is great interest in defining the role of lipids in neurodegenerative disease [59]. Beyond ALS and FTD, dysfunctional cholesterol metabolism has been identified in Alzheimer’s, Parkinson’s and Huntington’s disease, implicating lipid pathways as a broad aetiological factor in neurodegenerative disease [60]. Since brain cholesterol is largely synthesised in situ without significant translocation of cholesterol from blood [61], it remains unclear how circulating lipids reflect neurodegenerative disease risk. Hypotheses that might explain this relationship include a role for oxidised cholesterol species [62], a means of transporting cholesterol between the central nervous system and circulation that are toxic to neurons, or cholesteryl esters [63], a means of sequestering excess cholesterol but which exert oxidative stress on neurons. Lipid levels may also reflect far more complex changes evolving at the synaptic level [64].

The small sample size of the GWAS of FTD and of survival in people with ALS are significant limitations. This has inevitably impacted the power of the analysis. ALS is a more pathologically homogeneous disease, with 97% of cases being associated with TDP-43 pathology, and only 50% of FTD cases are associated with TDP-43 pathology. Although exposures such as alterations in blood lipids might have an effect on FTD risk that is not pathology-specific, any pathology-specific effect would negatively impact the power of this analysis. No GWAS of sufficient size examining pathological subtypes of FTD exists to probe this question. Limitations relating to the genetic proxies of therapeutic targets include that genetic variants reflect the effect of lifelong changes in lipid levels on ALS or FTD risk, and the magnitude of the effect may not be comparable with the short-term effects of lipid-lowering drugs. Our study only predicts the on-target effects of specific drug targets, and these models do not estimate potential off-target effects. Horizontal pleiotropy cannot be completely excluded, although various sensitivity analyses were performed to test the assumptions of MR analyses. Our analyses also assume no gene–environment or gene–gene interactions and linear and time-dependent effects of drug targets on ALS or FTD risk. Furthermore, since our findings were limited to GWAS of individuals of European ancestry, these findings are not necessarily valid for other genetic ancestries.

In conclusion, these data support a causal role for higher LDL-c and total cholesterol increasing the risk of ALS and higher APOB increasing the risk of both ALS and FTD. The findings reveal the potential for *APOB* inhibitors to reduce the risk of sporadic ALS and FTD. Further work in monogenic forms of ALS and FTD is necessary to determine whether reducing blood lipids influences risk in those at high risk. Understanding the mechanisms by which LDL-c and ApoB mediate ALS and FTD risk may help identify additional approaches to the prevention of these diseases.

## Supplementary Information

Below is the link to the electronic supplementary material.

Supplementary file1 (DOCX 6460 KB)

## Data Availability

All data are publicly available. Detailed information for these datasets is summarised in Table S1.
