# Supplementary material for: Apolipoproteins, lipids, lipid-lowering drugs and risk of amyotrophic lateral sclerosis and frontotemporal dementia: a meta-analysis and Mendelian randomisation study
Source: J Neurol. 2024 Sep 4;271(10):6956–69. doi: 10.1007/s00415-024-12665-x (PMC11447100; doi:10.1007/s00415-024-12665-x)
Supplement: Supplementary file 1 — Supplementary file1 (DOCX 6460 KB) [file 415_2024_12665_MOESM1_ESM.docx]

**Apolipoproteins, lipids, lipid-lowering drugs and risk of amyotrophic lateral sclerosis and frontotemporal dementia: A meta-analysis and Mendelian randomisation study**

**Short title**: Lipid Biomarkers and Risk of ALS and FTD

**Authors**

Christos V. Chalitsios, Harriet Ley, Jiali Gao, Martin R Turner, Alexander G. Thompson

**Supplements**

[**Figure S1**. Overview and assumptions of the MR design. Dashed lines represent potential pleiotropic or direct causal effects between variables that would violate Mendelian randomisation assumptions. Assumption 1: Genetic variants are associated with the exposure; Assumption 2: Genetic variants are not associated with any confounders; and Assumption 3: Genetic variants influence risk only through the exposure and not through any alternative pathways. The MR design can reduce residual confounding and reverse causality, thereby reinforcing the causal inference of an exposure-outcome association. This is because genetic variants, selected as instrumental variables for studying the effect of modifying the exposure, are randomly allocated at conception and, therefore, less vulnerable to confounding from environmental factors and reverse causation. 4](#_Toc160114675)

[**Figure S2**. Minimum detecTable S odds ratio at a range of power thresholds for the Mendelian randomisation analyses of lipid and apolipoprotein traits on ALS. 4](#_Toc160114676)

[**Figure S3**. Minimum detecTable S hazard ratio at a range of power thresholds for the Mendelian randomisation analyses of lipid and apolipoprotein traits on ALS survival. 5](#_Toc160114677)

[**Figure S4**. Minimum detecTable S odds ratio at a range of power thresholds for the Mendelian randomisation analyses of lipid and apolipoprotein traits on ALS. 5](#_Toc160114678)

[**Figure S5**. Minimum detecTable S odds ratio at a range of power thresholds for the Mendelian randomisation analyses of genetic proxies of lipid-lowering drugs on ALS. 6](#_Toc160114679)

[**Figure S6**. Minimum detecTable S odds ratio at a range of power thresholds for the Mendelian randomisation analyses of genetic proxies of lipid-lowering drugs on ALS survival. 6](#_Toc160114680)

[**Figure S7**. Minimum detecTable S odds ratio at a range of power thresholds for the Mendelian randomisation analyses of genetic proxies of lipid-lowering drugs on FTD. 7](#_Toc160114681)

[**Figure S8**. Scatter plot of individual SNP-ALS and SNP-LDL-c associations with an overlay of the causal estimate from each MR test in two-sample MR analysis. 9](#_Toc160114682)

[**Figure S9**. Scatter plot of individual SNP-ALS and SNP-HDL-c associations with an overlay of the causal estimate from each MR test in two-sample MR analysis. 9](#_Toc160114683)

[**Figure S10**. Scatter plot of individual SNP-ALS and SNP-total cholesterol associations with an overlay of the causal estimate from each MR test in two-sample MR analysis. 10](#_Toc160114684)

[**Figure S11.** Scatter plot of individual SNP-ALS and SNP-triglycerides associations with an overlay of the causal estimate from each MR test in two-sample MR analysis. 10](#_Toc160114685)

[**Figure S12**. Radial curve displays the ratio estimate for each genetic variant, as well as the overall IVW (in blue) and MR Egger estimate (in orange) between LDL-c and ALS. Data points with large contributions to Cochran’s Q statistic are shown in pink, with an alpha of 0.05/nSNPs. 12](#_Toc160114686)

[**Figure S13**. Radial curve displays the ratio estimate for each genetic variant, as well as the overall IVW (in blue) and MR Egger estimate (in orange) between HDL-c and ALS. Data points with large contributions to Cochran’s Q statistic are shown in pink, with an alpha of 0.05/nSNPs. 13](#_Toc160114687)

[**Figure S14**. Radial curve displays the ratio estimate for each genetic variant, as well as the overall IVW (in blue) and MR Egger estimate (in orange) between total cholesterol and ALS. Data points with large contributions to Cochran’s Q statistic are shown in pink, with an alpha of 0.05/nSNPs. 13](#_Toc160114688)

[**Figure S15**. Radial curve displays the ratio estimate for each genetic variant, as well as the overall IVW (in blue) and MR Egger estimate (in orange) between triglycerides and ALS. Data points with large contributions to Cochran’s Q statistic are shown in pink, with an alpha of 0.05/nSNPs. 14](#_Toc160114689)

[**Figure S16**. Scatter plot of individual SNP-ALS and SNP-ApoA1 associations with an overlay of the causal estimate from each MR test in two-sample MR analysis. 17](#_Toc160114690)

[**Figure S17**. Scatter plot of individual SNP-ALS and SNP-ApoB associations with an overlay of the causal estimate from each MR test in two-sample MR analysis. 17](#_Toc160114691)

[**Figure S18**. Scatter plot of individual SNP-ALS survival and SNP-LDL-c associations with an overlay of the causal estimate from each MR test in two-sample MR analysis 18](#_Toc160114692)

[**Figure S19**. Scatter plot of individual SNP-ALS survival and SNP-HDL-c associations with an overlay of the causal estimate from each MR test in two-sample MR analysis. 18](#_Toc160114693)

[**Figure S20**. Scatter plot of individual SNP-ALS survival and SNP-total cholesterol associations with an overlay of the causal estimate from each MR test in two-sample MR analysis. 19](#_Toc160114694)

[**Figure S21**. Scatter plot of individual SNP-ALS survival and SNP-triglycerides associations with an overlay of the causal estimate from each MR test in two-sample MR analysis. 19](#_Toc160114695)

[**Figure S22**. Scatter plot of individual SNP-ALS survival and SNP-ApoA1 associations with an overlay of the causal estimate from each MR test in two-sample MR analysis. 20](#_Toc160114696)

[**Figure S23**. Scatter plot of individual SNP-ALS survival and SNP-ApoB associations with an overlay of the causal estimate from each MR test in two-sample MR analysis. 20](#_Toc160114697)

[**Figure S24**. Scatter plot of individual SNP-FTD and SNP-LDL-c associations with an overlay of the causal estimate from each MR test in two-sample MR analysis. 21](#_Toc160114698)

[**Figure S25**. Scatter plot of individual SNP-FTD and SNP-HDL-c associations with an overlay of the causal estimate from each MR test in two-sample MR analysis. 21](#_Toc160114699)

[**Figure S26**. Scatter plot of individual SNP-FTD and SNP-total cholesterol associations with an overlay of the causal estimate from each MR test in two-sample MR analysis. 22](#_Toc160114700)

[**Figure S27**. Scatter plot of individual SNP-FTD and SNP-triglycerides associations with an overlay of the causal estimate from each MR test in two-sample MR analysis. 22](#_Toc160114701)

[**Figure S28.** Scatter plot of individual SNP-FTD and SNP-ApoA1 associations with an overlay of the causal estimate from each MR test in two-sample MR analysis. 23](#_Toc160114702)

[**Figure S29**. Scatter plot of individual SNP-FTD and SNP-ApoB associations with an overlay of the causal estimate from each MR test in two-sample MR analysis. 23](#_Toc160114703)

[**Figure S30.** Scatter plot of individual SNP-ALS and SNP-genetically proxied HMGCR inhibition associations with an overlay of the causal estimate from each MR test in two-sample MR analysis. 25](#_Toc160114704)

[**Figure S31**. Scatter plot of individual SNP-ALS and SNP-genetically proxied PCSK9 inhibition associations with an overlay of the causal estimate from each MR test in two-sample MR analysis. 25](#_Toc160114705)

[**Figure S32**. Scatter plot of individual SNP-ALS and SNP-genetically proxied NPC1L1 inhibition associations with an overlay of the causal estimate from each MR test in two-sample MR analysis. 26](#_Toc160114706)

[**Figure S33.** Scatter plot of individual SNP-ALS and SNP-genetically proxied APOB inhibition associations with an overlay of the causal estimate from each MR test in two-sample MR analysis. 26](#_Toc160114707)

[**Figure S34**. Scatter plot of individual SNP-ALS survival and SNP-genetically proxied HMGCR inhibition associations with an overlay of the causal estimate from each MR test in two-sample MR analysis. 27](#_Toc160114708)

[**Figure S35.** Scatter plot of individual SNP-ALS survival and SNP-genetically proxied PCSK9 inhibition associations with an overlay of the causal estimate from each MR test in two-sample MR analysis. 27](#_Toc160114709)

[**Figure S36**. Scatter plot of individual SNP-ALS and SNP-genetically proxied NPC1L1 inhibition associations with an overlay of the causal estimate from each MR test in two-sample MR analysis. 28](#_Toc160114710)

[**Figure S37**. Scatter plot of individual SNP-ALS survival and SNP-genetically proxied APOB inhibition associations with an overlay of the causal estimate from each MR test in two-sample MR analysis. 28](#_Toc160114711)

[**Figure S38**. Scatter plot of individual SNP-FTD and SNP-genetically proxied HMGCR inhibition associations with an overlay of the causal estimate from each MR test in two-sample MR analysis. 29](#_Toc160114712)

[**Figure S39.** Scatter plot of individual SNP-FTD and SNP-genetically proxied PCSK9 inhibition associations with an overlay of the causal estimate from each MR test in two-sample MR analysis. 29](#_Toc160114713)

[**Figure S40**. Scatter plot of individual SNP-FTD and SNP-genetically proxied NPC1L1 inhibition associations with an overlay of the causal estimate from each MR test in two-sample MR analysis. 30](#_Toc160114714)

[**Figure S41**. Scatter plot of individual SNP-FTD and SNP-genetically proxied APOB inhibition associations with an overlay of the causal estimate from each MR test in two-sample MR analysis. 30](#_Toc160114715)

[**Table S 1**. Systematic review search terms 3](#_Toc162618268)

[**Table S 2**. Characteristic of the genome-wide association studies used to create instrumental variables. 8](#_Toc162618269)

[**Table S 3**. Heterogeneity and pleiotropy tests of instrument effects. 11](#_Toc162618270)

[**Table S 4**. Identified outliers with the most weight in the MR analysis and the largest contribution to Cochran’s Q statistic for heterogeneity from the MR Radial method. 15](#_Toc162618271)

[**Table S 5**. Univariate MR of the association of genetically proxied lipid-lowering drug targets with coronary artery disease (positive control analysis) 25](#_Toc162618272)

**Supplementary methods – Mendelian randomisation**

Inverse variance–weighted (IVW) MR, returning an unbiased estimate if the horizontal pleiotropy is balanced (1). To account for potential horizontal pleiotropy, several MR sensitivity analyses were performed, each providing a valid MR estimate under different combinations of assumptions. MR-Egger provides an unbiased causal effect estimate even if the third MR assumption (that there is an absence of horizontal pleiotropy) is violated and all the variants are invalid IVs, provided that the Instrument Strength Independent of Direct Effect (InSIDE) assumption of independence between the horizontal pleiotropic effects and the variants-exposure effects are met (2). The weighted median returns a valid estimate when at least 50% of the weight of the genetic variants comes from valid IVs (3). The weighted mode is valid under the Zero Modal Pleiotropy Assumption (ZEMPA), according to which, out of all clusters of variants with similar effects, the largest is the group of valid genetic variants (4). To detect potential outlying IVs, we implemented the MR pleiotropy residual sum and outlier test (MR-PRESSO), which identifies and excludes outliers, applying a random-effects IVW model (5). In addition, MR using a robust adjusted profile score (MR-RAPS) (6), was used to control for pleiotropy through a random effects model, considering the variance in instrument effect sizes. When there was evidence of heterogeneity (Cochran’s Q statistic p-value >0.05), Radial MR analysis was performed (7) in the two sample analyses to identify outliers with the most weight in the MR analysis and the largest contribution to Cochran’s Q statistic for heterogeneity, which were then removed and the data reanalysed. Radial MR analysis was conducted using modified second-order weights and an α level of 0.05 divided by the number of SNPs being used to instrument the exposure.

**References**

1. Bowden J, Del Greco M F, Minelli C, Davey Smith G, Sheehan N, Thompson J. A framework for the investigation of pleiotropy in two‐sample summary data Mendelian randomization. Stat Med. 2017 May 20;36(11):1783–802.

2. Bowden J, Davey Smith G, Burgess S. Mendelian randomization with invalid instruments: effect estimation and bias detection through Egger regression. Int J Epidemiol. 2015 Apr 1;44(2):512–25.

3. Bowden J, Davey Smith G, Haycock PC, Burgess S. Consistent Estimation in Mendelian Randomization with Some Invalid Instruments Using a Weighted Median Estimator. Genet Epidemiol. 2016 May;40(4):304–14.

4. Hartwig FP, Davey Smith G, Bowden J. Robust inference in summary data Mendelian randomization via the zero modal pleiotropy assumption. Int J Epidemiol. 2017 Dec 1;46(6):1985–98.

5. Verbanck M, Chen CY, Neale B, Do R. Detection of widespread horizontal pleiotropy in causal relationships inferred from Mendelian randomization between complex traits and diseases. Nat Genet. 2018 May;50(5):693–8.

6. Zhao Q, Wang J, Hemani G, Bowden J, Small DS. Statistical inference in two-sample summary-data Mendelian randomization using robust adjusted profile score. Ann Stat. 2020;48(3):1742–69.

7. Bowden J, Spiller W, Del Greco M F, Sheehan N, Thompson J, Minelli C, et al. Improving the visualization, interpretation and analysis of two-sample summary data Mendelian randomization via the Radial plot and Radial regression. Int J Epidemiol. 2018 Aug 1;47(4):1264–78.


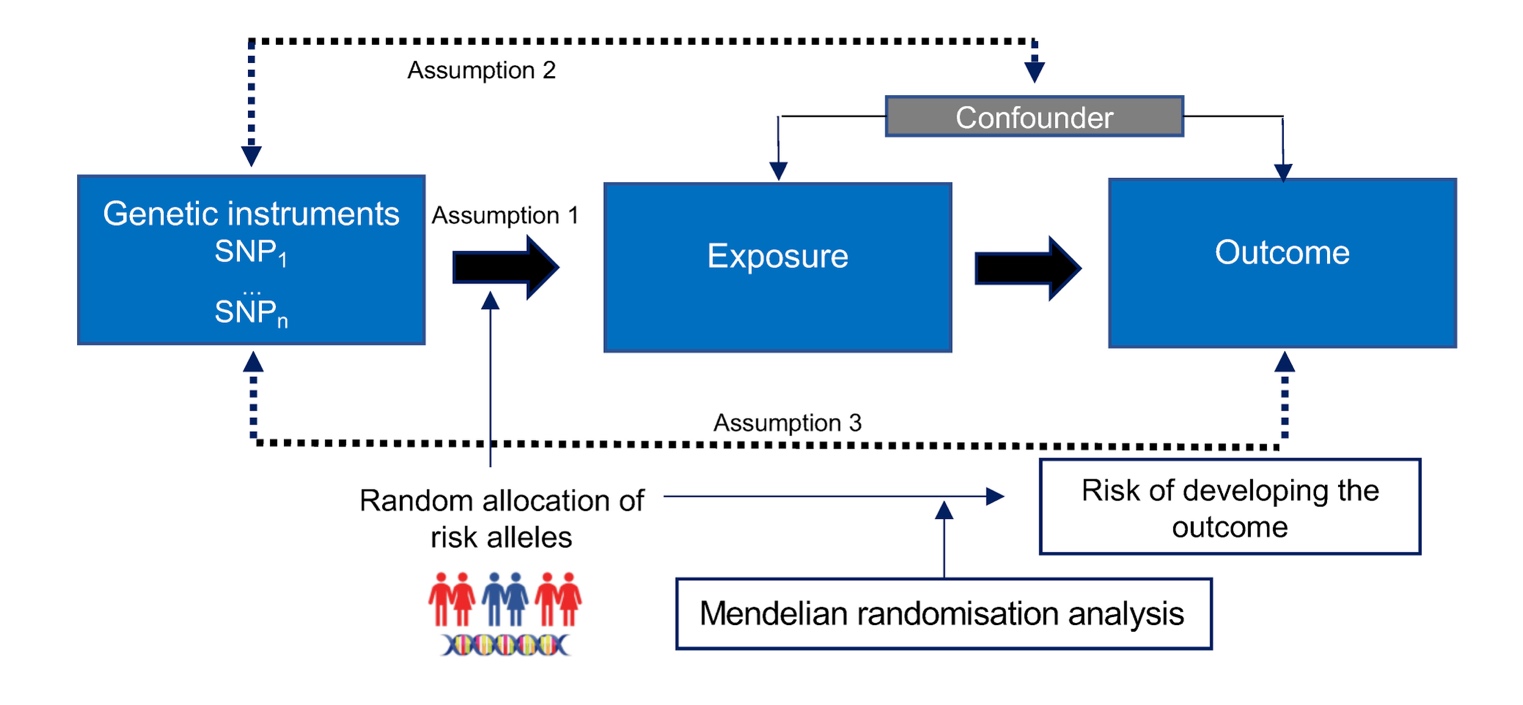


**Figure S1**. Overview and assumptions of the MR design. Dashed lines represent potential pleiotropic or direct causal effects between variables that would violate Mendelian randomisation assumptions. Assumption 1: Genetic variants are associated with the exposure; Assumption 2: Genetic variants are not associated with any confounders; and Assumption 3: Genetic variants influence risk only through the exposure and not through any alternative pathways. The MR design can reduce residual confounding and reverse causality, thereby reinforcing the causal inference of an exposure-outcome association. This is because genetic variants, selected as instrumental variables for studying the effect of modifying the exposure, are randomly allocated at conception and, therefore, less vulnerable to confounding from environmental factors and reverse causation.

**Table S1**. Systematic review search terms

| **Literature database** | **Search terms** |
| --- | --- |
| **PUBMED (MEDLINE)** | (amyotrophic lateral sclerosis OR Progressive muscular atrophy OR progressive bulbar palsy OR primary lateral sclerosis OR flail arm syndrome OR flail leg syndrome OR frontotemporal dementia OR Pick's disease OR motor neuron disease) **AND**  (total cholesterol OR cholesterol OR high density lipoprotein OR HDL OR low density lipoprotein OR LDL OR triglyceride OR lipid OR apolipoprotein A1 OR ApoA1 OR apolipoprotein B OR ApoB) |
| **EMBASE** | (amyotrophic lateral sclerosis or Progressive muscular atrophy or progressive bulbar palsy or primary lateral sclerosis or flail arm syndrome or flail leg syndrome or frontotemporal dementia or Pick's disease or motor neuron disease) **AND**  (total cholesterol OR cholesterol OR high density lipoprotein OR HDL OR low density lipoprotein OR LDL OR triglyceride OR lipid OR apolipoprotein A1 OR ApoA1 OR apolipoprotein B OR ApoB) |
| **Web of Science** | TS=(amyotrophic lateral sclerosis OR Progressive muscular atrophy OR progressive bulbar palsy OR primary lateral sclerosis OR flail arm syndrome OR flail leg syndrome OR frontotemporal dementia OR Pick's disease OR motor neuron disease) and Preprint Citation Index (Exclude – Database) **AND**  (TS=(amyotrophic lateral sclerosis OR Progressive muscular atrophy OR progressive bulbar palsy OR primary lateral sclerosis OR flail arm syndrome OR flail leg syndrome OR frontotemporal dementia OR Pick's disease OR motor neuron disease)) AND TS=(total cholesterol OR cholesterol OR high density lipoprotein OR HDL OR low density lipoprotein OR LDL OR triglyceride OR lipid OR apolipoprotein A1 OR ApoA1 OR apolipoprotein B OR ApoB) and Preprint Citation Index (Exclude – Database) |


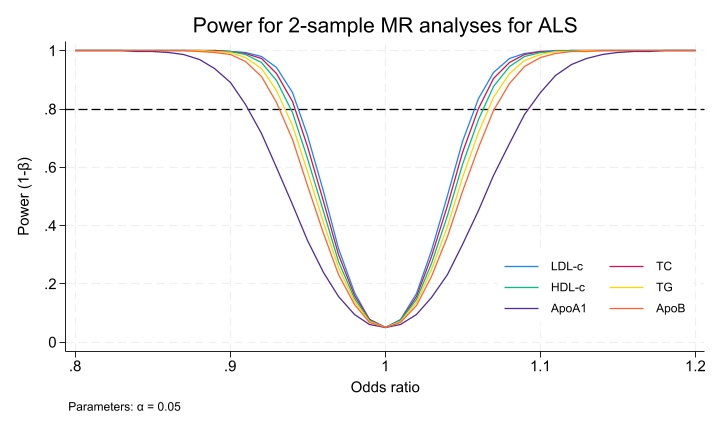


**Figure S2**. Minimum detecTable S odds ratio at a range of power thresholds for the Mendelian randomisation analyses of lipid and apolipoprotein traits on ALS.


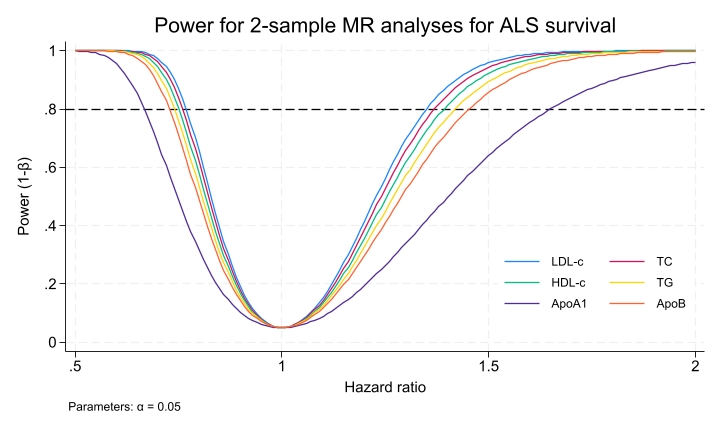


**Figure S3**. Minimum detecTable S hazard ratio at a range of power thresholds for the Mendelian randomisation analyses of lipid and apolipoprotein traits on ALS survival.


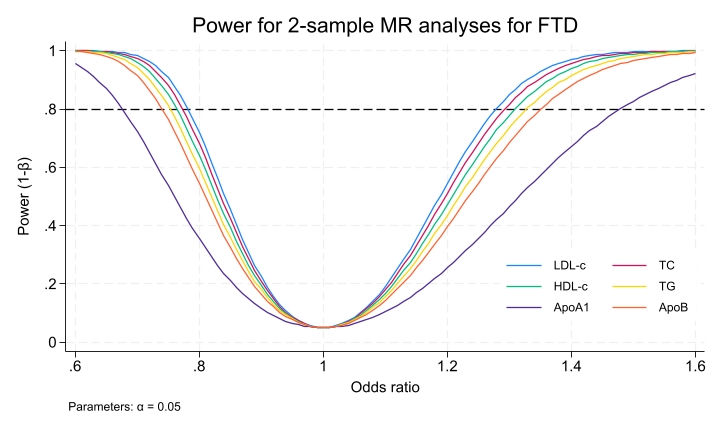


**Figure S4**. Minimum detecTable S odds ratio at a range of power thresholds for the Mendelian randomisation analyses of lipid and apolipoprotein traits on FTD.


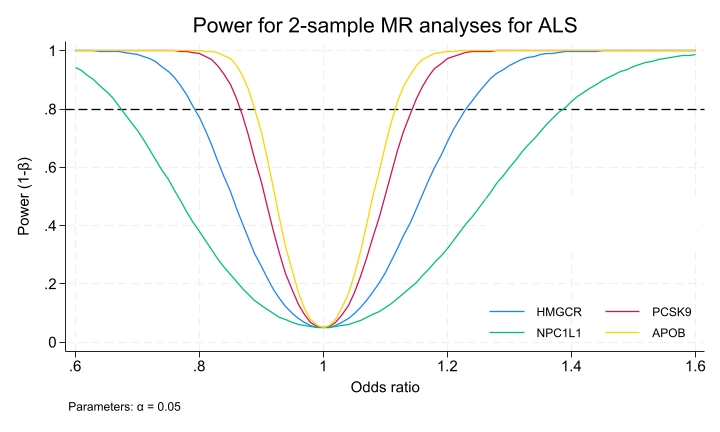


**Figure S5**. Minimum detecTable S odds ratio at a range of power thresholds for the Mendelian randomisation analyses of genetic proxies of lipid-lowering drugs on ALS.


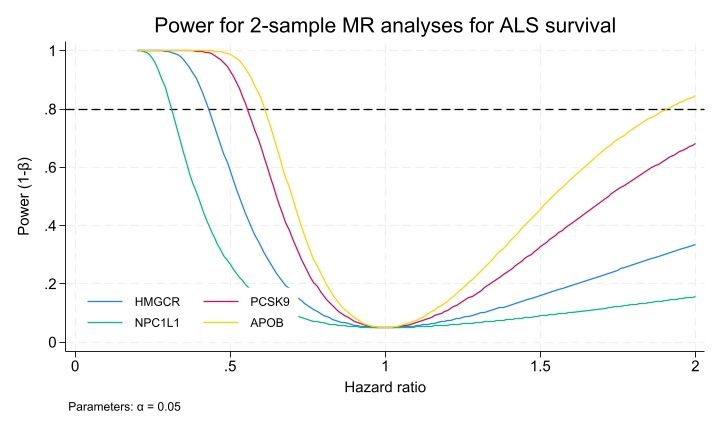


**Figure S6**. Minimum detecTable S odds ratio at a range of power thresholds for the Mendelian randomisation analyses of genetic proxies of lipid-lowering drugs on ALS survival.


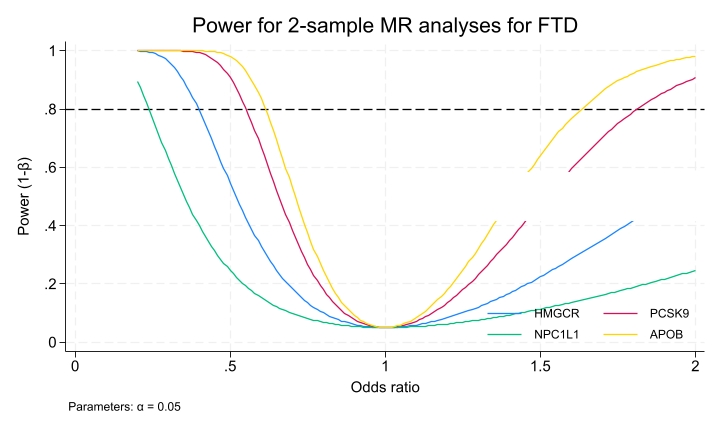


**Figure S7**. Minimum detecTable S odds ratio at a range of power thresholds for the Mendelian randomisation analyses of genetic proxies of lipid-lowering drugs on FTD.


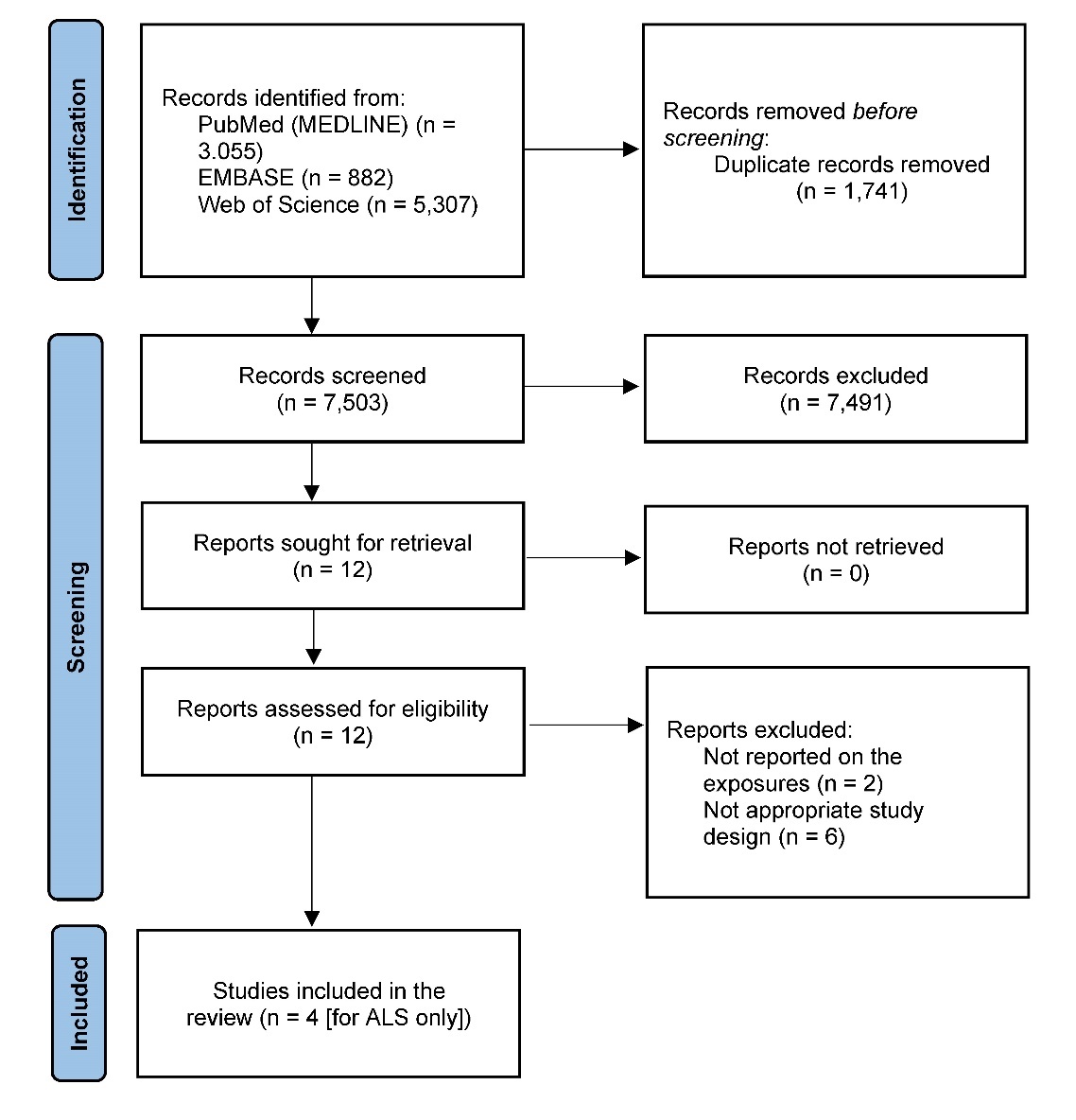


**Figure S8**. Study selection flowchart.

**Table S2**. Characteristic of the genome-wide association studies used to create instrumental variables.

| **Trait** | **PMID** | **Cases** | **Controls** | **Variance explained** | **F-statistic (range)** | **nSNPs** |
| --- | --- | --- | --- | --- | --- | --- |
| LDL-c | 34887591 | 1,231,284 | 21,543 | 0.12 | 40.27 to 6,388.88 | 324 |
| HDL-C | 34887591 | 1,244,546 | 397,564 | 0.10 | 51.14 to 10,212.66 | 290 |
| Total cholesterol | 34887591 | 1,320,010 | 12,228 | 0.11 | 43.11 to 5,143.32 | 333 |
| Triglycerides | 34887591 | 10,619 | 15,145 | 0.09 | 46.24 to 5825.17 | 298 |
| Apolipoprotein A1 | 27005778 | 3,162 | 294,770 | 0.047 | 32.23 to 284.79 | 11 |
| Apolipoprotein B | 27005778 | 17,416 | 375,455 | 0.09 | 31.59 to 270.85 | 21 |
| HMGCR | 34887591 | 1,231,284 | 21,543 | 0.008 | 12.4 to 2124.3 | 47 |
| PCSK9 | 34887591 | 1,231,284 | 21,543 | 0.02 | 28.9 to 6388.6 | 112 |
| NPC1L1 | 34887591 | 1,231,284 | 21,543 | 0.003 | 10.56 to 572 | 64 |
| APOB | 34887591 | 1,231,284 | 21,543 | 0.03 | 23.16 to 3441.1 | 62 |

LDL-c, low-density lipoprotein cholesterol; HDL-c, high-density lipoprotein cholesterol; HMGCR, HMG-CoA reductase; NPC1L1, Niemann-Pick C1-like protein 1; PCSK9, proprotein convertase subtilisin/kexin type 9; APOB, Apolipoprotein B-100.


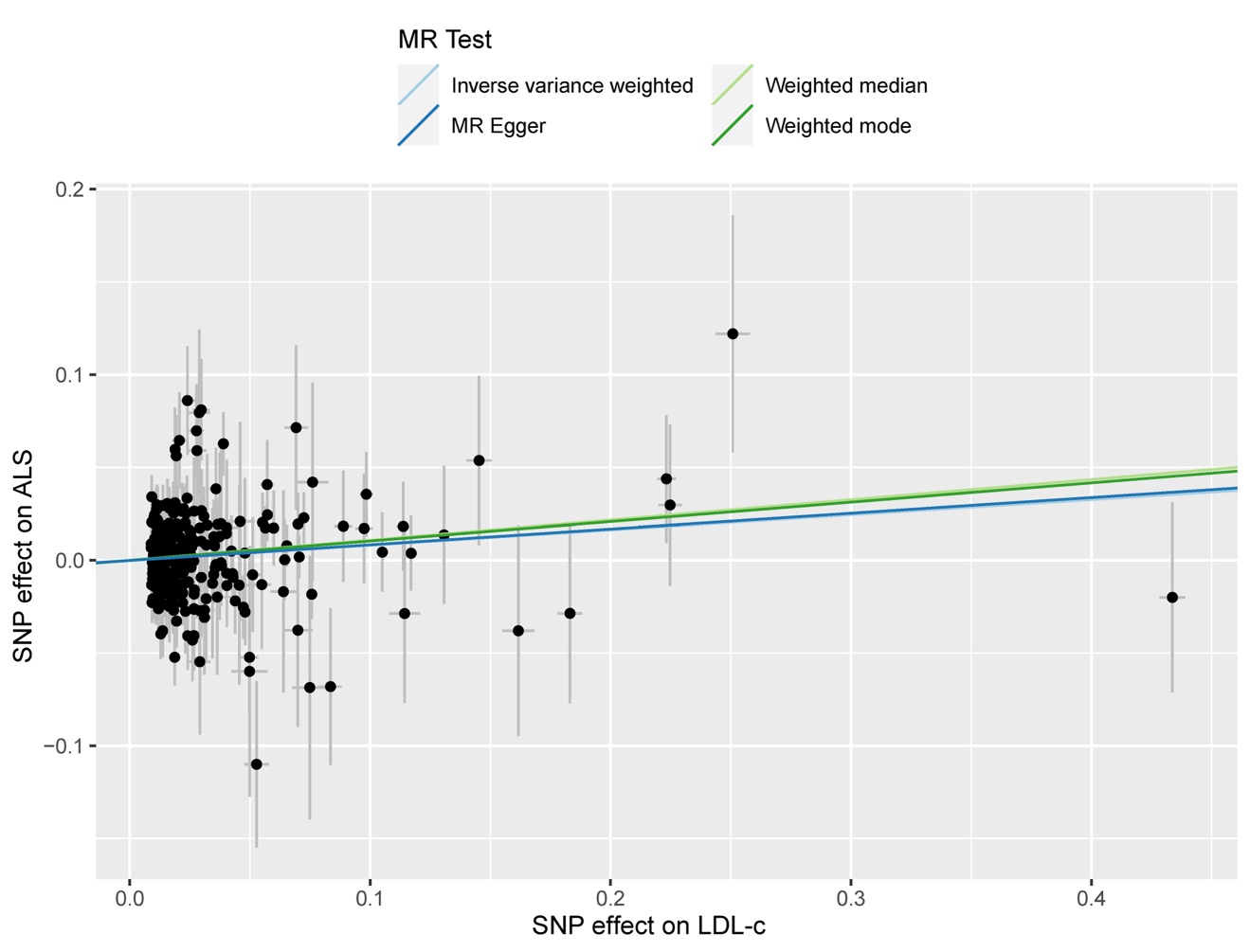


**Figure S9**. Scatter plot of individual SNP-ALS and SNP-LDL-c associations with an overlay of the causal estimate from each MR test in two-sample MR analysis.


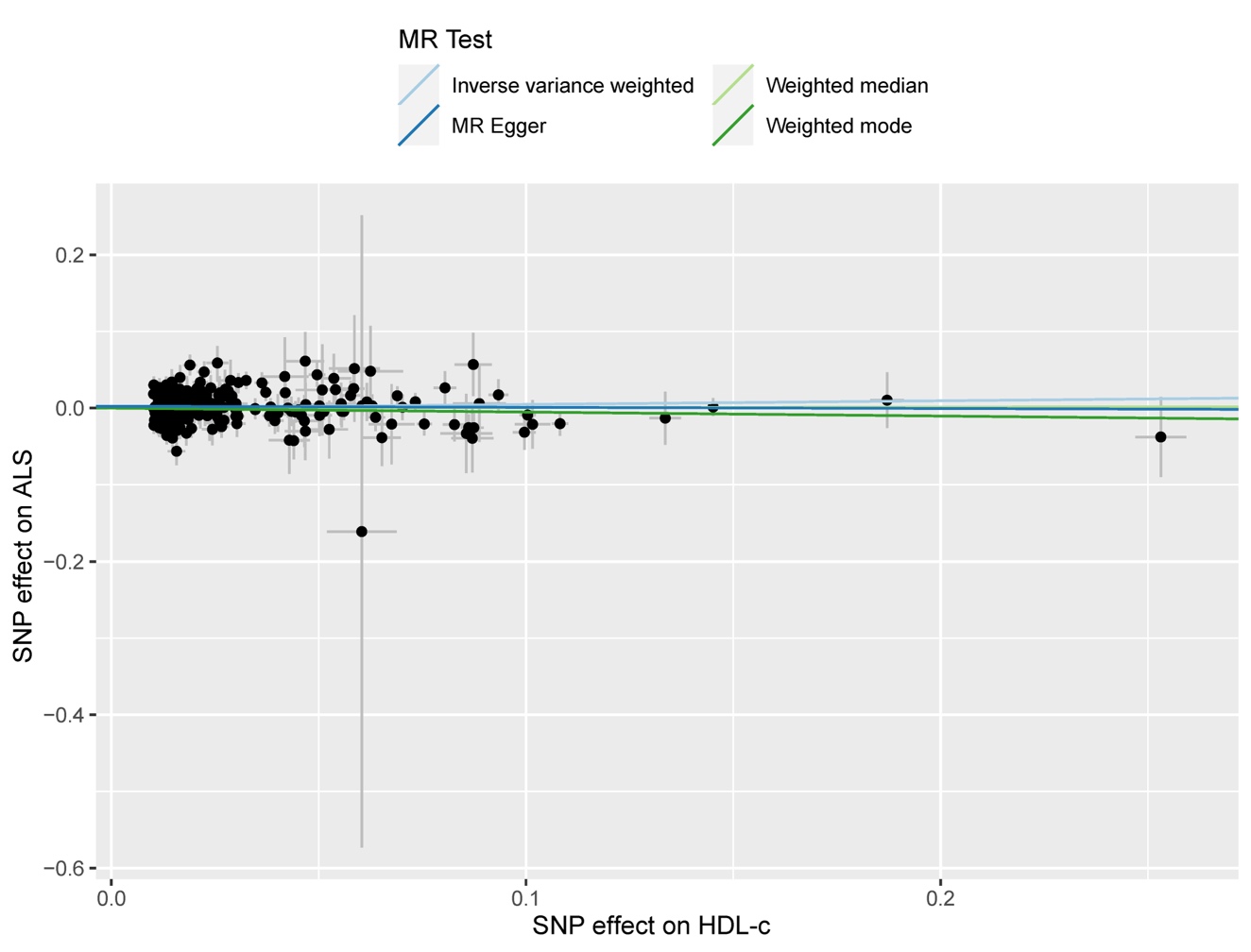


**Figure S10**. Scatter plot of individual SNP-ALS and SNP-HDL-c associations with an overlay of the causal estimate from each MR test in two-sample MR analysis.


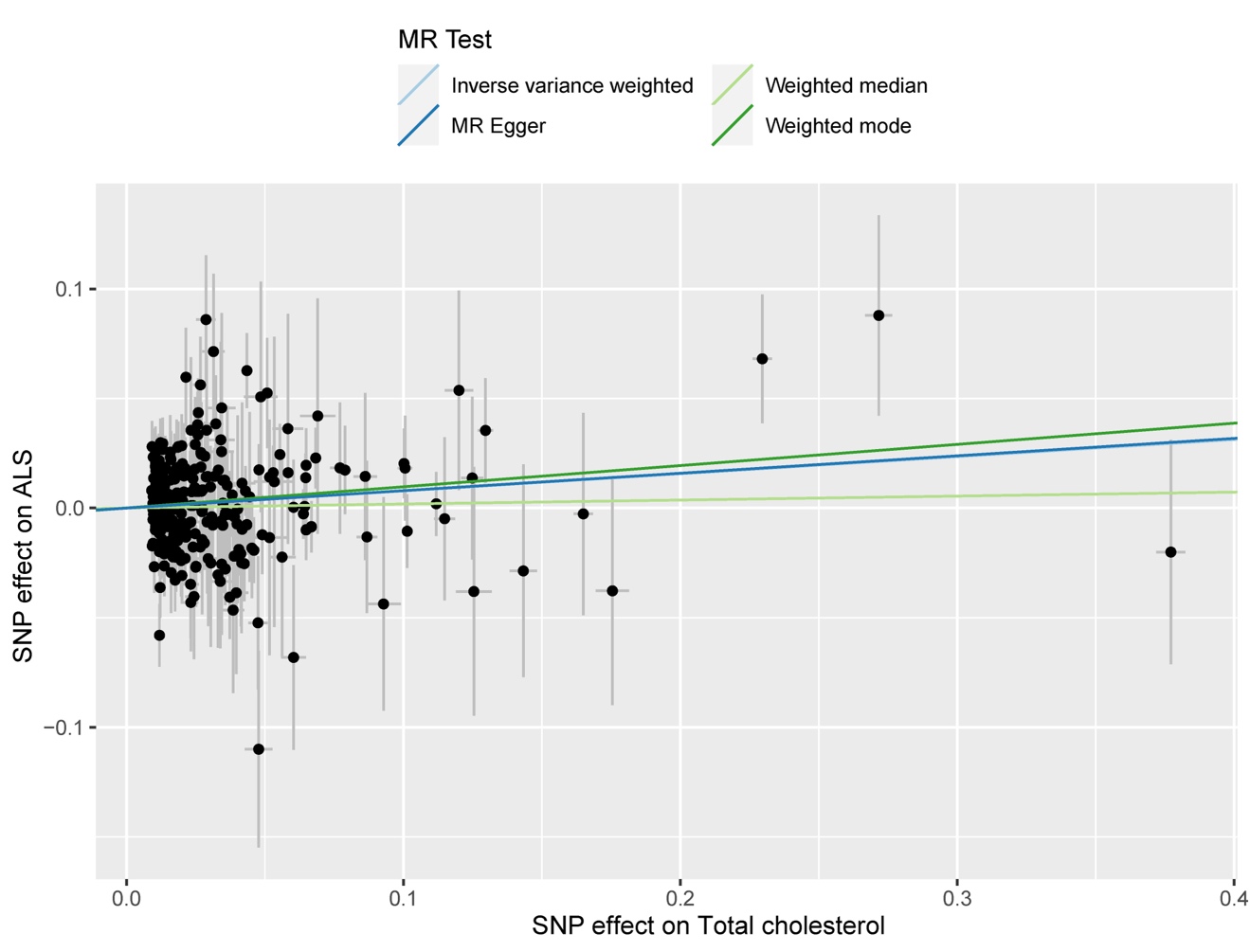


**Figure S11**. Scatter plot of individual SNP-ALS and SNP-total cholesterol associations with an overlay of the causal estimate from each MR test in two-sample MR analysis.


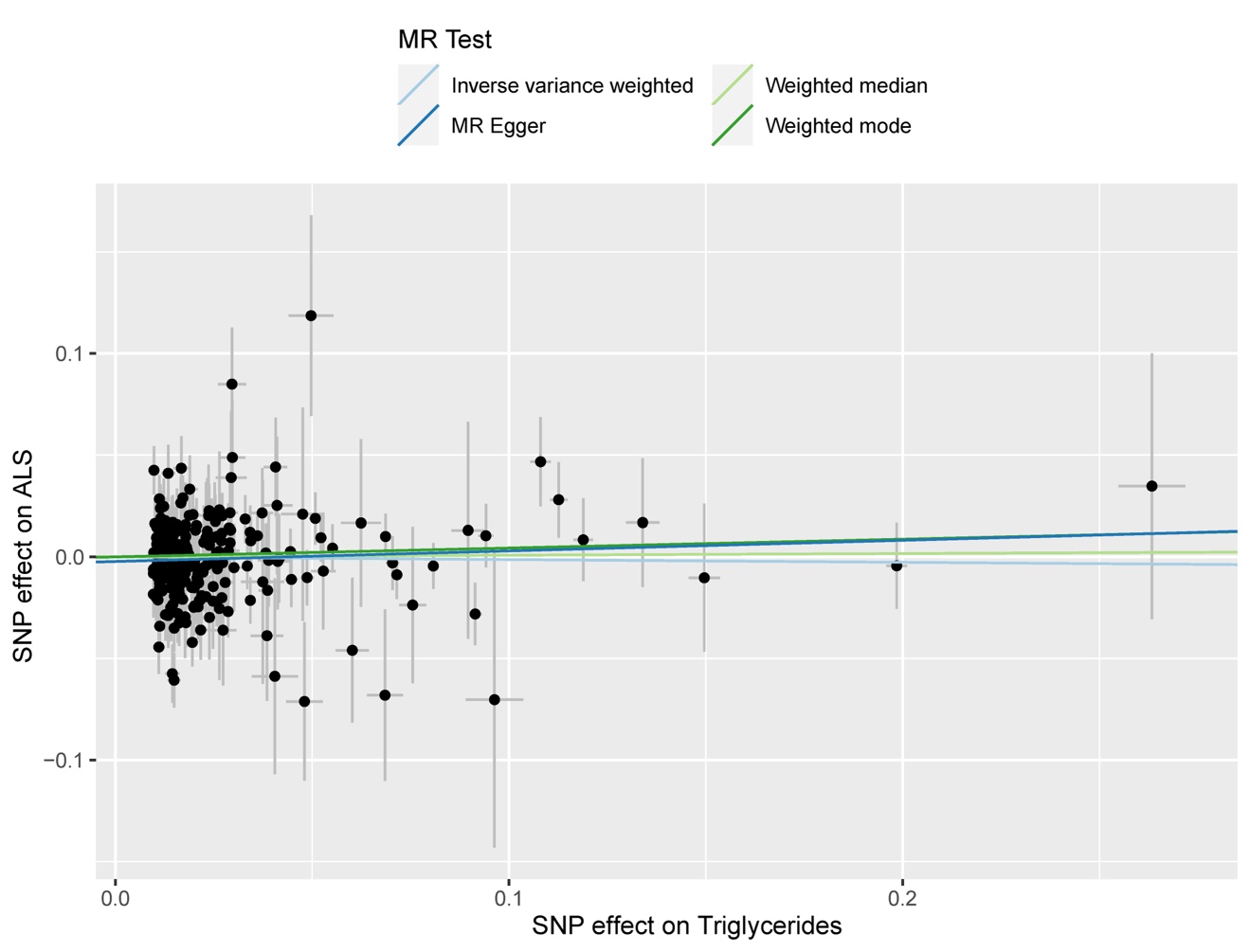


**Figure S12.** Scatter plot of individual SNP-ALS and SNP-triglycerides associations with an overlay of the causal estimate from each MR test in two-sample MR analysis.

**Table S3**. Heterogeneity and pleiotropy tests of instrument effects.

|  |  | **MR Egger regression** | | | **Heterogeneity test** | |
| --- | --- | --- | --- | --- | --- | --- |
| **Exposure** | **Outcome** | **Intercept** | **SE** | **P-value** | **Q-statistic** | **IVW - Q_P-value** |
| \| LDL-c \| \| --- \| | \| ALS \| \| --- \| | -0.0001 | 0.006 | 9.43E-01 | 411.6 | 2.61E-07 |
| \| HDL-c \| \| --- \| | \| ALS \| \| --- \| | 0.0025 | 0.0016 | 1.25E-01 | 369.9 | 5.87E-06 |
| \| Total cholesterol \| \| --- \| | \| ALS \| \| --- \| | 0.0000 | 0.0015 | 9.68E-01 | 401.1 | 1.36E-05 |
| \| Triglycerides \| \| --- \| | \| ALS \| \| --- \| | -0.0023 | 0.0016 | 1.55E-01 | 403.2 | 1.36E-07 |
| ApoA1 | \| ALS \| \| --- \| | 0.0164 | 0.0180 | 3.87E-01 | 14.6 | 1.02E-01 |
| ApoB | \| ALS \| \| --- \| | 0.0005 | 0.0070 | 9.48E-01 | 12.2 | 6.58E-01 |
| \| LDL-c \| \| --- \| | \| ALS survival \| \| --- \| | 0.0024 | 0.0036 | 5.01E-01 | 259 | 2.03E-01 |
| \| HDL-c \| \| --- \| | ALS survival | -0.0024 | 0.0039 | 5.39E-01 | 296.5 | 2.69E-01 |
| \| Total cholesterol \| \| --- \| | ALS survival | -0.0014 | 0.0035 | 6.84E-01 | 293.5 | 4.87E-02 |
| \| Triglycerides \| \| --- \| | ALS survival | -0.0025 | 0.0034 | 4.49E-01 | 278.5 | 5.86E-02 |
| ApoA1 | ALS survival | 0.0130 | 0.0369 | 7.35E-01 | 8.8 | 3.59E-01 |
| ApoB | ALS survival | -0.0128 | 0.0180 | 4.90E-01 | 13.9 | 4.57E-01 |
| \| LDL-c \| \| --- \| | FTD | 0.0122 | 0.0061 | 4.78E-02 | 198.7 | 5.52E-01 |
| \| HDL-c \| \| --- \| | FTD | -0.0009 | 0.0065 | 8.87E-01 | 218.6 | 1.18E-01 |
| \| Total cholesterol \| \| --- \| | FTD | 0.0018 | 0.0061 | 7.63E-01 | 194 | 8.19E-01 |
| \| Triglycerides \| \| --- \| | FTD | -0.0003 | 0.0056 | 9.55E-01 | 213.6 | 4.75E-01 |
| ApoA1 | FTD | 0.0147 | 0.0533 | 7.92E-01 | 3.1 | 8.76E-01 |
| ApoB | FTD | -0.0659 | 0.0332 | 1.04E-01 | 5.3 | 5.05E-01 |
| HMGCR (statins genetic proxy) | \| ALS \| \| --- \| | -0.0092 | 0.0232 | 6.94E-01 | 25.4 | 1.47E-01 |
| PCSK9 (alirocumab or evolocumab genetic proxy) | \| ALS \| \| --- \| | 0.0072 | 0.0044 | 1.08E-01 | 33.8 | 8.12E-01 |
| NPC1L1 (ezetimibe genetic proxy) | \| ALS \| \| --- \| | -0.0105 | 0.0242 | 6.81E-01 | 4.7 | 6.91E-01 |
| APOB (Mipomersen genetic proxy) | \| ALS \| \| --- \| | -0.0088 | 0.0077 | 2.65E-01 | 32.5 | 5.42E-01 |
| HMGCR (statins genetic proxy) | \| ALS survival \| \| --- \| | -0.0147 | 0.0459 | 7.53E-01 | 8.5 | 9.02E-01 |
| PCSK9 (alirocumab or evolocumab genetic proxy) | ALS survival | -0.0047 | 0.0189 | 8.05E-01 | 22.7 | 5.36E-01 |
| NPC1L1 (ezetimibe genetic proxy) | ALS survival | 0.0101 | 0.0521 | 8.56E-01 | 2.4 | 7.87E-01 |
| APOB (Mipomersen genetic proxy) | ALS survival | -0.0002 | 0.0185 | 9.92E-01 | 21.9 | 6.40E-01 |
| HMGCR (statins genetic proxy) | FTD | -0.0635 | 0.1884 | 7.58E-01 | 0.8 | 9.65E-01 |
| PCSK9 (alirocumab or evolocumab genetic proxy) | FTD | -0.0105 | 0.0295 | 7.26E-01 | 13.1 | 6.63E-01 |
| NPC1L1 (ezetimibe genetic proxy) | FTD | 0.0100 | 0.1246 | 9.49E-01 | 1.5 | 4.74E-01 |
| APOB (Mipomersen genetic proxy) | FTD | 0.0045 | 0.0332 | 8.94E-01 | 14.3 | 9.39E-01 |
| HMGCR (statins genetic proxy) | CAD | -0.0063 | 0.0142 | 6.60E-01 | 25.7 | 5.37E-01 |
| PCSK9 (alirocumab or evolocumab genetic proxy) | CAD | 0.0002 | 0.0040 | 9.64E-01 | 36.1 | 7.62E-01 |
| NPC1L1 (ezetimibe genetic proxy) | CAD | 0.0214 | 0.0194 | 3.05E-01 | 3.8 | 8.78E-01 |
| APOB (Mipomersen genetic proxy) | CAD | -0.0120 | 0.0089 | 1.90E-01 | 61.3 | 2.57E-03 |


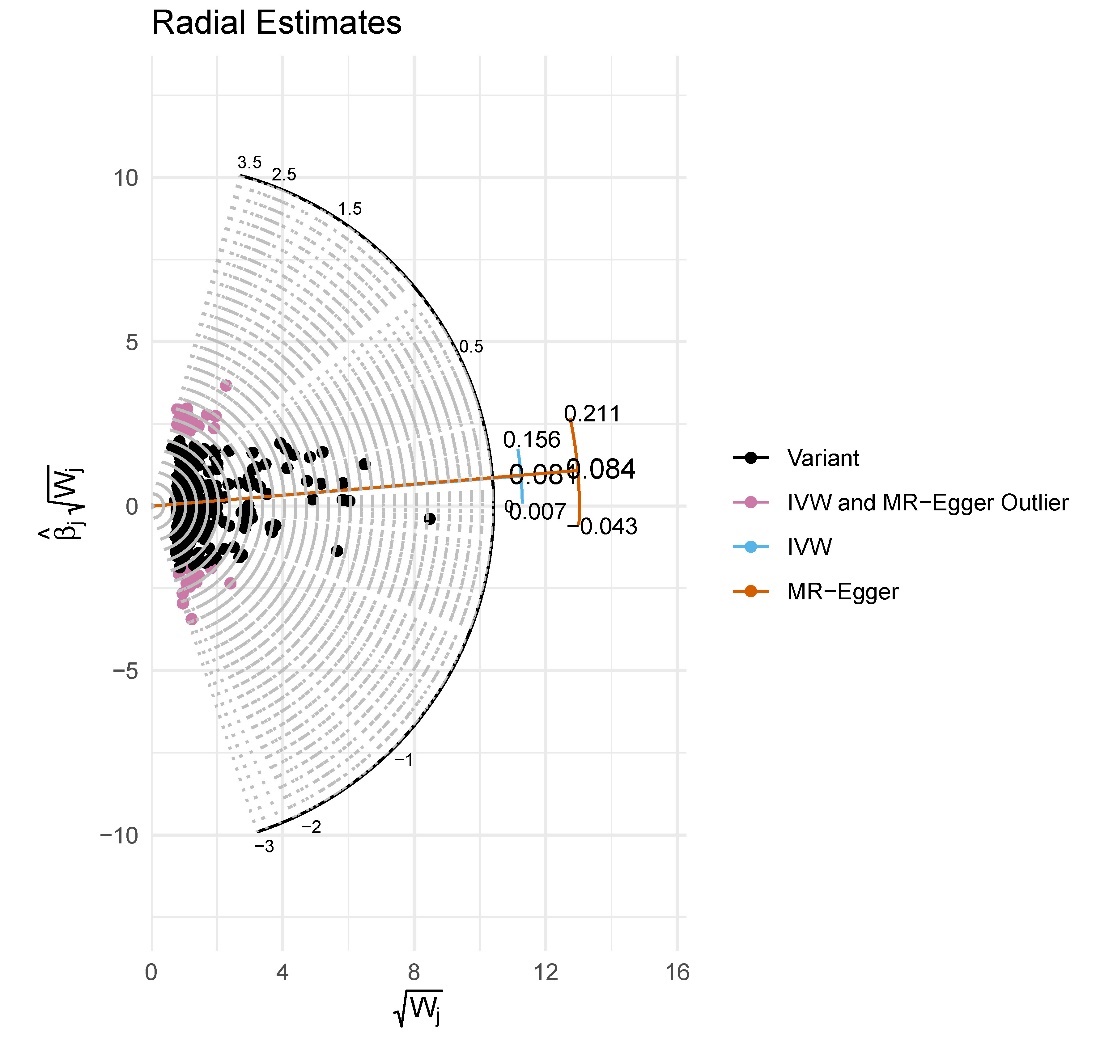


**Figure S13**. Radial curve displays the ratio estimate for each genetic variant, as well as the overall IVW (in blue) and MR Egger estimate (in orange) between LDL-c and ALS. Data points with large contributions to Cochran’s Q statistic are shown in pink, with an alpha of 0.05/nSNPs.


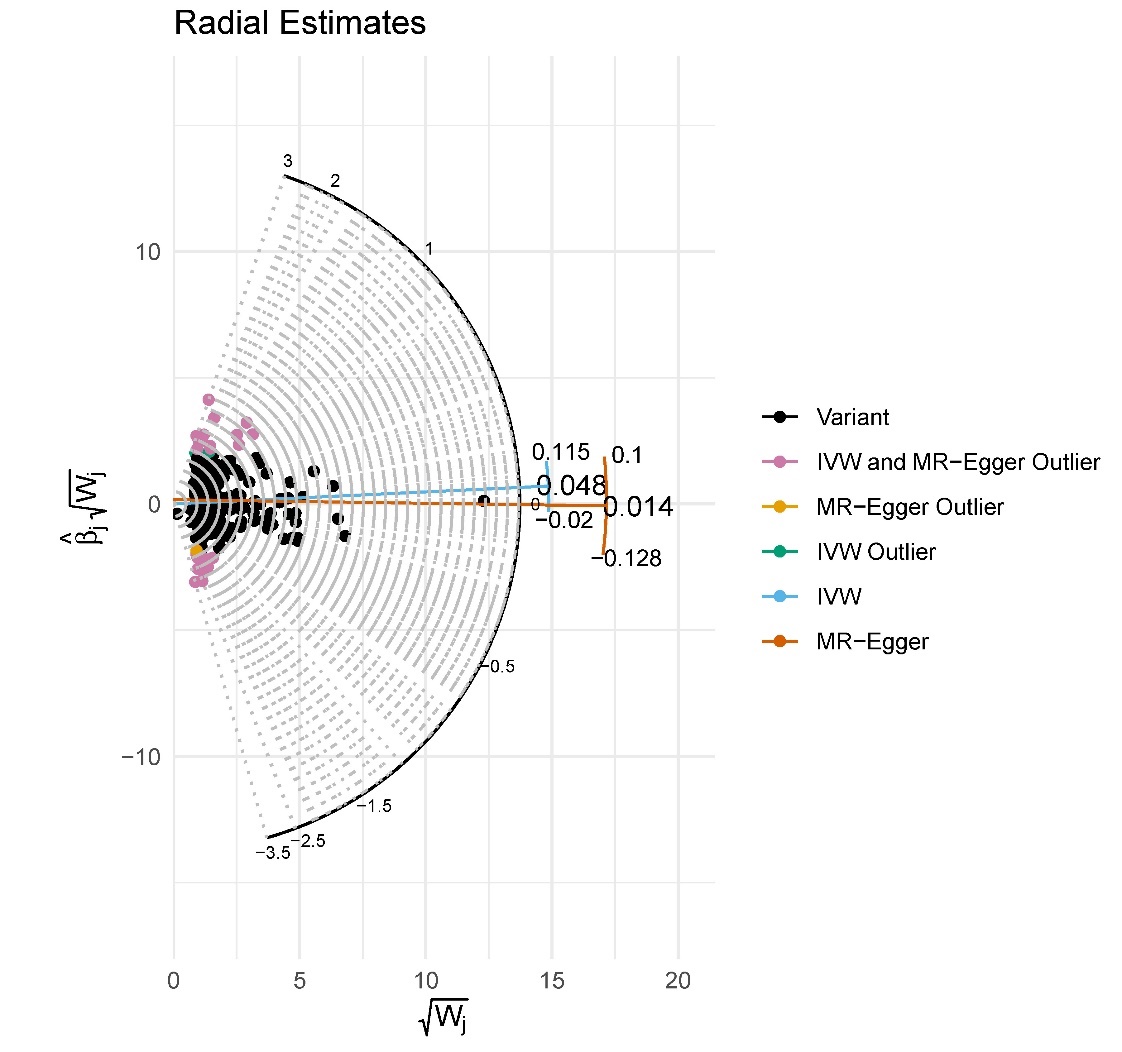


**Figure S14**. Radial curve displays the ratio estimate for each genetic variant, as well as the overall IVW (in blue) and MR Egger estimate (in orange) between HDL-c and ALS. Data points with large contributions to Cochran’s Q statistic are shown in pink, with an alpha of 0.05/nSNPs.


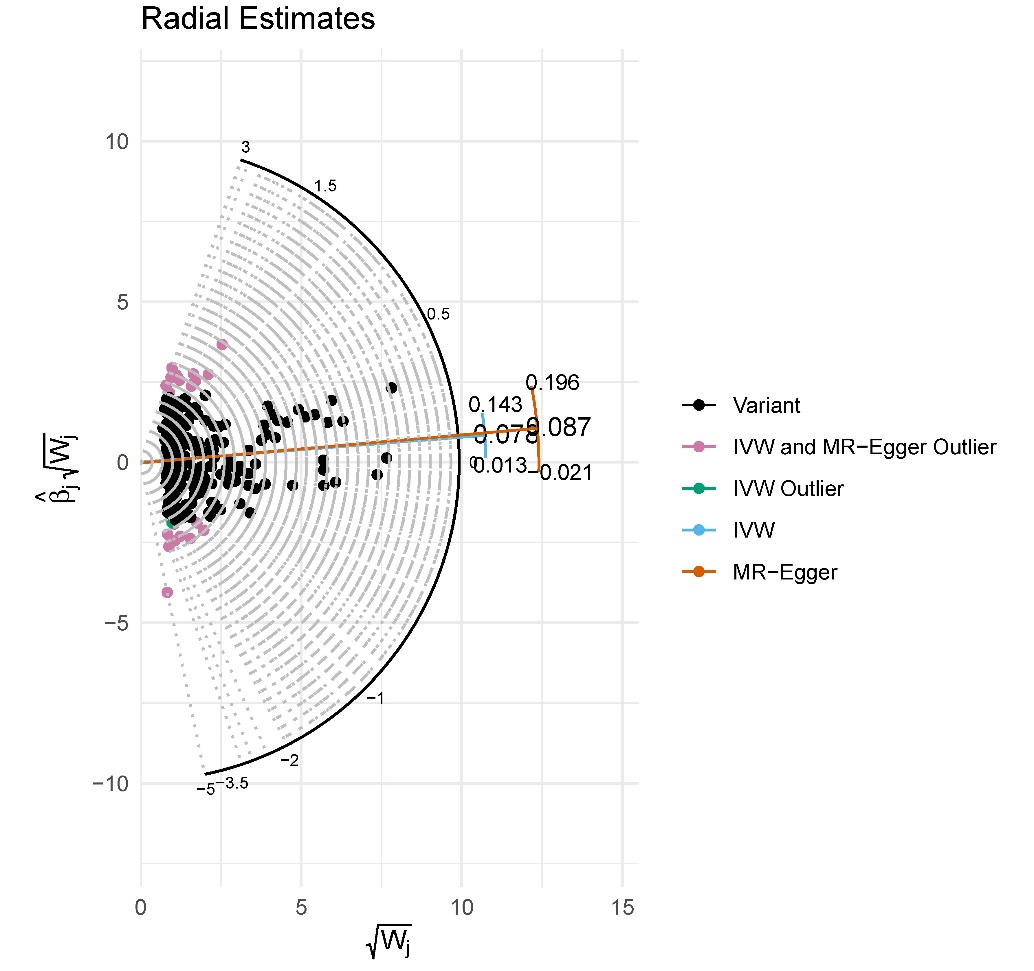


**Figure S15**. Radial curve displays the ratio estimate for each genetic variant, as well as the overall IVW (in blue) and MR Egger estimate (in orange) between total cholesterol and ALS. Data points with large contributions to Cochran’s Q statistic are shown in pink, with an alpha of 0.05/nSNPs.


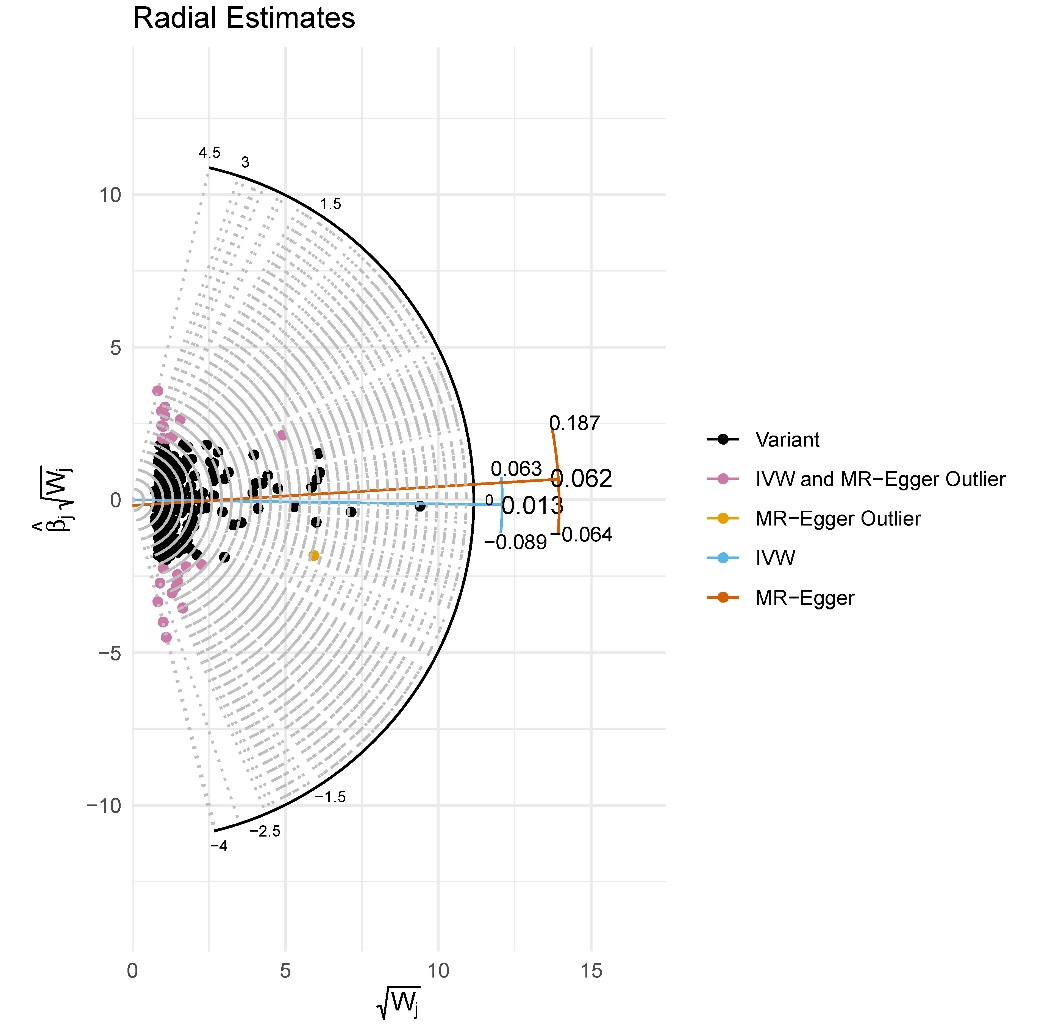


**Figure S16**. Radial curve displays the ratio estimate for each genetic variant, as well as the overall IVW (in blue) and MR Egger estimate (in orange) between triglycerides and ALS. Data points with large contributions to Cochran’s Q statistic are shown in pink, with an alpha of 0.05/nSNPs.

**Table S4**. Identified outliers with the most weight in the MR analysis and the largest contribution to Cochran’s Q statistic for heterogeneity from the MR Radial method.

|  |  | **MR-radial - IVW outlier** | | | **MR-radial -Egger outlier** | | |
| --- | --- | --- | --- | --- | --- | --- | --- |
| **Exposure** | **Outcome** | **SNP** | **Q_statistic** | **p.value** | **SNP** | **Q_statistic** | **p.value** |
| LDL-c | ALS | rs11175540 | 4.095004 | 4.30E-02 | rs11175540 | 4.083102 | 4.33E-02 |
| LDL-c | ALS | rs114863007 | 5.015886 | 2.51E-02 | rs114863007 | 5.005172 | 2.53E-02 |
| LDL-c | ALS | rs11621792 | 4.1639 | 4.13E-02 | rs11621792 | 4.158239 | 4.14E-02 |
| LDL-c | ALS | rs11837065 | 5.17702 | 2.29E-02 | rs11837065 | 5.192738 | 2.27E-02 |
| LDL-c | ALS | rs12055389 | 7.16158 | 7.45E-03 | rs12055389 | 7.178225 | 7.38E-03 |
| LDL-c | ALS | rs12094989 | 9.249471 | 2.36E-03 | rs12094989 | 9.228574 | 2.38E-03 |
| LDL-c | ALS | rs12138866 | 8.227657 | 4.13E-03 | rs12138866 | 8.249399 | 4.08E-03 |
| LDL-c | ALS | rs12306780 | 5.348186 | 2.07E-02 | rs12306780 | 5.363832 | 2.06E-02 |
| LDL-c | ALS | rs12451056 | 6.623139 | 1.01E-02 | rs12451056 | 6.642453 | 9.96E-03 |
| LDL-c | ALS | rs12603290 | 6.491157 | 1.08E-02 | rs12603290 | 6.491374 | 1.08E-02 |
| LDL-c | ALS | rs13108218 | 4.996752 | 2.54E-02 | rs13108218 | 4.98649 | 2.55E-02 |
| LDL-c | ALS | rs13301660 | 4.82859 | 2.80E-02 | rs13301660 | 4.841471 | 2.78E-02 |
| LDL-c | ALS | rs148086620 | 8.284438 | 4.00E-03 | rs148086620 | 8.302509 | 3.96E-03 |
| LDL-c | ALS | rs1515565 | 4.585787 | 3.22E-02 | rs1515565 | 4.569827 | 3.25E-02 |
| LDL-c | ALS | rs1571791 | 6.371511 | 1.16E-02 | rs1571791 | 6.385562 | 1.15E-02 |
| LDL-c | ALS | rs17532371 | 6.164509 | 1.30E-02 | rs17532371 | 6.182606 | 1.29E-02 |
| LDL-c | ALS | rs1992172 | 5.864991 | 1.54E-02 | rs1992172 | 5.853112 | 1.55E-02 |
| LDL-c | ALS | rs2250751 | 5.371565 | 2.05E-02 | rs2250751 | 5.382231 | 2.03E-02 |
| LDL-c | ALS | rs2296288 | 6.892015 | 8.66E-03 | rs2296288 | 6.909265 | 8.58E-03 |
| LDL-c | ALS | rs2374569 | 8.26665 | 4.04E-03 | rs2374569 | 8.288833 | 3.99E-03 |
| LDL-c | ALS | rs267733 | 12.539384 | 3.98E-04 | rs267733 | 12.519586 | 4.03E-04 |
| LDL-c | ALS | rs2792751 | 6.647642 | 9.93E-03 | rs2792751 | 6.653063 | 9.90E-03 |
| LDL-c | ALS | rs28929474 | 6.480292 | 1.09E-02 | rs28929474 | 6.465378 | 1.10E-02 |
| LDL-c | ALS | rs4390169 | 5.949796 | 1.47E-02 | rs4390169 | 5.934371 | 1.48E-02 |
| LDL-c | ALS | rs4671050 | 4.890901 | 2.70E-02 | rs4671050 | 4.895998 | 2.69E-02 |
| LDL-c | ALS | rs56118251 | 7.481973 | 6.23E-03 | rs56118251 | 7.463077 | 6.30E-03 |
| LDL-c | ALS | rs6431630 | 5.376693 | 2.04E-02 | rs6431630 | 5.364621 | 2.05E-02 |
| LDL-c | ALS | rs6689 | 12.117585 | 4.99E-04 | rs6689 | 12.119508 | 4.99E-04 |
| LDL-c | ALS | rs704 | 6.997594 | 8.16E-03 | rs704 | 7.006426 | 8.12E-03 |
| LDL-c | ALS | rs78242215 | 5.837195 | 1.57E-02 | rs78242215 | 5.8558 | 1.55E-02 |
| HDL-c | ALS | rs1051613 | 9.645456 | 1.90E-03 | rs1051613 | 10.247133 | 1.37E-03 |
| HDL-c | ALS | rs1054787 | 4.265086 | 3.89E-02 | rs1054787 | 4.676592 | 3.06E-02 |
| HDL-c | ALS | rs1107850 | 7.225921 | 7.19E-03 | rs1107850 | 6.741726 | 9.42E-03 |
| HDL-c | ALS | rs11694828 | 3.930221 | 4.74E-02 | rs12549603 | 5.203357 | 2.25E-02 |
| HDL-c | ALS | rs12549603 | 4.889899 | 2.70E-02 | rs12940636 | 5.182176 | 2.28E-02 |
| HDL-c | ALS | rs12940636 | 4.706548 | 3.00E-02 | rs137991784 | 10.543946 | 1.17E-03 |
| HDL-c | ALS | rs137991784 | 9.825799 | 1.72E-03 | rs1955512 | 4.597802 | 3.20E-02 |
| HDL-c | ALS | rs1955512 | 4.139282 | 4.19E-02 | rs2070512 | 4.931083 | 2.64E-02 |
| HDL-c | ALS | rs2070512 | 4.956703 | 2.60E-02 | rs2540949 | 4.199635 | 4.04E-02 |
| HDL-c | ALS | rs2307111 | 4.01136 | 4.52E-02 | rs267738 | 10.74009 | 1.05E-03 |
| HDL-c | ALS | rs2540949 | 4.516533 | 3.36E-02 | rs2792751 | 6.7972 | 9.13E-03 |
| HDL-c | ALS | rs267738 | 11.175655 | 8.29E-04 | rs2941657 | 4.142743 | 4.18E-02 |
| HDL-c | ALS | rs2792751 | 6.854004 | 8.84E-03 | rs3803800 | 15.888677 | 6.72E-05 |
| HDL-c | ALS | rs3803800 | 16.531829 | 4.78E-05 | rs3808460 | 9.598514 | 1.95E-03 |
| HDL-c | ALS | rs3808460 | 9.516748 | 2.04E-03 | rs4675812 | 8.097377 | 4.43E-03 |
| HDL-c | ALS | rs4675812 | 7.57975 | 5.90E-03 | rs6060086 | 7.423609 | 6.44E-03 |
| HDL-c | ALS | rs4851250 | 3.916597 | 4.78E-02 | rs6710091 | 6.937796 | 8.44E-03 |
| HDL-c | ALS | rs6060086 | 6.856926 | 8.83E-03 | rs686030 | 6.924521 | 8.50E-03 |
| HDL-c | ALS | rs6710091 | 6.512256 | 1.07E-02 | rs6882591 | 5.272753 | 2.17E-02 |
| HDL-c | ALS | rs686030 | 6.778338 | 9.23E-03 | rs73243877 | 4.610197 | 3.18E-02 |
| HDL-c | ALS | rs6882591 | 5.75724 | 1.64E-02 | rs746390 | 4.105634 | 4.27E-02 |
| HDL-c | ALS | rs73243877 | 4.941772 | 2.62E-02 | rs7586605 | 5.35162 | 2.07E-02 |
| HDL-c | ALS | rs746390 | 4.553481 | 3.29E-02 | rs7684939 | 6.468939 | 1.10E-02 |
| HDL-c | ALS | rs7586605 | 4.875951 | 2.72E-02 | rs7732603 | 3.961801 | 4.65E-02 |
| HDL-c | ALS | rs7684939 | 7.037264 | 7.98E-03 | rs78424108 | 6.207527 | 1.27E-02 |
| HDL-c | ALS | rs78424108 | 6.690475 | 9.69E-03 | rs9374588 | 5.266269 | 2.17E-02 |
| HDL-c | ALS | rs9374588 | 4.843226 | 2.78E-02 |  |  |  |
| Total cholesterol | ALS | rs111299924 | 5.326141 | 2.10E-02 | rs111299924 | 5.369002 | 2.05E-02 |
| Total cholesterol | ALS | rs11175540 | 4.038235 | 4.45E-02 | rs11175540 | 3.979004 | 4.61E-02 |
| Total cholesterol | ALS | rs114863007 | 5.189684 | 2.27E-02 | rs114863007 | 5.161555 | 2.31E-02 |
| Total cholesterol | ALS | rs11621792 | 4.117615 | 4.24E-02 | rs11621792 | 4.084829 | 4.33E-02 |
| Total cholesterol | ALS | rs12138866 | 8.172209 | 4.25E-03 | rs12138866 | 8.260364 | 4.05E-03 |
| Total cholesterol | ALS | rs12306780 | 5.435756 | 1.97E-02 | rs12306780 | 5.516079 | 1.88E-02 |
| Total cholesterol | ALS | rs12451056 | 6.592077 | 1.02E-02 | rs12451056 | 6.67275 | 9.79E-03 |
| Total cholesterol | ALS | rs12575636 | 7.246123 | 7.11E-03 | rs12575636 | 7.15854 | 7.46E-03 |
| Total cholesterol | ALS | rs13108218 | 5.160652 | 2.31E-02 | rs13108218 | 5.133239 | 2.35E-02 |
| Total cholesterol | ALS | rs1567889 | 16.981909 | 3.77E-05 | rs1567889 | 16.844006 | 4.06E-05 |
| Total cholesterol | ALS | rs1571791 | 6.419767 | 1.13E-02 | rs1571791 | 6.488783 | 1.09E-02 |
| Total cholesterol | ALS | rs17532371 | 6.051123 | 1.39E-02 | rs17532371 | 6.116117 | 1.34E-02 |
| Total cholesterol | ALS | rs1992172 | 5.790397 | 1.61E-02 | rs1992172 | 5.728218 | 1.67E-02 |
| Total cholesterol | ALS | rs2296288 | 6.874318 | 8.74E-03 | rs2296288 | 6.948328 | 8.39E-03 |
| Total cholesterol | ALS | rs2792751 | 6.619583 | 1.01E-02 | rs2792751 | 6.643686 | 9.95E-03 |
| Total cholesterol | ALS | rs28929474 | 6.415239 | 1.13E-02 | rs28929474 | 6.341539 | 1.18E-02 |
| Total cholesterol | ALS | rs3732359 | 6.254624 | 1.24E-02 | rs3732359 | 6.204823 | 1.27E-02 |
| Total cholesterol | ALS | rs4671050 | 5.036263 | 2.48E-02 | rs4671050 | 5.079895 | 2.42E-02 |
| Total cholesterol | ALS | rs57854543 | 3.875728 | 4.90E-02 | rs58588820 | 4.482937 | 3.42E-02 |
| Total cholesterol | ALS | rs58588820 | 4.414689 | 3.56E-02 | rs5998509 | 5.799573 | 1.60E-02 |
| Total cholesterol | ALS | rs5998509 | 5.758873 | 1.64E-02 | rs6689 | 12.033378 | 5.23E-04 |
| Total cholesterol | ALS | rs6689 | 12.029054 | 5.24E-04 | rs686030 | 6.939151 | 8.43E-03 |
| Total cholesterol | ALS | rs686030 | 6.89113 | 8.66E-03 | rs9898605 | 5.256928 | 2.19E-02 |
| Total cholesterol | ALS | rs9898605 | 5.333644 | 2.09E-02 |  |  |  |
| Triglycerides | ALS | rs11113118 | 4.325898 | 3.75E-02 | rs11113118 | 4.688871 | 3.04E-02 |
| Triglycerides | ALS | rs111998037 | 11.070554 | 8.77E-04 | rs111998037 | 10.281632 | 1.34E-03 |
| Triglycerides | ALS | rs115276619 | 5.828667 | 1.58E-02 | rs115276619 | 6.359499 | 1.17E-02 |
| Triglycerides | ALS | rs11651783 | 20.084096 | 7.41E-06 | rs11651783 | 19.19637 | 1.18E-05 |
| Triglycerides | ALS | rs12478327 | 7.012513 | 8.09E-03 | rs12478327 | 7.366743 | 6.64E-03 |
| Triglycerides | ALS | rs12953014 | 8.019517 | 4.63E-03 | rs12953014 | 7.592694 | 5.86E-03 |
| Triglycerides | ALS | rs13108218 | 4.359602 | 3.68E-02 | rs13108218 | 4.302022 | 3.81E-02 |
| Triglycerides | ALS | rs1377587 | 12.833885 | 3.40E-04 | rs1377587 | 13.717296 | 2.12E-04 |
| Triglycerides | ALS | rs17713879 | 5.954154 | 1.47E-02 | rs17713879 | 6.510313 | 1.07E-02 |
| Triglycerides | ALS | rs2009170 | 4.976016 | 2.57E-02 | rs2009170 | 4.506422 | 3.38E-02 |
| Triglycerides | ALS | rs2063724 | 15.837393 | 6.90E-05 | rs2063724 | 14.99213 | 1.08E-04 |
| Triglycerides | ALS | rs2523608 | 9.210127 | 2.41E-03 | rs2523608 | 8.697664 | 3.19E-03 |
| Triglycerides | ALS | rs2540949 | 4.713976 | 2.99E-02 | rs2540949 | 4.495032 | 3.40E-02 |
| Triglycerides | ALS | rs2803619 | 7.064418 | 7.86E-03 | rs2803619 | 6.686997 | 9.71E-03 |
| Triglycerides | ALS | rs296883 | 7.376357 | 6.61E-03 | rs296883 | 6.762869 | 9.31E-03 |
| Triglycerides | ALS | rs56078619 | 8.529432 | 3.49E-03 | rs56078619 | 9.194191 | 2.43E-03 |
| Triglycerides | ALS | rs58542926 | 4.788918 | 2.86E-02 | rs58542926 | 4.011818 | 4.52E-02 |
| Triglycerides | ALS | rs6448429 | 5.90137 | 1.51E-02 | rs6448429 | 5.554674 | 1.84E-02 |
| Triglycerides | ALS | rs686030 | 7.658119 | 5.65E-03 | rs686030 | 8.241905 | 4.09E-03 |
| Triglycerides | ALS | rs7212201 | 12.361295 | 4.38E-04 | rs7212201 | 11.944169 | 5.48E-04 |
| Triglycerides | ALS | rs73029263 | 4.061081 | 4.39E-02 | rs73029263 | 4.464257 | 3.46E-02 |
| Triglycerides | ALS | rs7312441 | 4.120469 | 4.24E-02 | rs7312441 | 4.580851 | 3.23E-02 |
| Triglycerides | ALS | rs74714416 | 9.346475 | 2.23E-03 | rs74714416 | 9.98945 | 1.57E-03 |
| Triglycerides | ALS |  |  |  | rs75627662 | 4.03075 | 4.47E-02 |


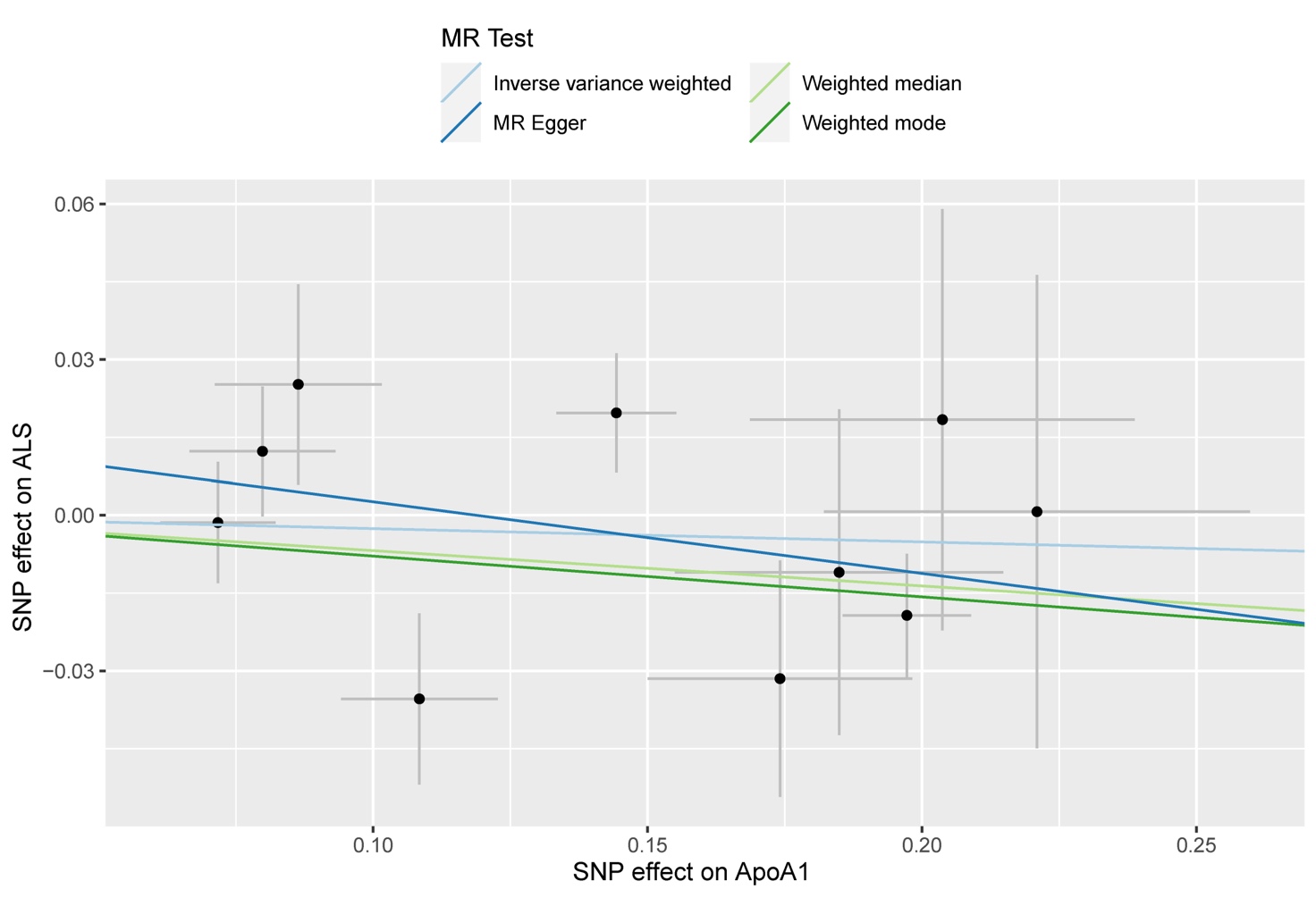


**Figure S17**. Scatter plot of individual SNP-ALS and SNP-ApoA1 associations with an overlay of the causal estimate from each MR test in two-sample MR analysis.


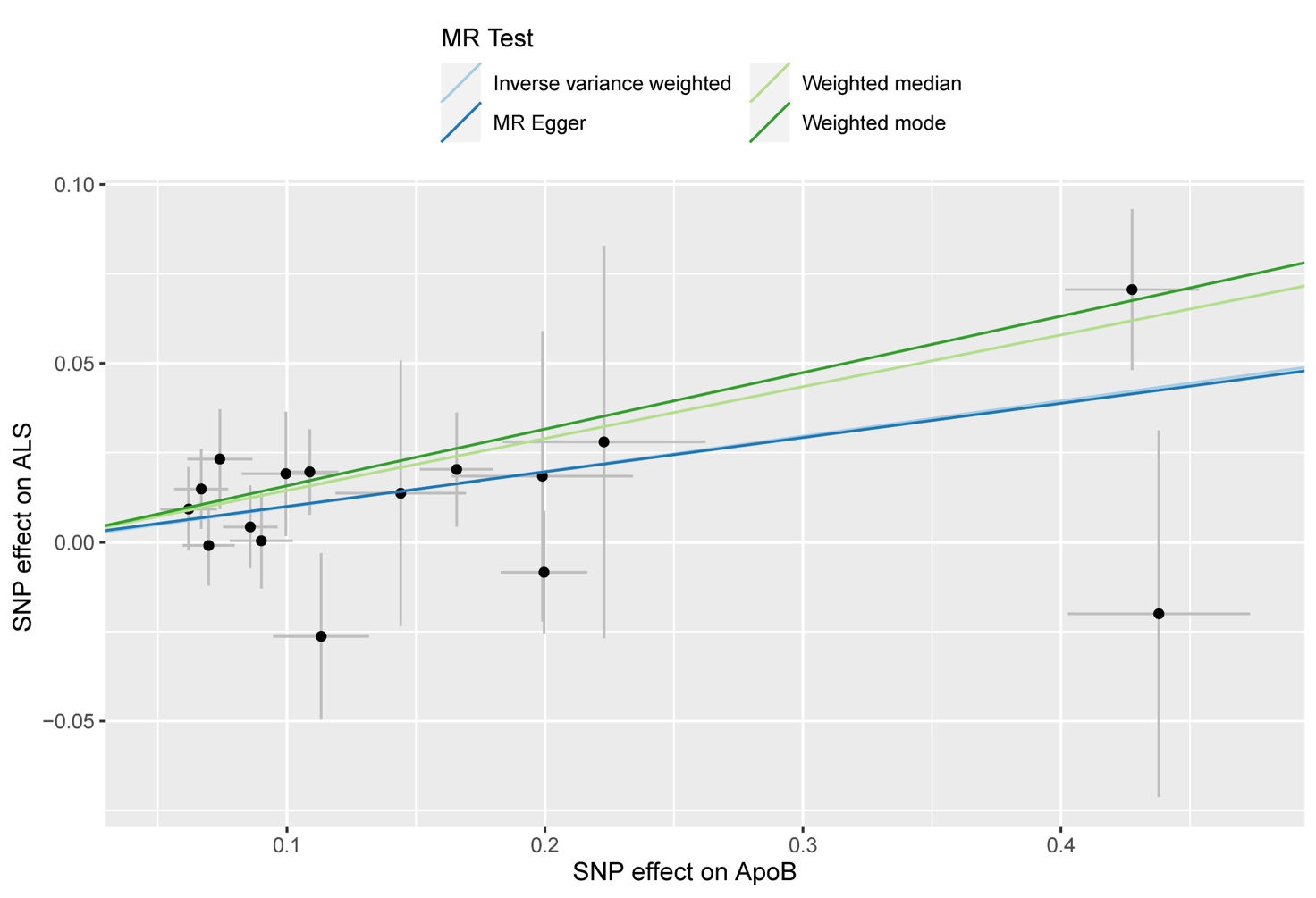


**Figure S18**. Scatter plot of individual SNP-ALS and SNP-ApoB associations with an overlay of the causal estimate from each MR test in two-sample MR analysis.


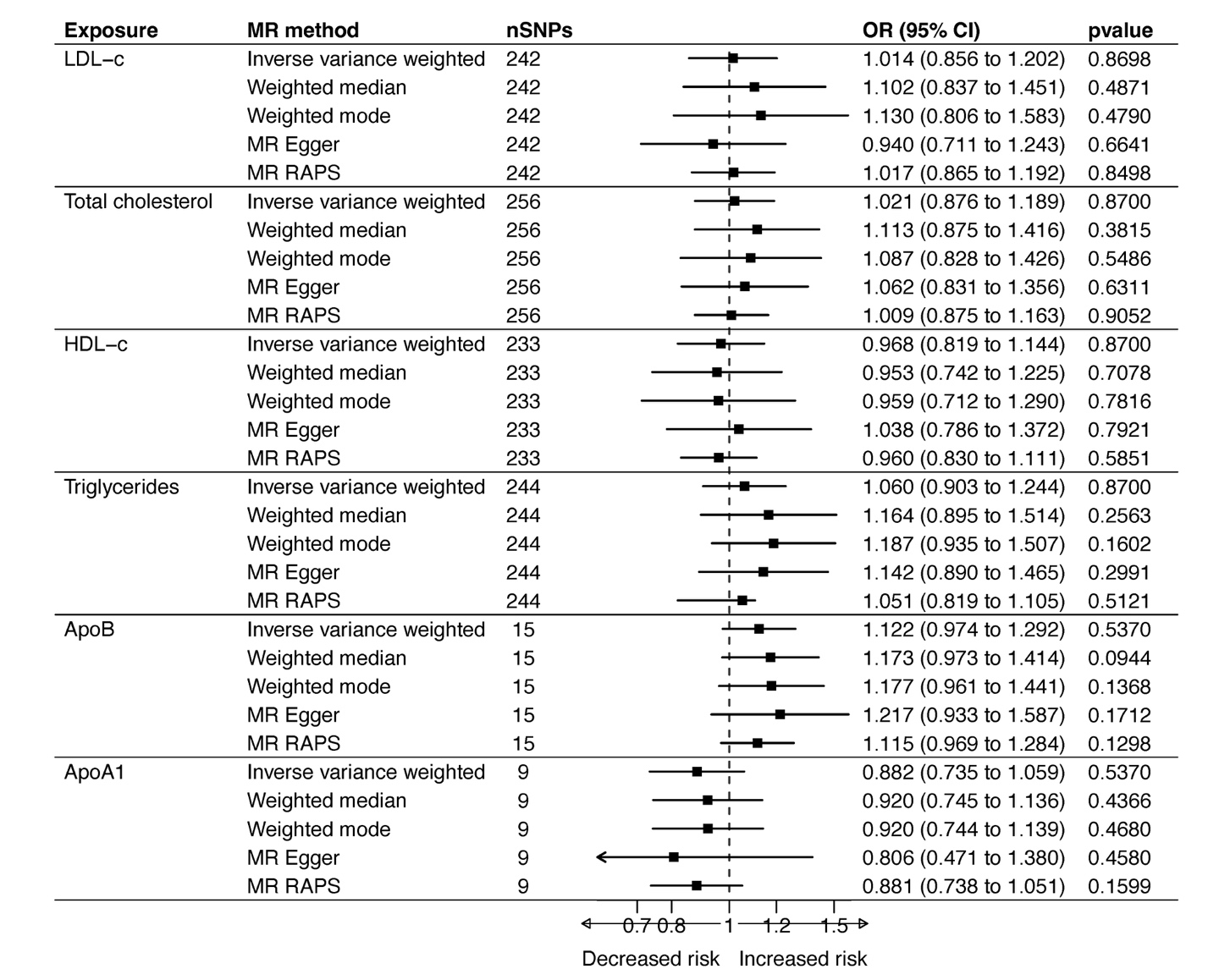


**Figure S19**. Univariate MR of the association of genetically predicted lipids and apolipoproteins levels with ALS survival. Forest plot of the association between a 1-SD change in the lipid and apolipoprotein levels with ALS survival. The p-value corresponding to the IVW method is an FDR-adjusted p-value. Abbreviations: LDL-c, low-density lipoprotein cholesterol; HDL-c, high-density lipoprotein cholesterol OR, odds ratio; SNP, single-nucleotide polymorphisms; ApoB, Apolipoprotein B; ApoA1, Apolipoprotein A1


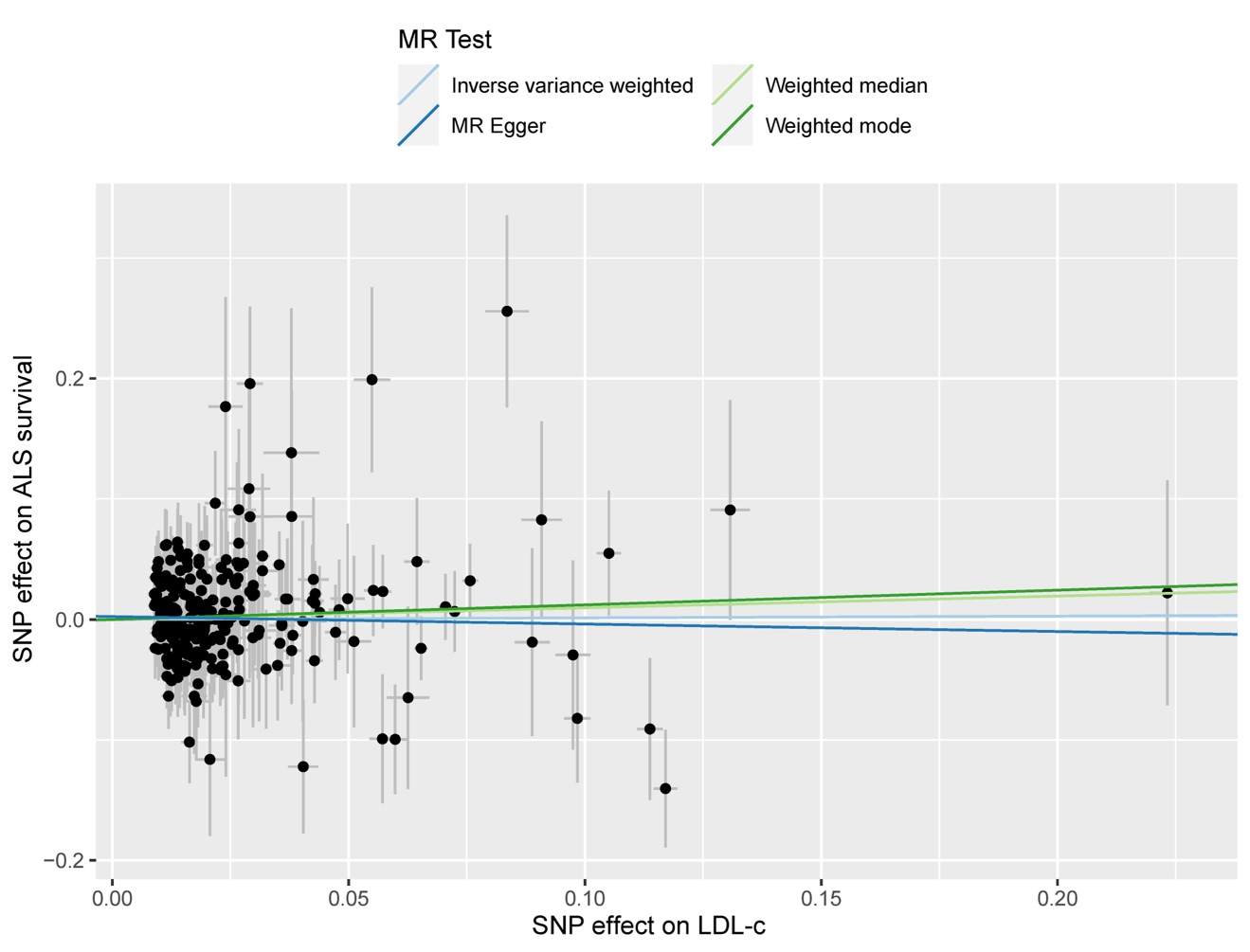


**Figure S20**. Scatter plot of individual SNP-ALS survival and SNP-LDL-c associations with an overlay of the causal estimate from each MR test in two-sample MR analysis


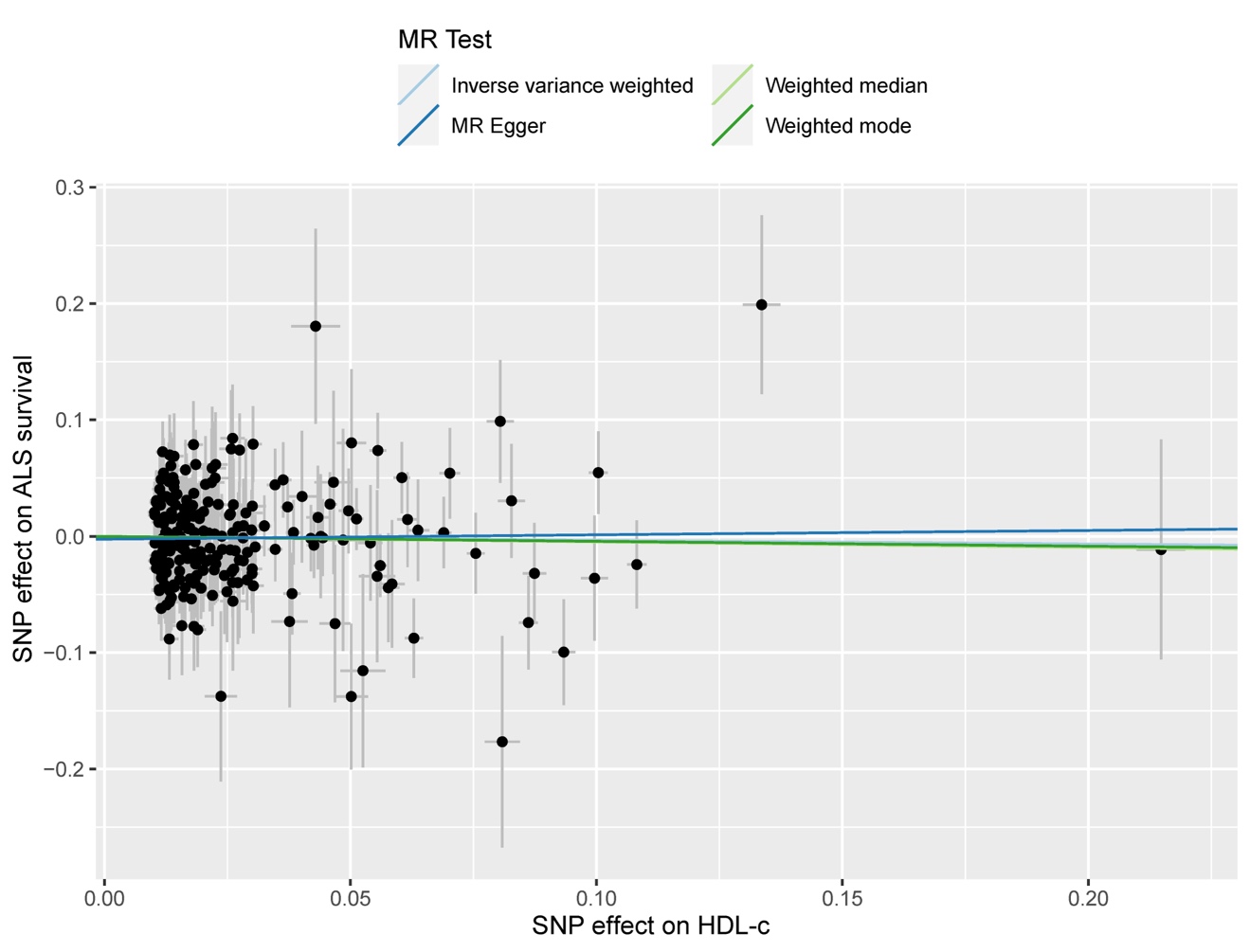


**Figure S21**. Scatter plot of individual SNP-ALS survival and SNP-HDL-c associations with an overlay of the causal estimate from each MR test in two-sample MR analysis.


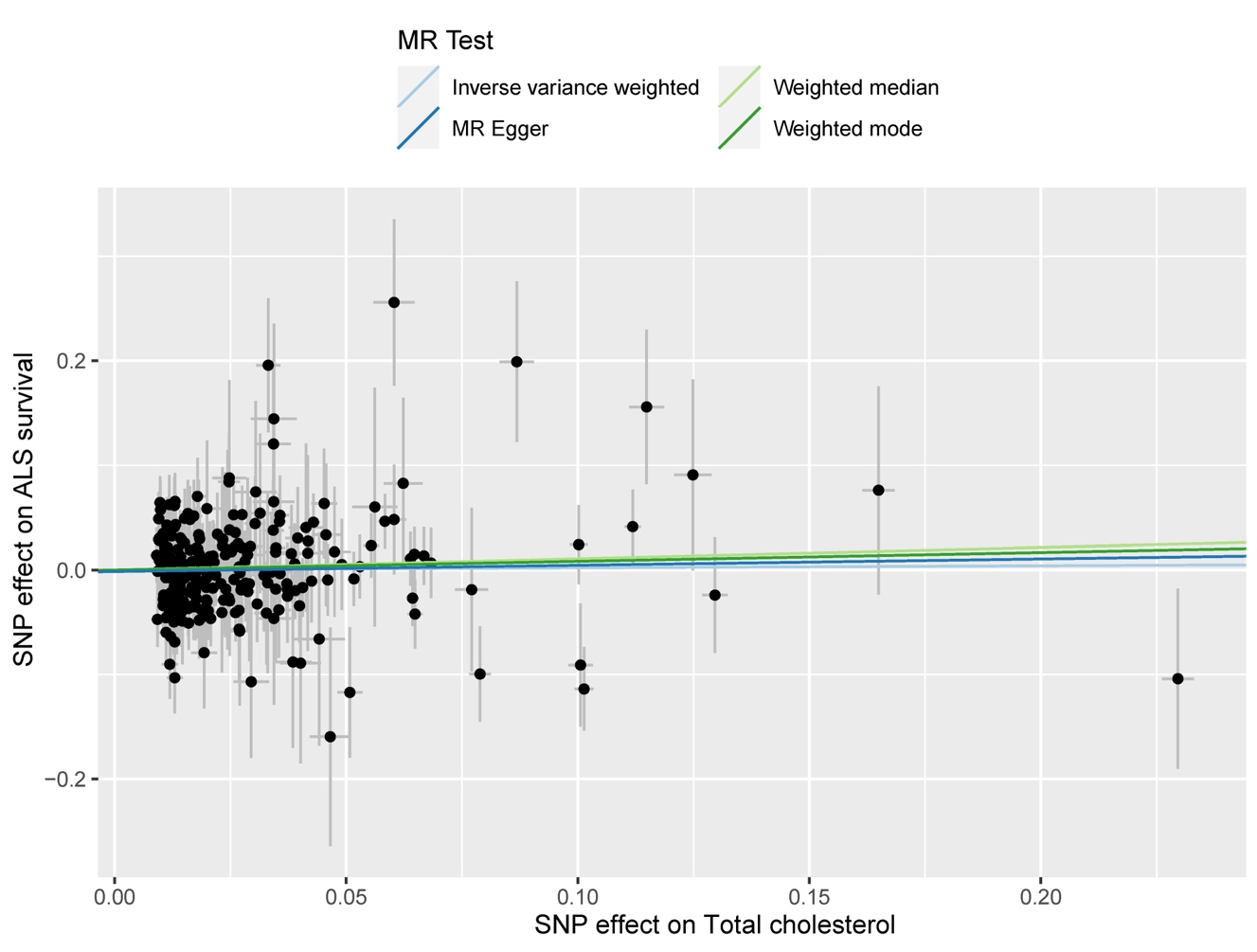


**Figure S22**. Scatter plot of individual SNP-ALS survival and SNP-total cholesterol associations with an overlay of the causal estimate from each MR test in two-sample MR analysis.


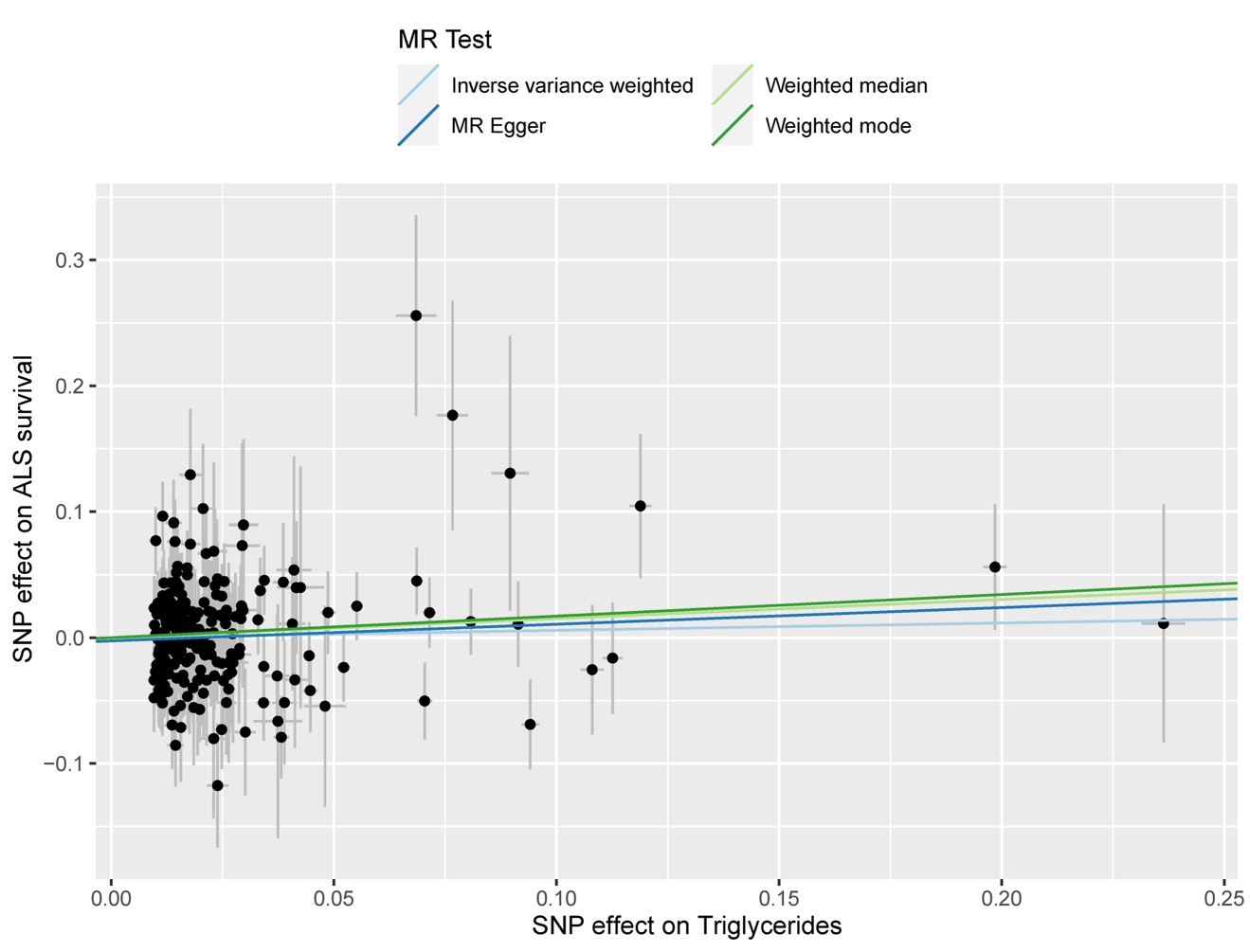


**Figure S23**. Scatter plot of individual SNP-ALS survival and SNP-triglycerides associations with an overlay of the causal estimate from each MR test in two-sample MR analysis.


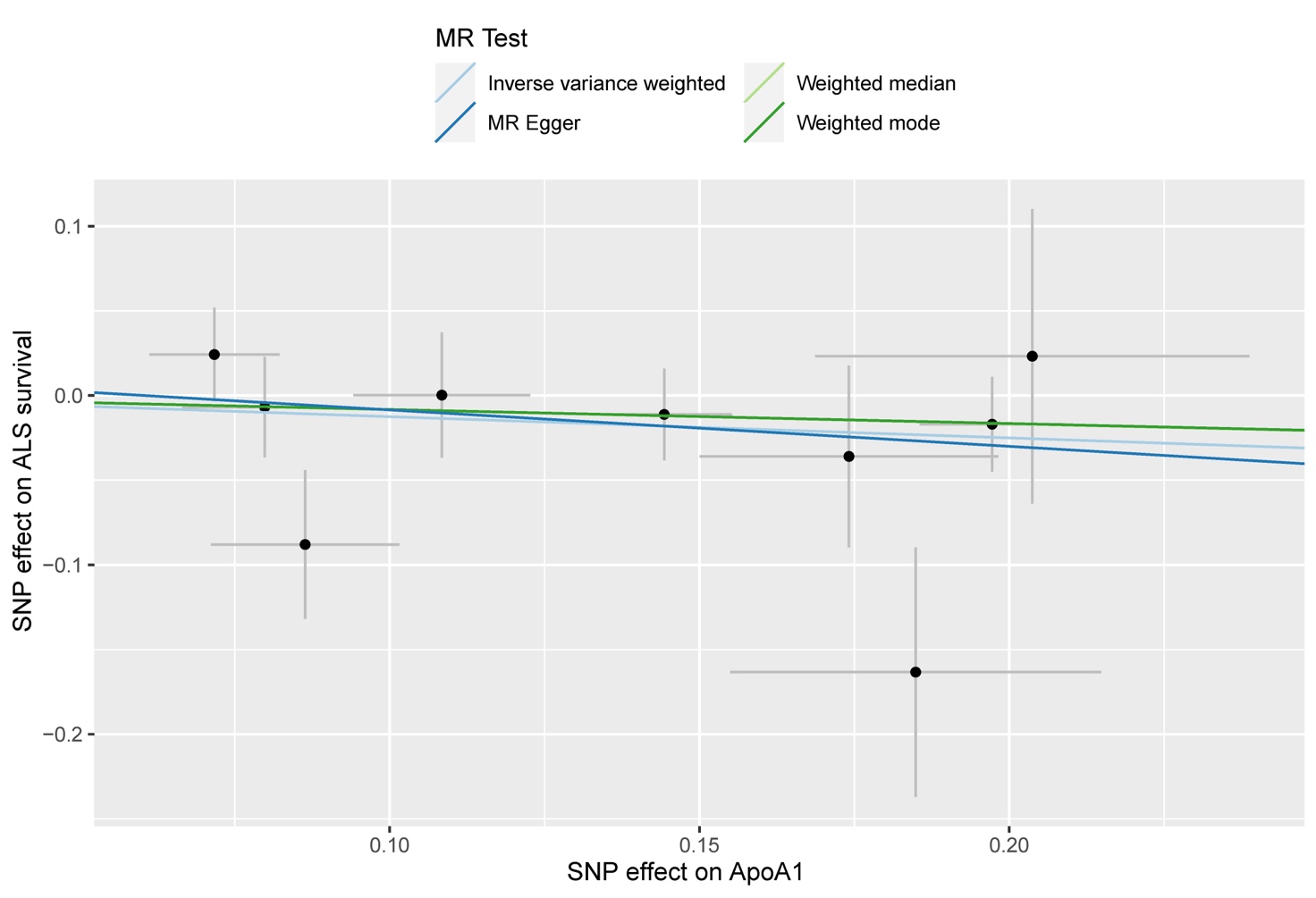


**Figure S24**. Scatter plot of individual SNP-ALS survival and SNP-ApoA1 associations with an overlay of the causal estimate from each MR test in two-sample MR analysis.


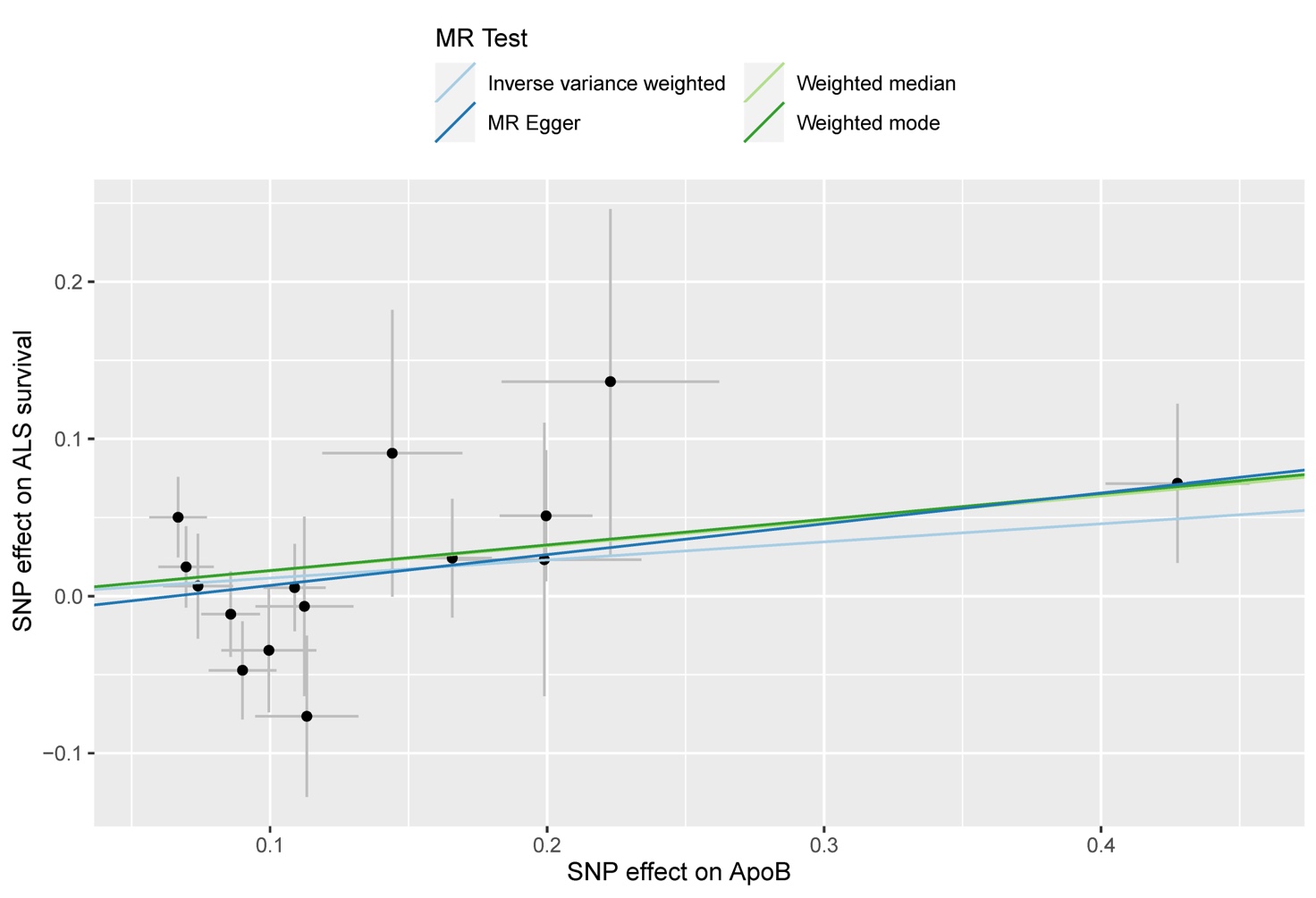


**Figure S25**. Scatter plot of individual SNP-ALS survival and SNP-ApoB associations with an overlay of the causal estimate from each MR test in two-sample MR analysis.


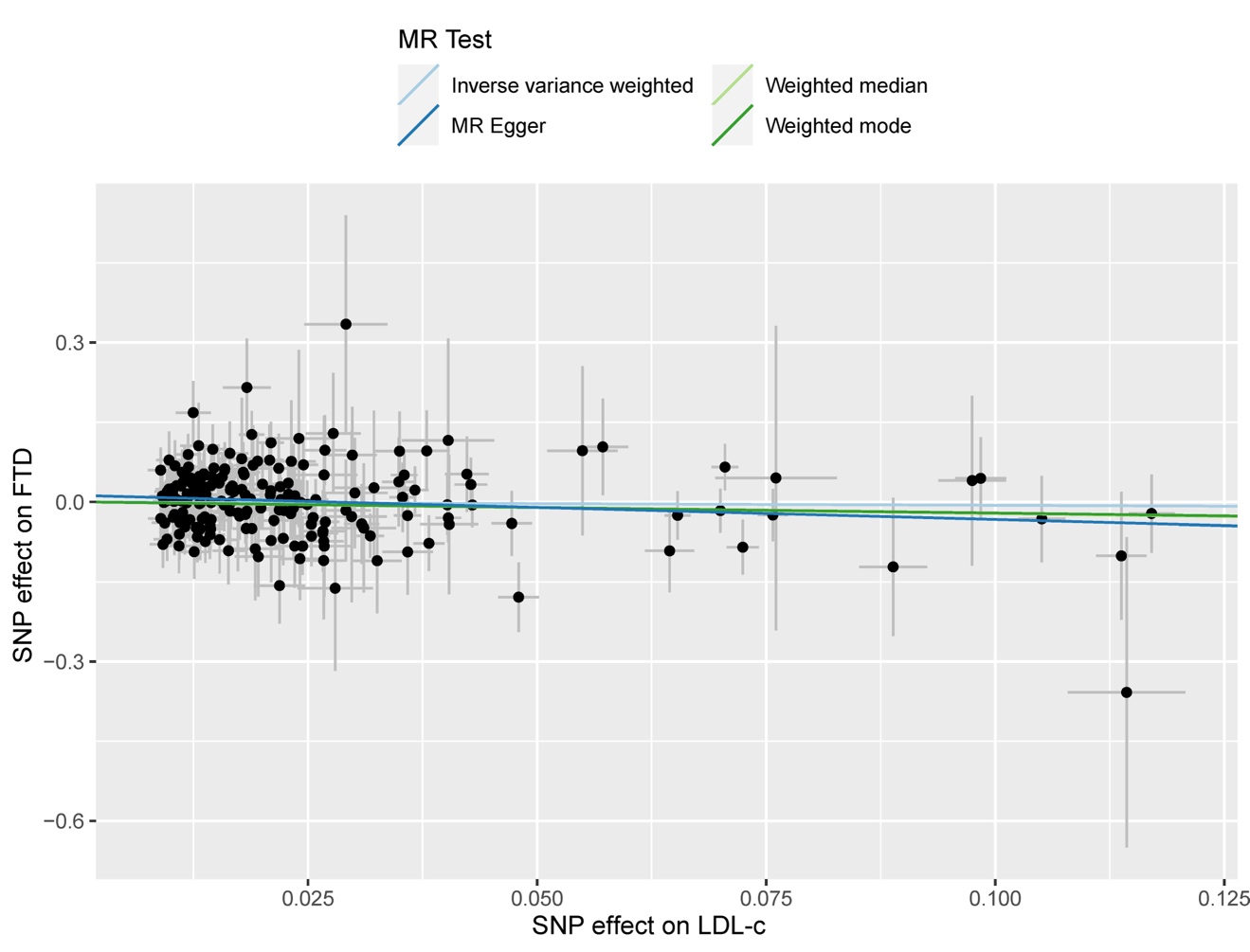


**Figure S26**. Scatter plot of individual SNP-FTD and SNP-LDL-c associations with an overlay of the causal estimate from each MR test in two-sample MR analysis.


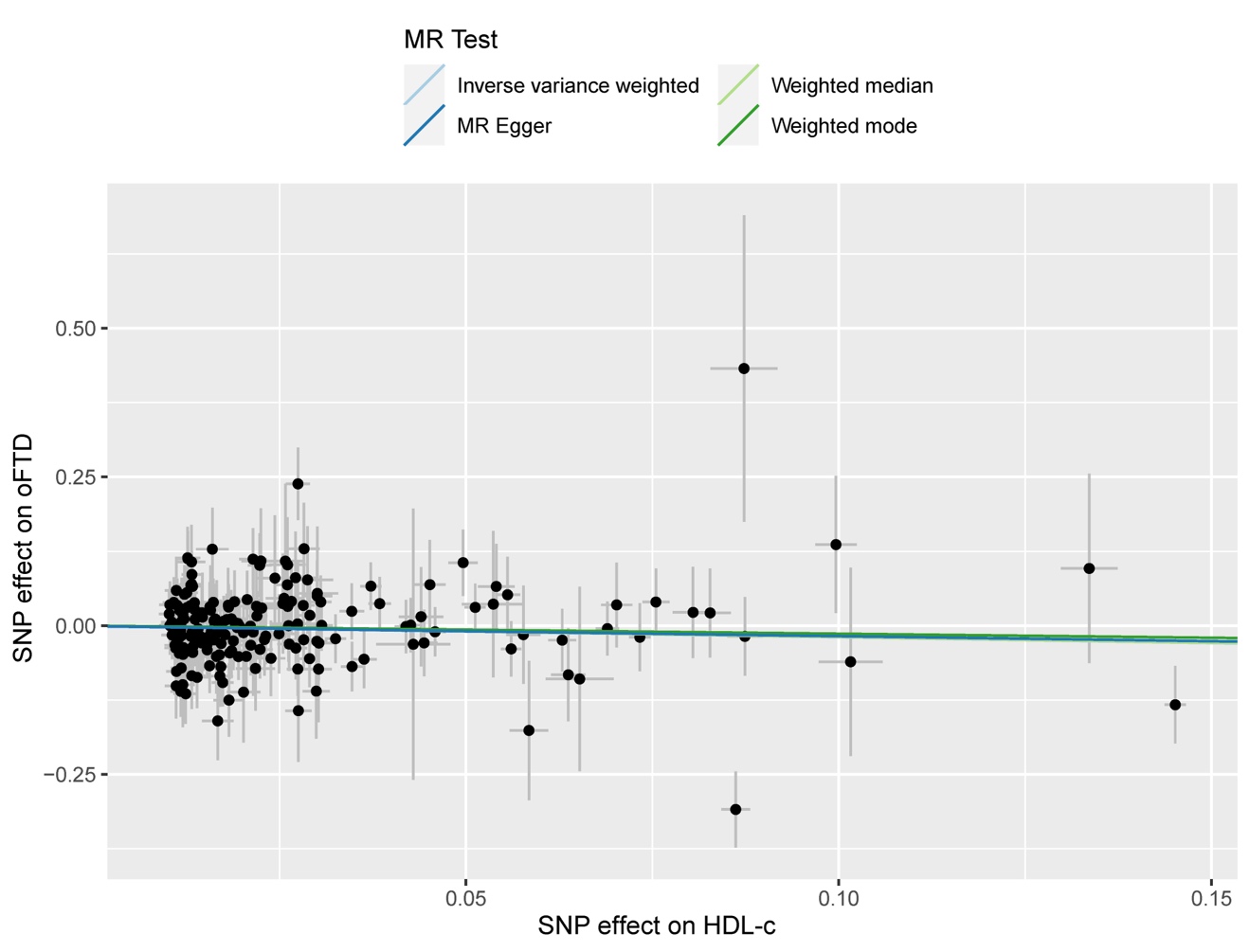


**Figure S27**. Scatter plot of individual SNP-FTD and SNP-HDL-c associations with an overlay of the causal estimate from each MR test in two-sample MR analysis.


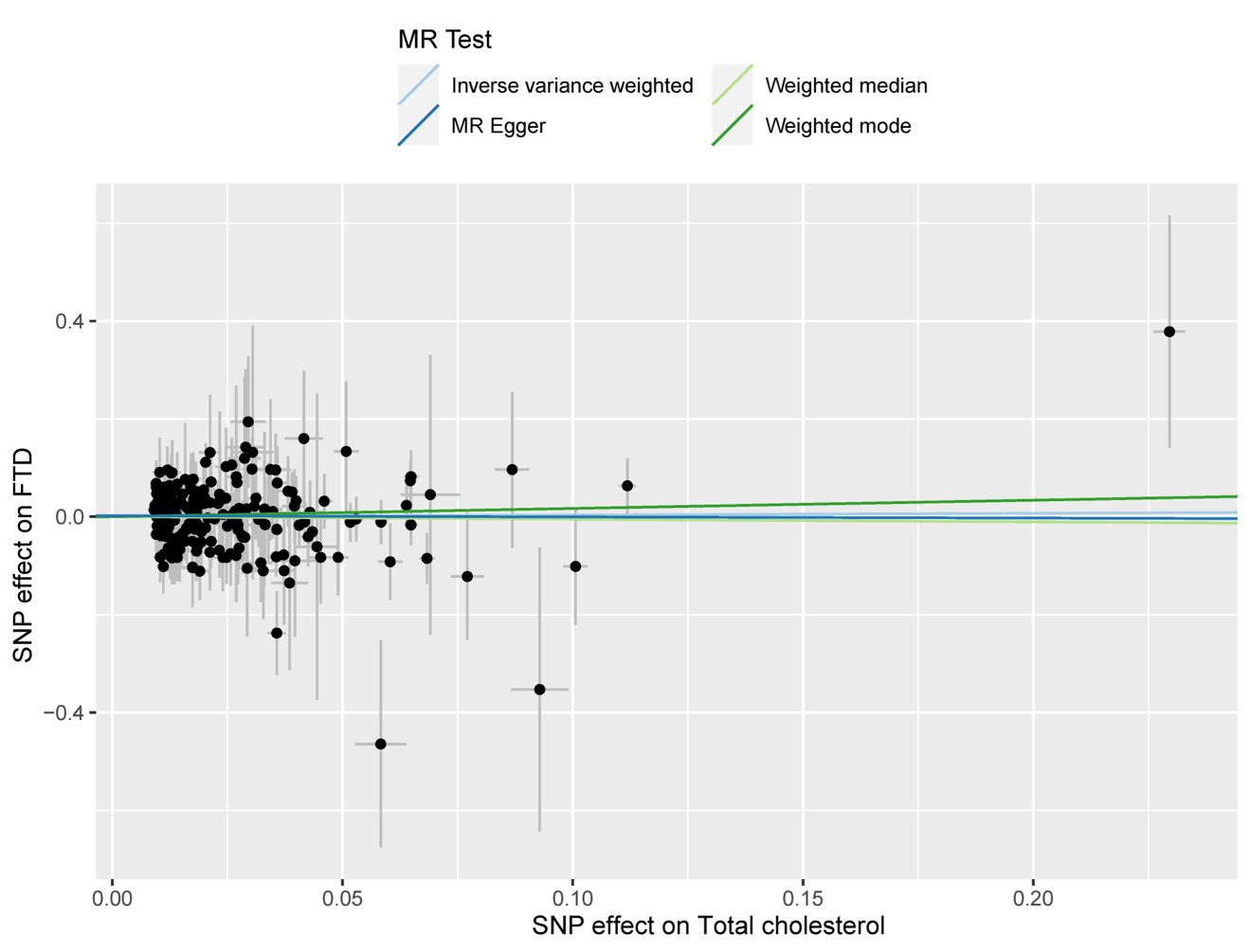


**Figure S28**. Scatter plot of individual SNP-FTD and SNP-total cholesterol associations with an overlay of the causal estimate from each MR test in two-sample MR analysis.


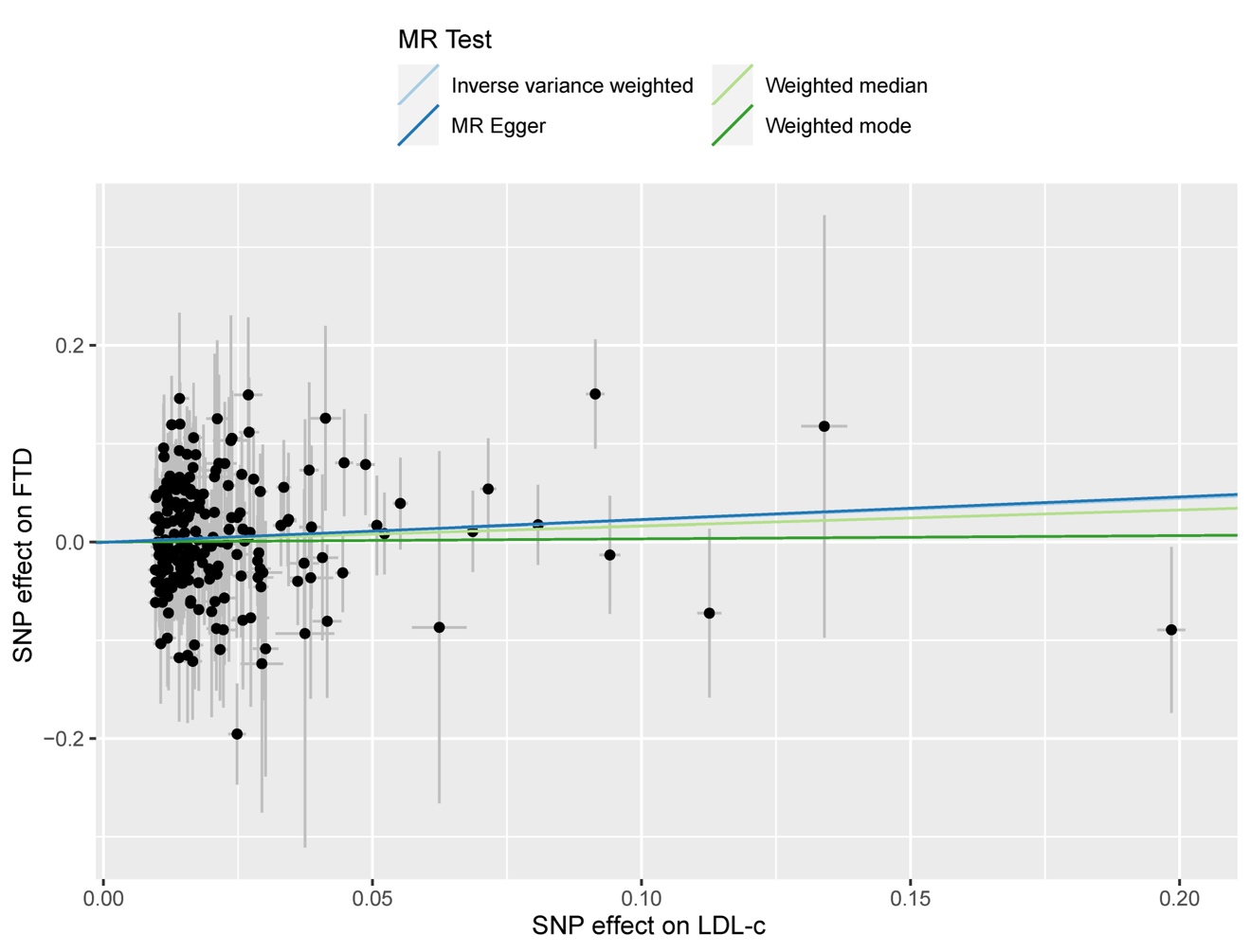


**Figure S29**. Scatter plot of individual SNP-FTD and SNP-triglycerides associations with an overlay of the causal estimate from each MR test in two-sample MR analysis.


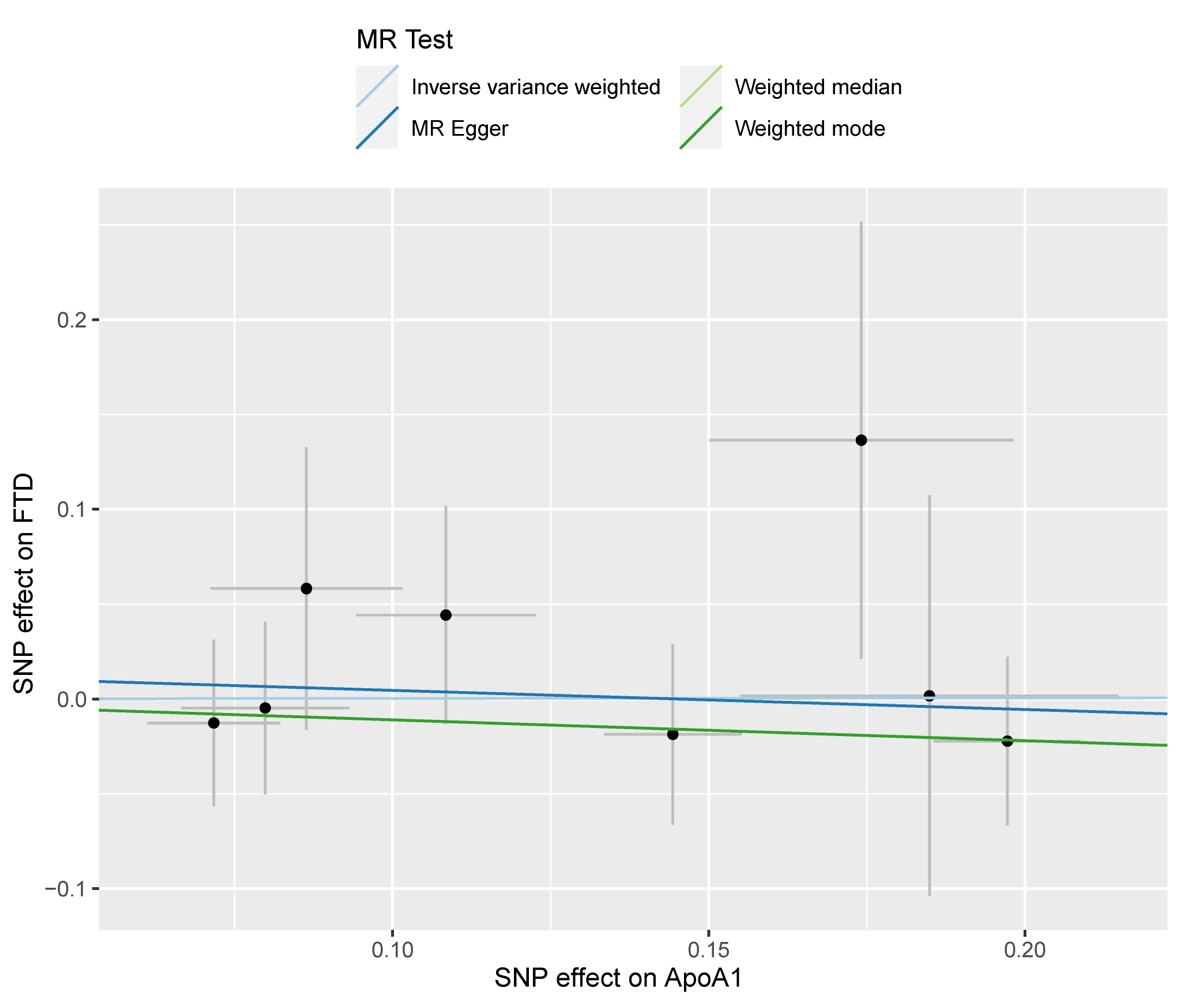


**Figure S30.** Scatter plot of individual SNP-FTD and SNP-ApoA1 associations with an overlay of the causal estimate from each MR test in two-sample MR analysis.


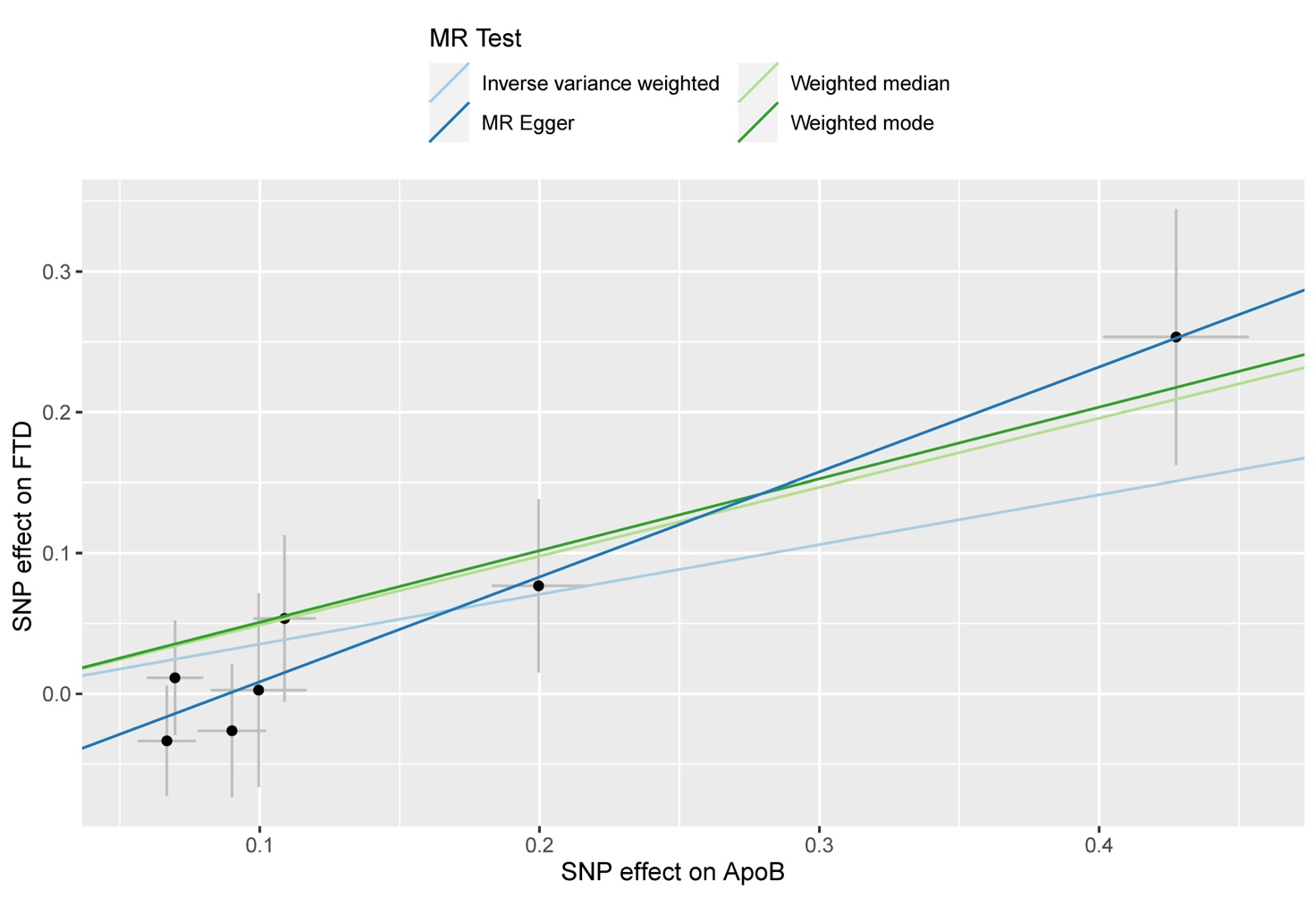


**Figure S31**. Scatter plot of individual SNP-FTD and SNP-ApoB associations with an overlay of the causal estimate from each MR test in two-sample MR analysis.

**Table S5**. Univariate MR of the association of genetically proxied lipid-lowering drug targets with coronary artery disease (positive control analysis)

| **Exposure** | **MR method** | **Outcome** | **nSNP** | **OR** | **95%LCI** | **95%UCI** | **pval** |
| --- | --- | --- | --- | --- | --- | --- | --- |
| HMGCR | Inverse variance weighted | CAD | 28 | 0.658 | 0.564 | 0.768 | **1.06E-07** |
|  | Weighted median | CAD | 28 | 0.684 | 0.556 | 0.843 | **3.56E-04** |
|  | Weighted mode | CAD | 28 | 0.696 | 0.583 | 0.831 | **4.41E-04** |
|  | MR Egger | CAD | 28 | 0.583 | 0.335 | 1.015 | 6.74E-02 |
|  | MR RAPS | CAD | 28 | 0.657 | 0.562 | 0.767 | **1.15E-07** |
| PCSK9 | Inverse variance weighted | CAD | 44 | 0.519 | 0.463 | 0.580 | **2.72E-30** |
|  | Weighted median | CAD | 44 | 0.539 | 0.457 | 0.636 | **2.06E-13** |
|  | Weighted mode | CAD | 44 | 0.534 | 0.453 | 0.629 | **2.34E-09** |
|  | MR Egger | CAD | 44 | 0.520 | 0.431 | 0.628 | **2.70E-08** |
|  | MR RAPS | CAD | 44 | 0.518 | 0.443 | 0.605 | **2.72E-52** |
| NCP1L1 | Inverse variance weighted | CAD | 9 | 0.535 | 0.365 | 0.785 | **1.36E-03** |
|  | Weighted median | CAD | 9 | 0.577 | 0.363 | 0.917 | **1.99E-02** |
|  | Weighted mode | CAD | 9 | 0.589 | 0.366 | 0.950 | 6.16E-02 |
|  | MR Egger | CAD | 9 | 1.008 | 0.308 | 3.296 | 9.90E-01 |
|  | MR RAPS | CAD | 9 | 0.525 | 0.376 | 0.734 | **1.60E-04** |
| APOB | Inverse variance weighted | CAD | 35 | 0.698 | 0.624 | 0.781 | **3.19E-10** |
|  | Weighted median | CAD | 35 | 0.704 | 0.618 | 0.803 | **1.67E-07** |
|  | Weighted mode | CAD | 35 | 0.721 | 0.620 | 0.840 | **1.74E-04** |
|  | MR Egger | CAD | 35 | 0.590 | 0.451 | 0.772 | **5.24E-04** |
|  | MR RAPS | CAD | 35 | 0.697 | 0.642 | 0.758 | **7.49E-11** |


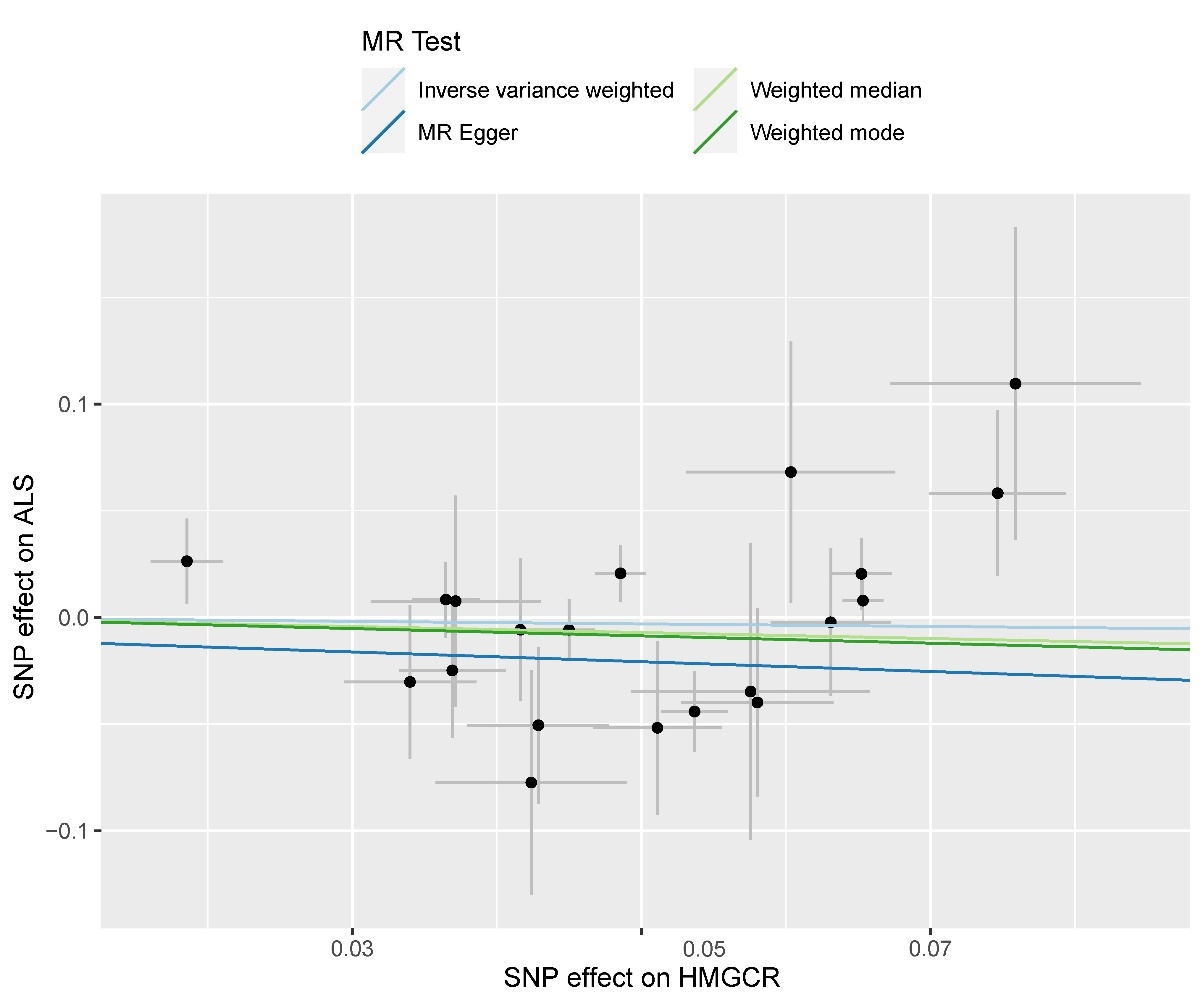


**Figure S32.** Scatter plot of individual SNP-ALS and SNP-genetically proxied HMGCR inhibition associations with an overlay of the causal estimate from each MR test in two-sample MR analysis.


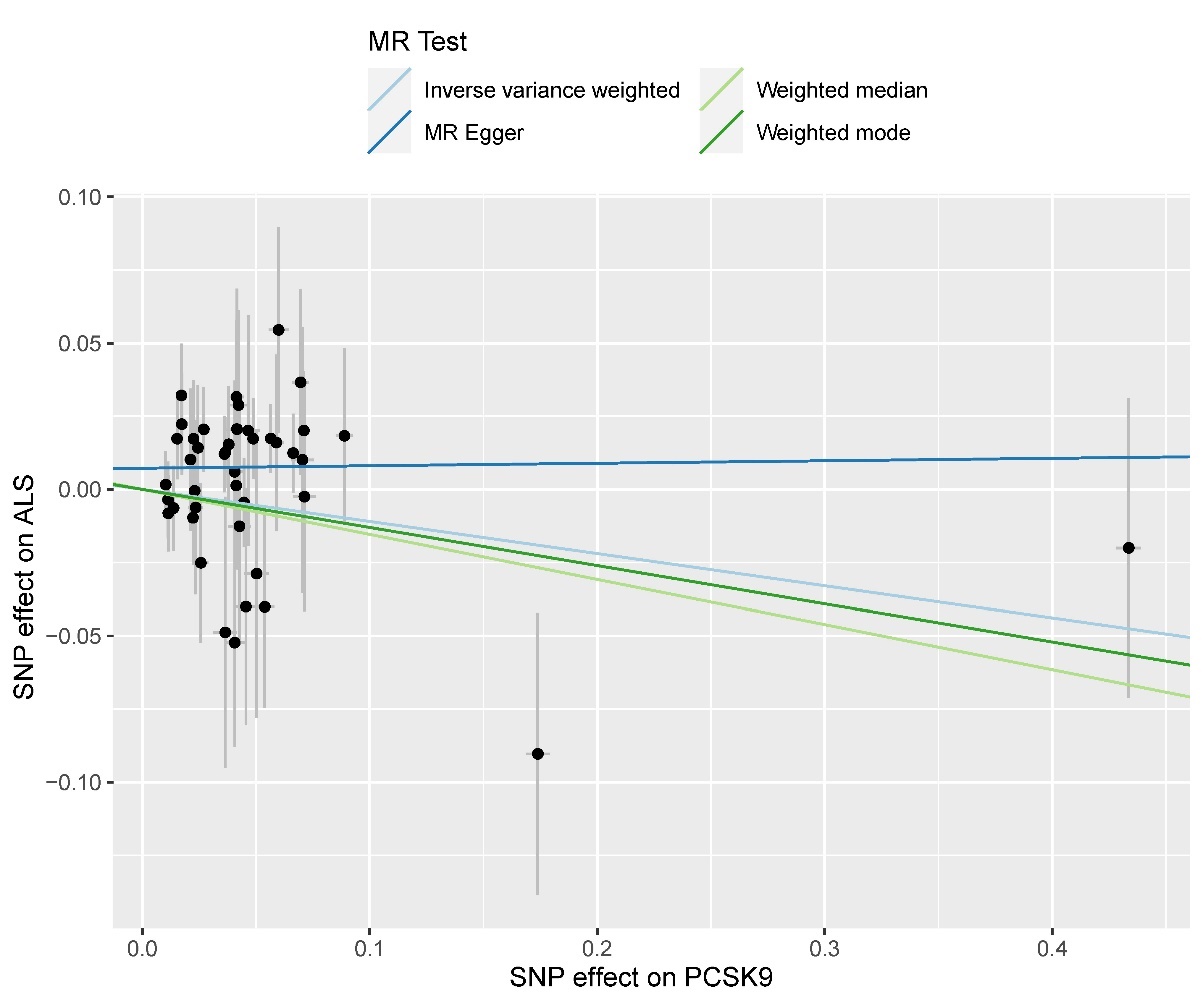


**Figure S33**. Scatter plot of individual SNP-ALS and SNP-genetically proxied PCSK9 inhibition associations with an overlay of the causal estimate from each MR test in two-sample MR analysis.


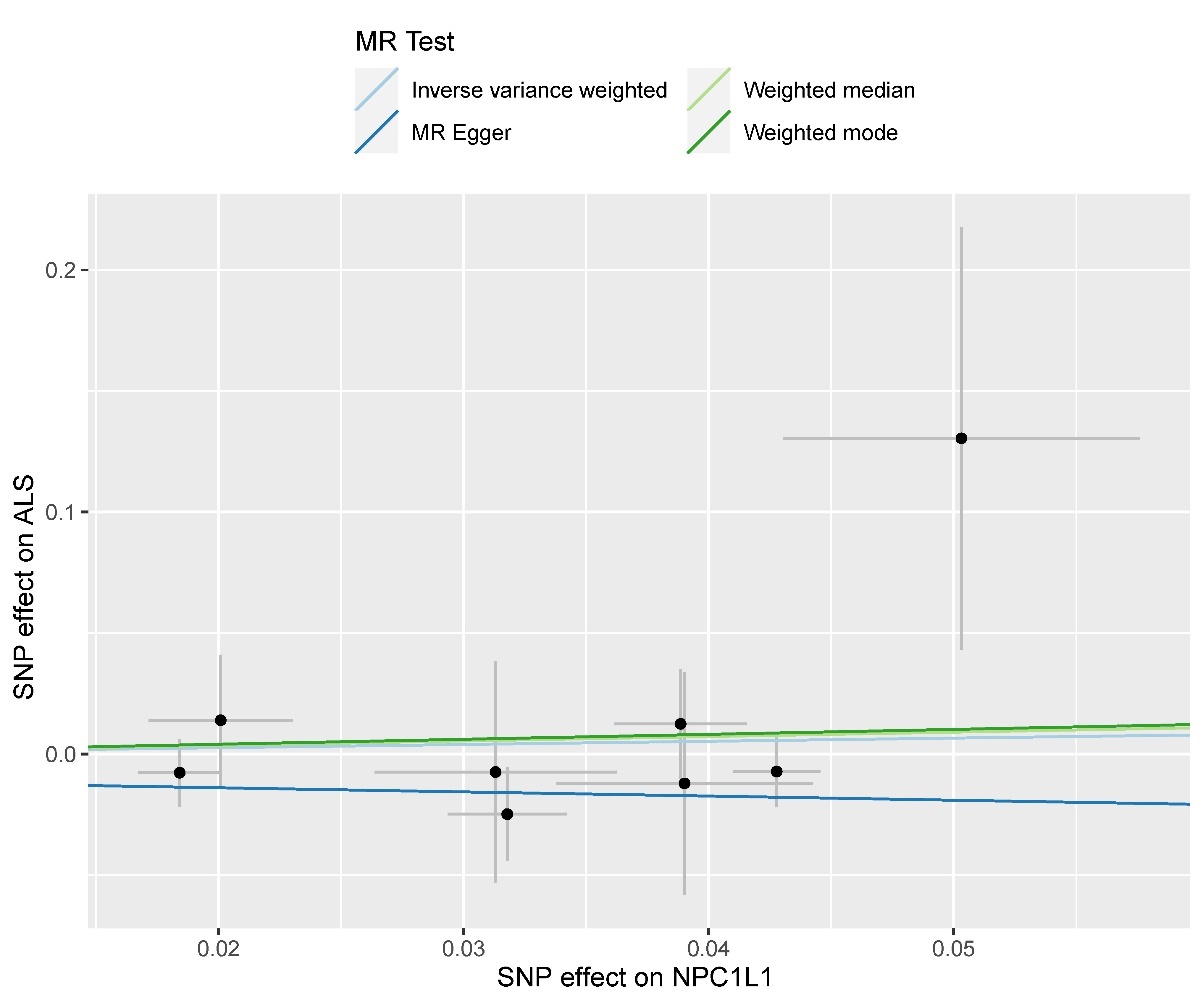


**Figure S34**. Scatter plot of individual SNP-ALS and SNP-genetically proxied NPC1L1 inhibition associations with an overlay of the causal estimate from each MR test in two-sample MR analysis.


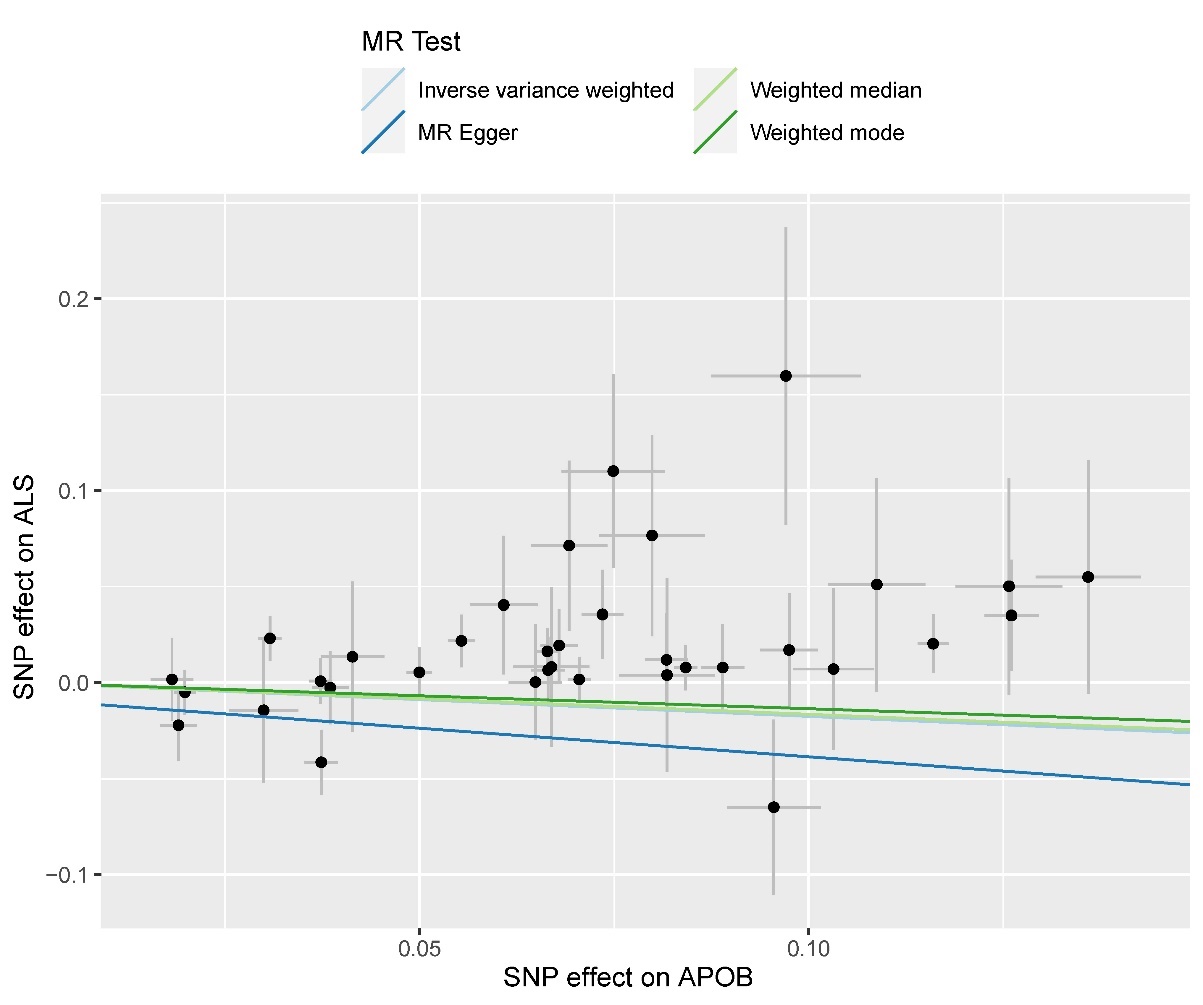


**Figure S35.** Scatter plot of individual SNP-ALS and SNP-genetically proxied APOB inhibition associations with an overlay of the causal estimate from each MR test in two-sample MR analysis.


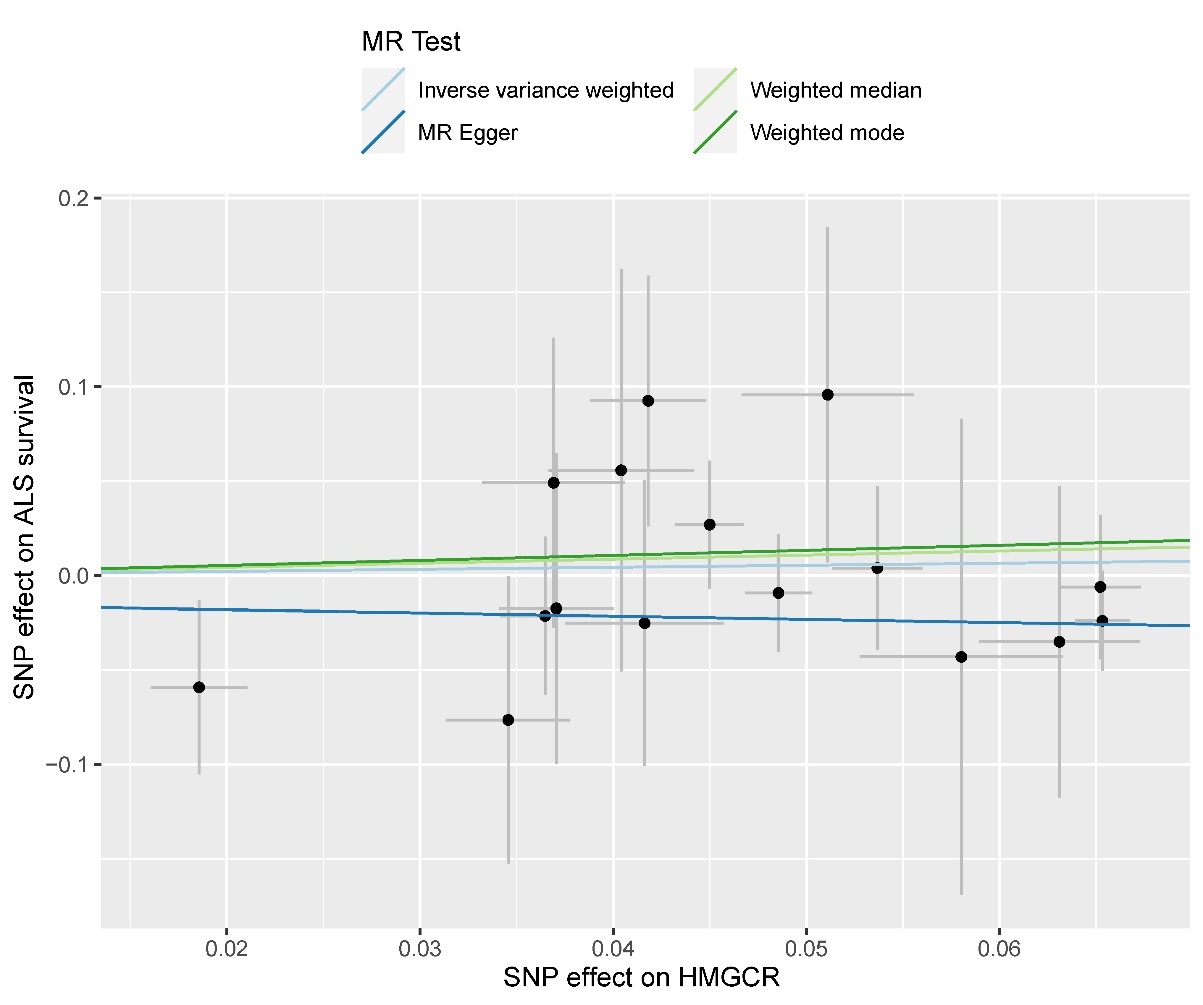


**Figure S36**. Scatter plot of individual SNP-ALS survival and SNP-genetically proxied HMGCR inhibition associations with an overlay of the causal estimate from each MR test in two-sample MR analysis.


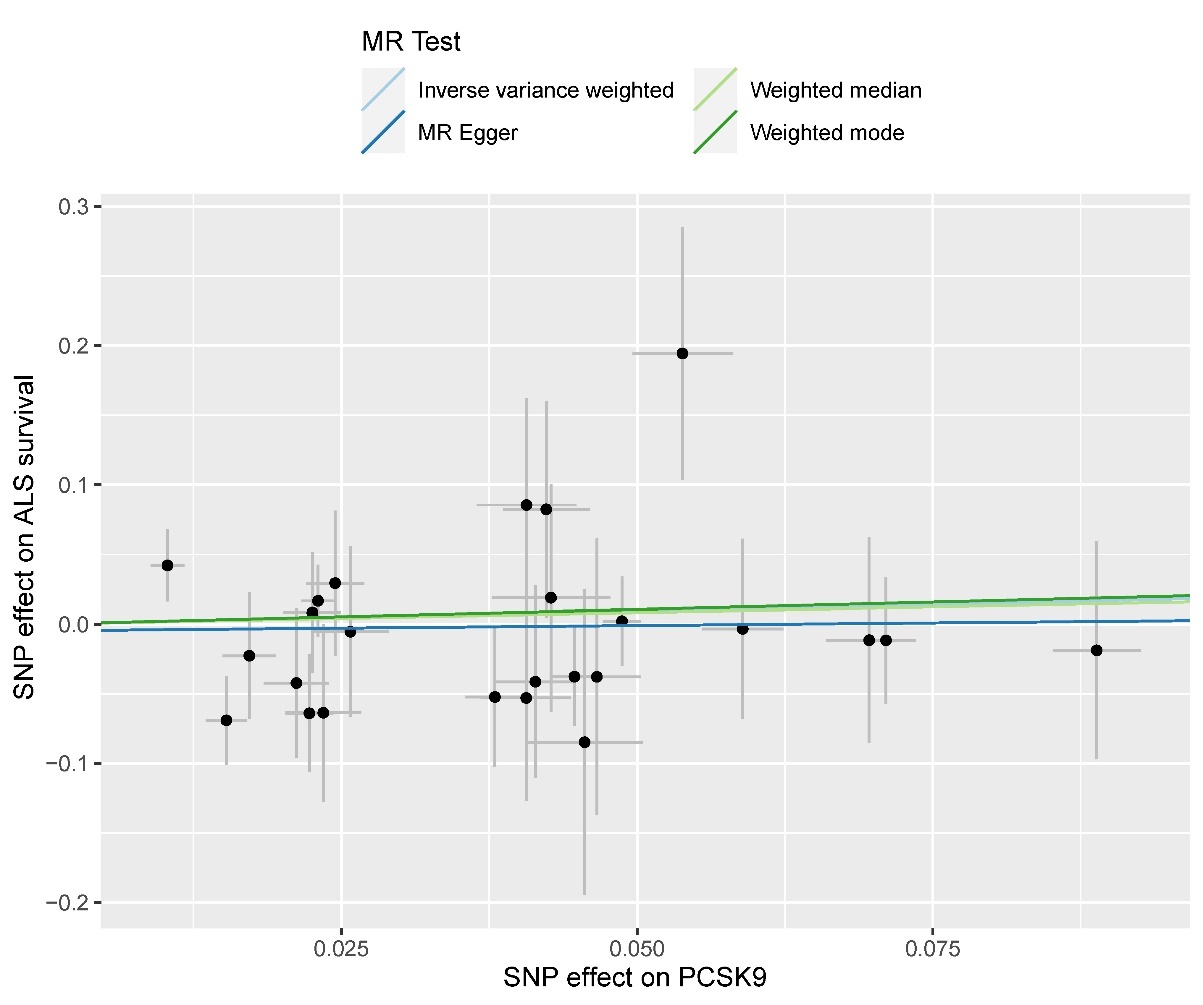


**Figure S37.** Scatter plot of individual SNP-ALS survival and SNP-genetically proxied PCSK9 inhibition associations with an overlay of the causal estimate from each MR test in two-sample MR analysis.


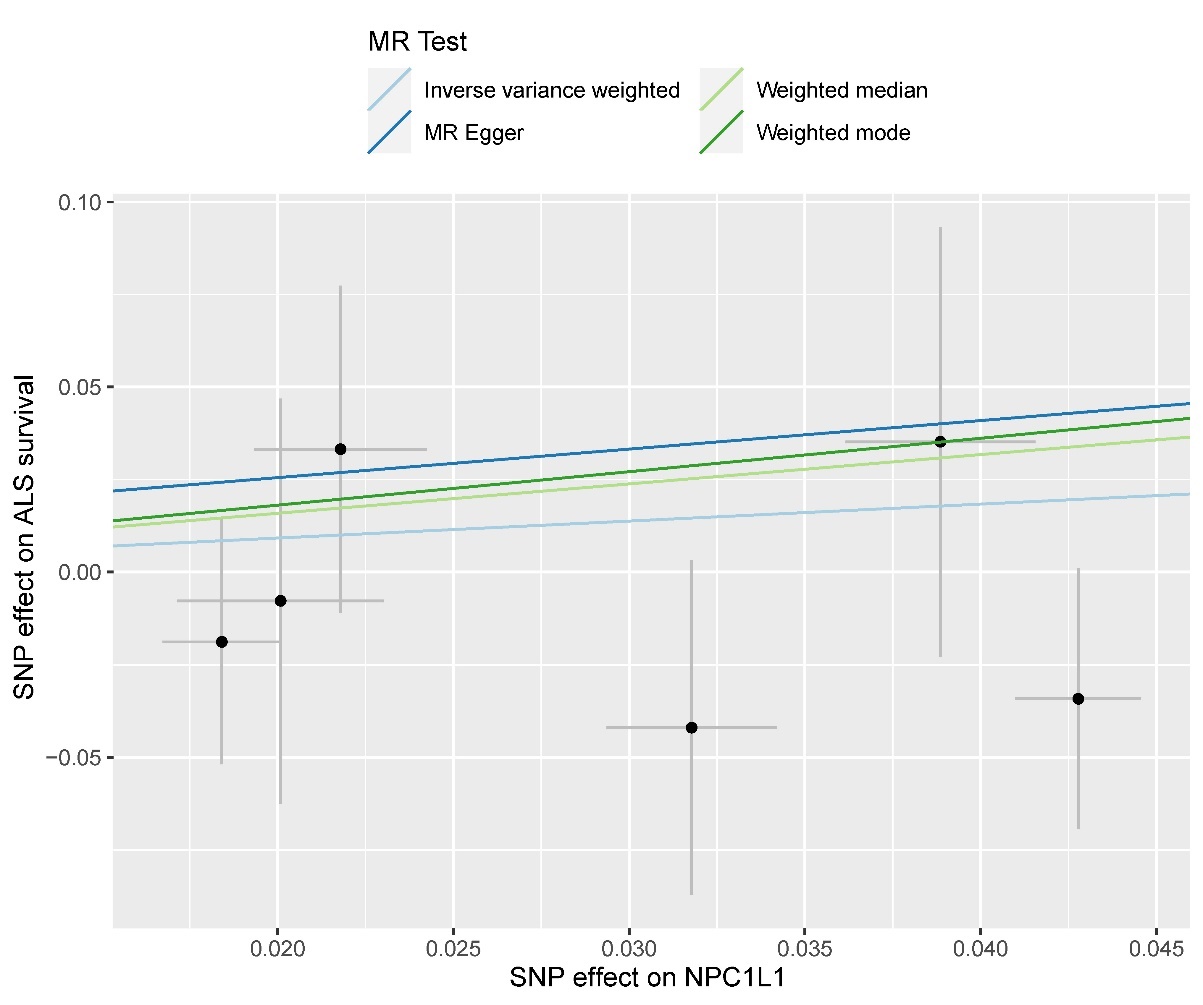


**Figure S38**. Scatter plot of individual SNP-ALS and SNP-genetically proxied NPC1L1 inhibition associations with an overlay of the causal estimate from each MR test in two-sample MR analysis.


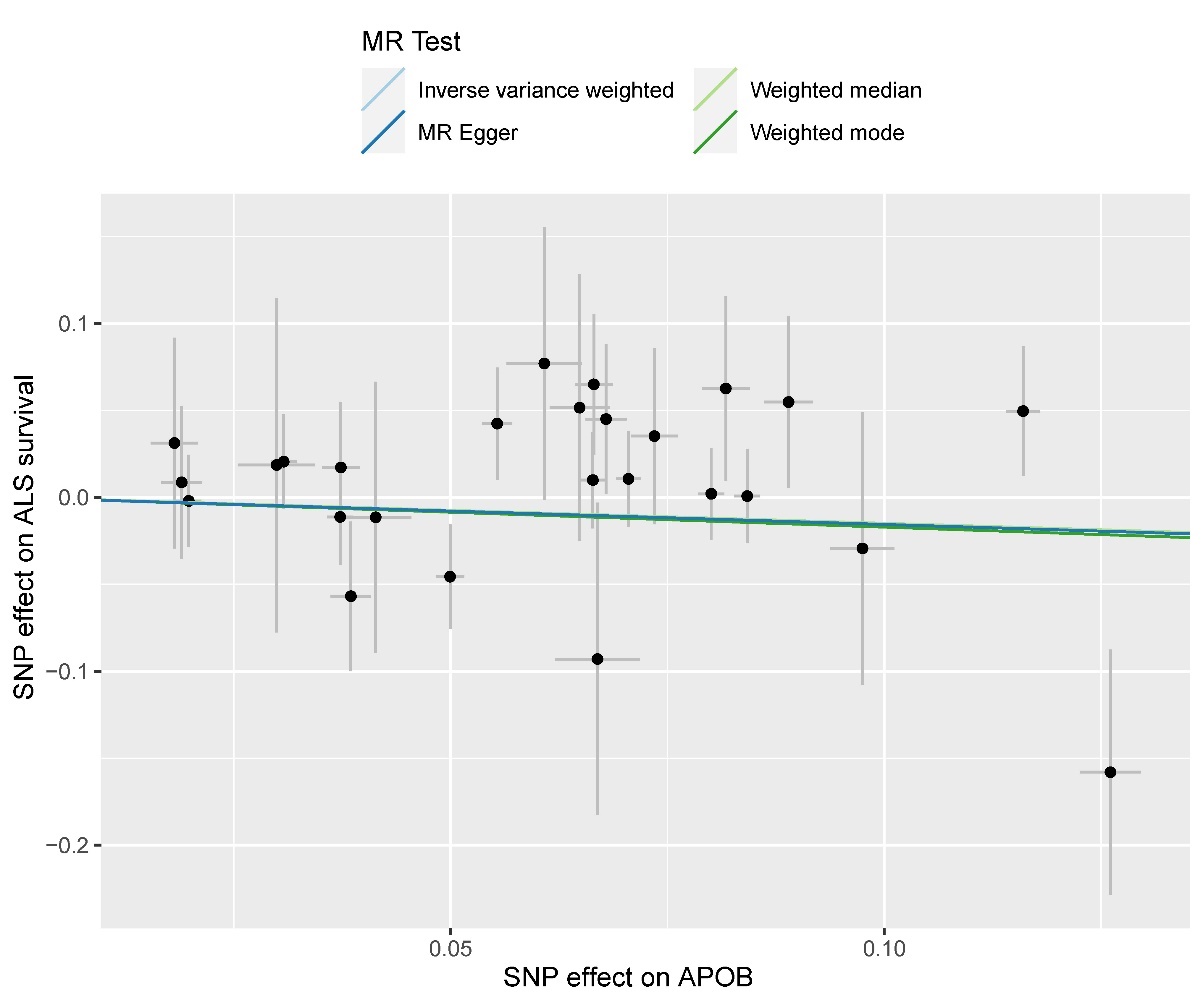


**Figure S39**. Scatter plot of individual SNP-ALS survival and SNP-genetically proxied APOB inhibition associations with an overlay of the causal estimate from each MR test in two-sample MR analysis.


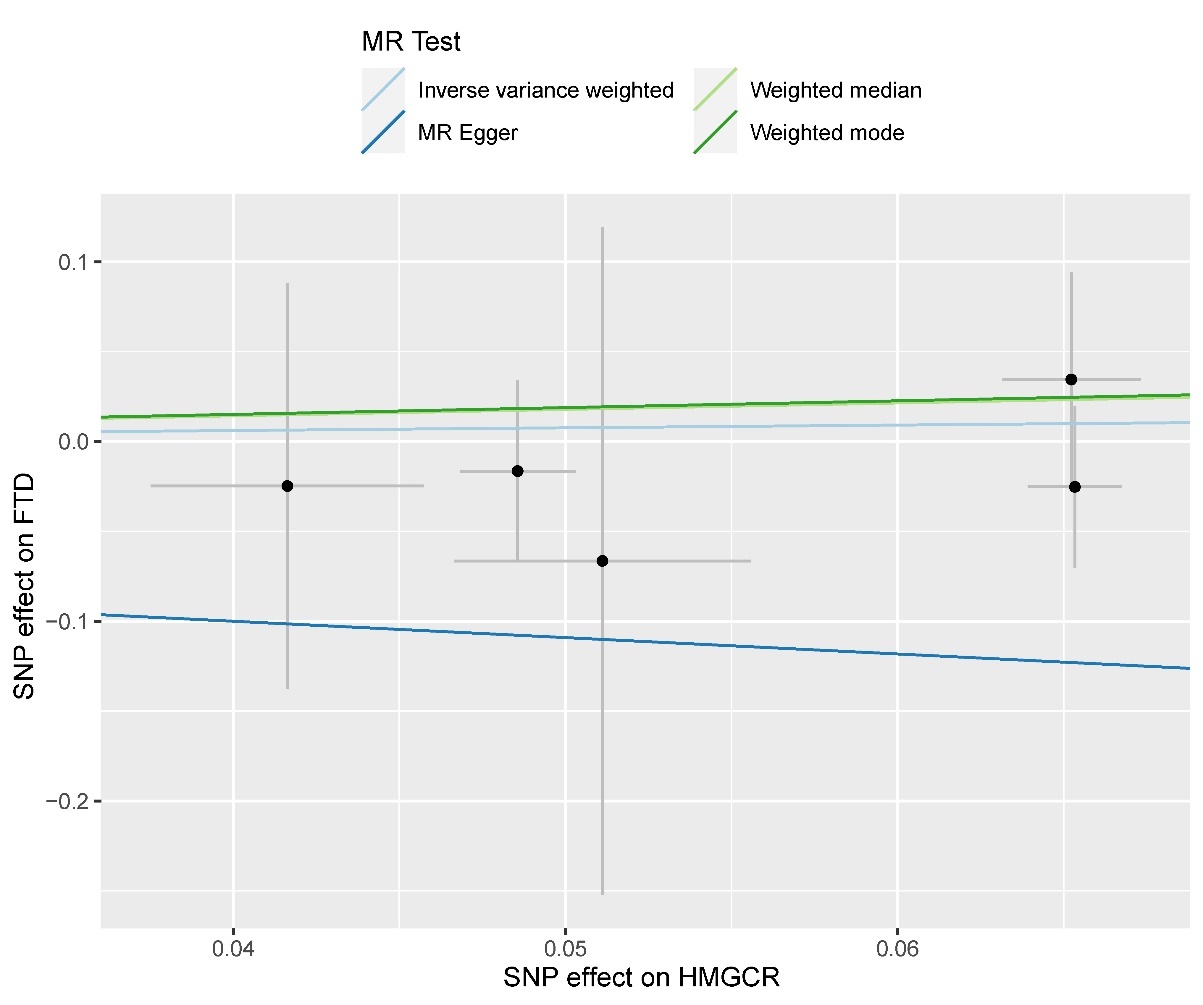


**Figure S40**. Scatter plot of individual SNP-FTD and SNP-genetically proxied HMGCR inhibition associations with an overlay of the causal estimate from each MR test in two-sample MR analysis.


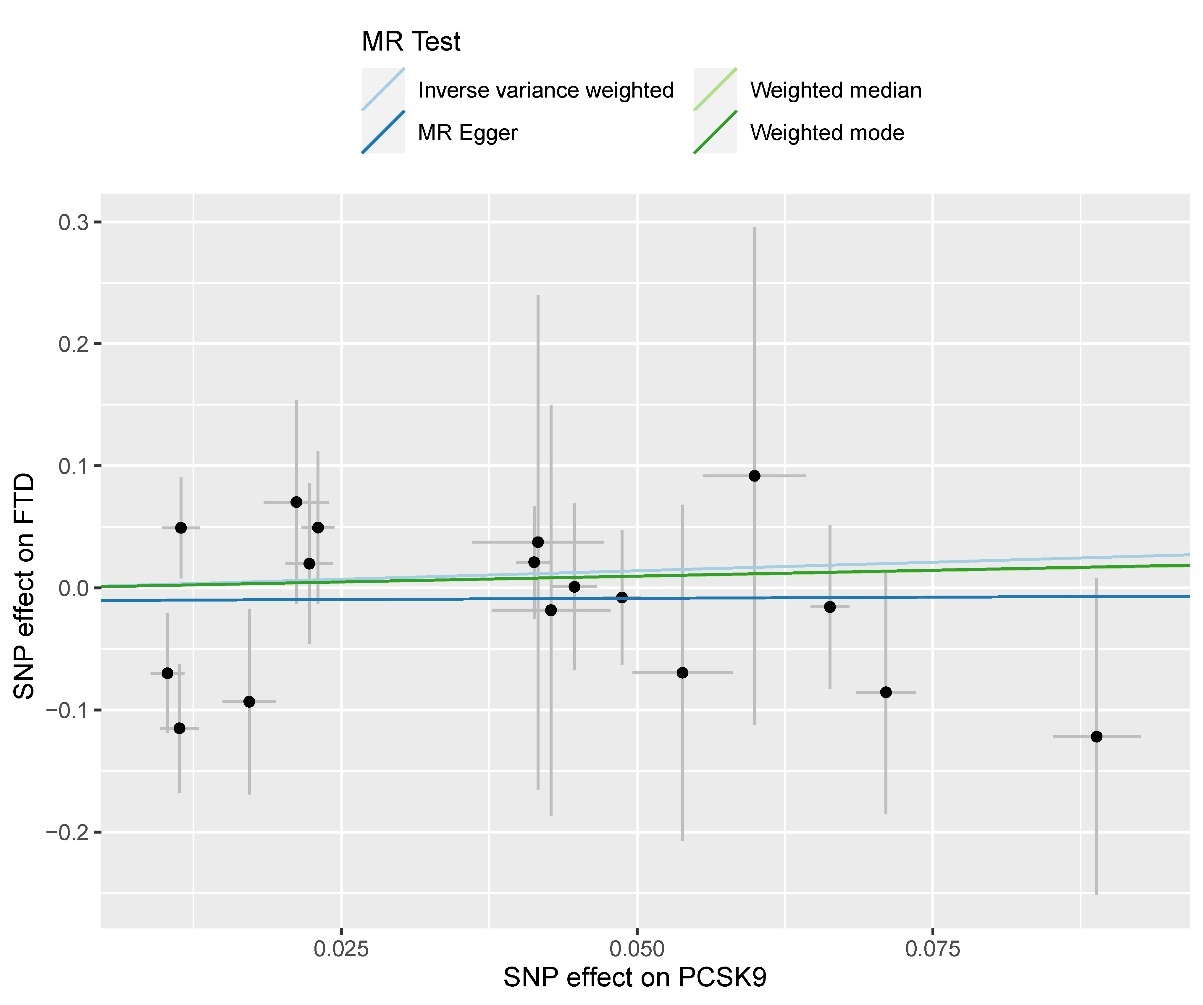


**Figure S41.** Scatter plot of individual SNP-FTD and SNP-genetically proxied PCSK9 inhibition associations with an overlay of the causal estimate from each MR test in two-sample MR analysis.


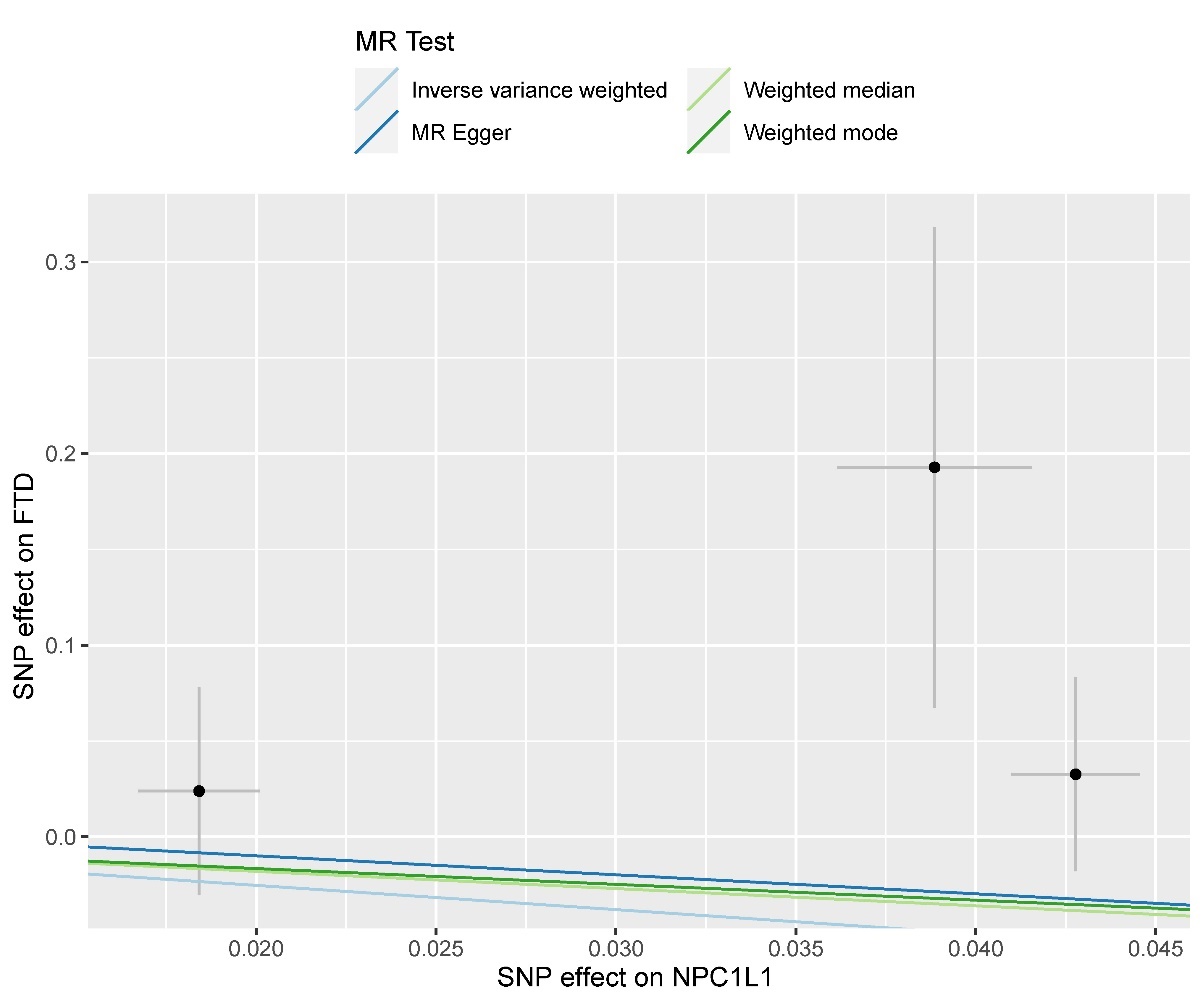


**Figure S42**. Scatter plot of individual SNP-FTD and SNP-genetically proxied NPC1L1 inhibition associations with an overlay of the causal estimate from each MR test in two-sample MR analysis.


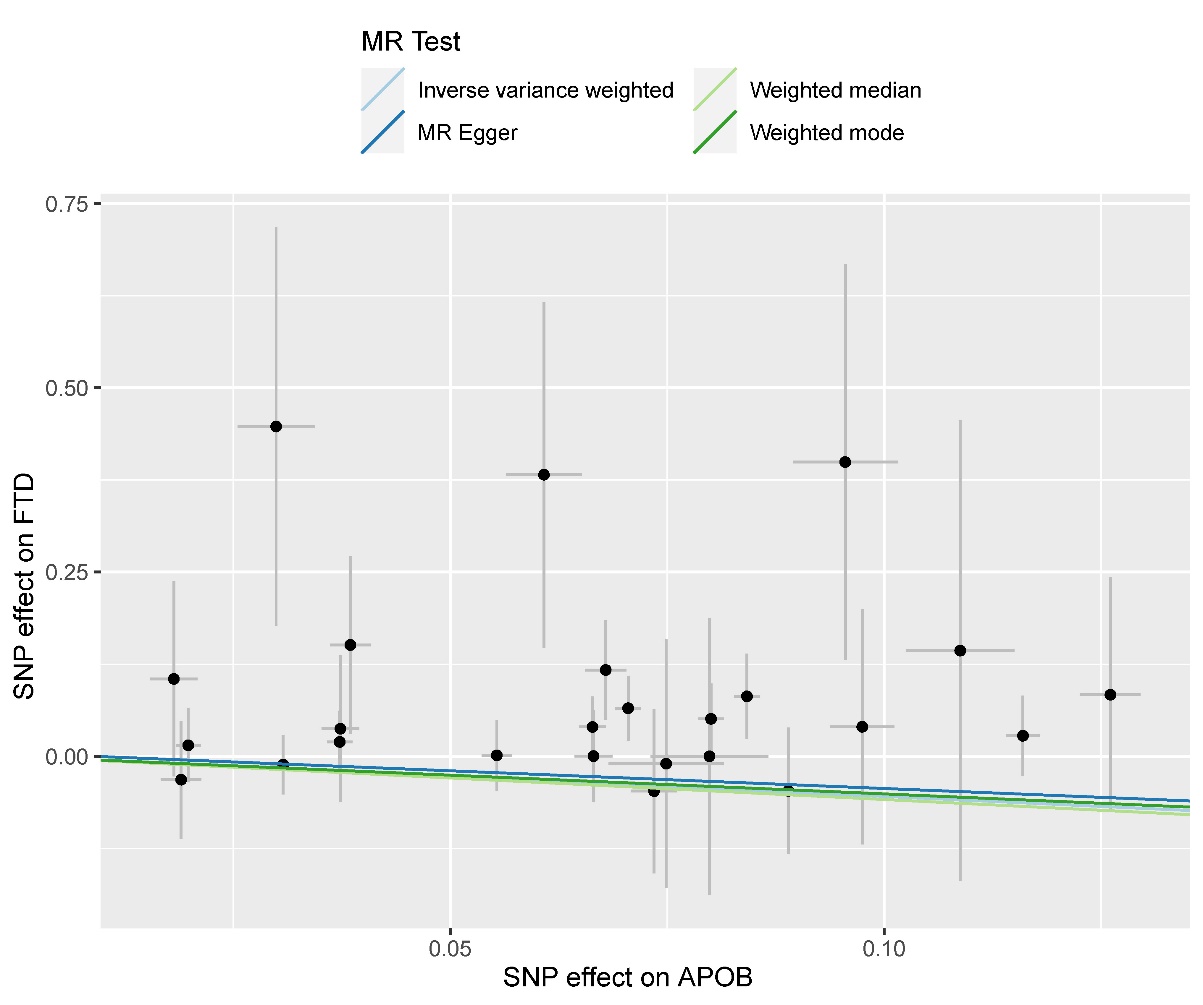


**Figure S43**. Scatter plot of individual SNP-FTD and SNP-genetically proxied APOB inhibition associations with an overlay of the causal estimate from each MR test in two-sample MR analysis.


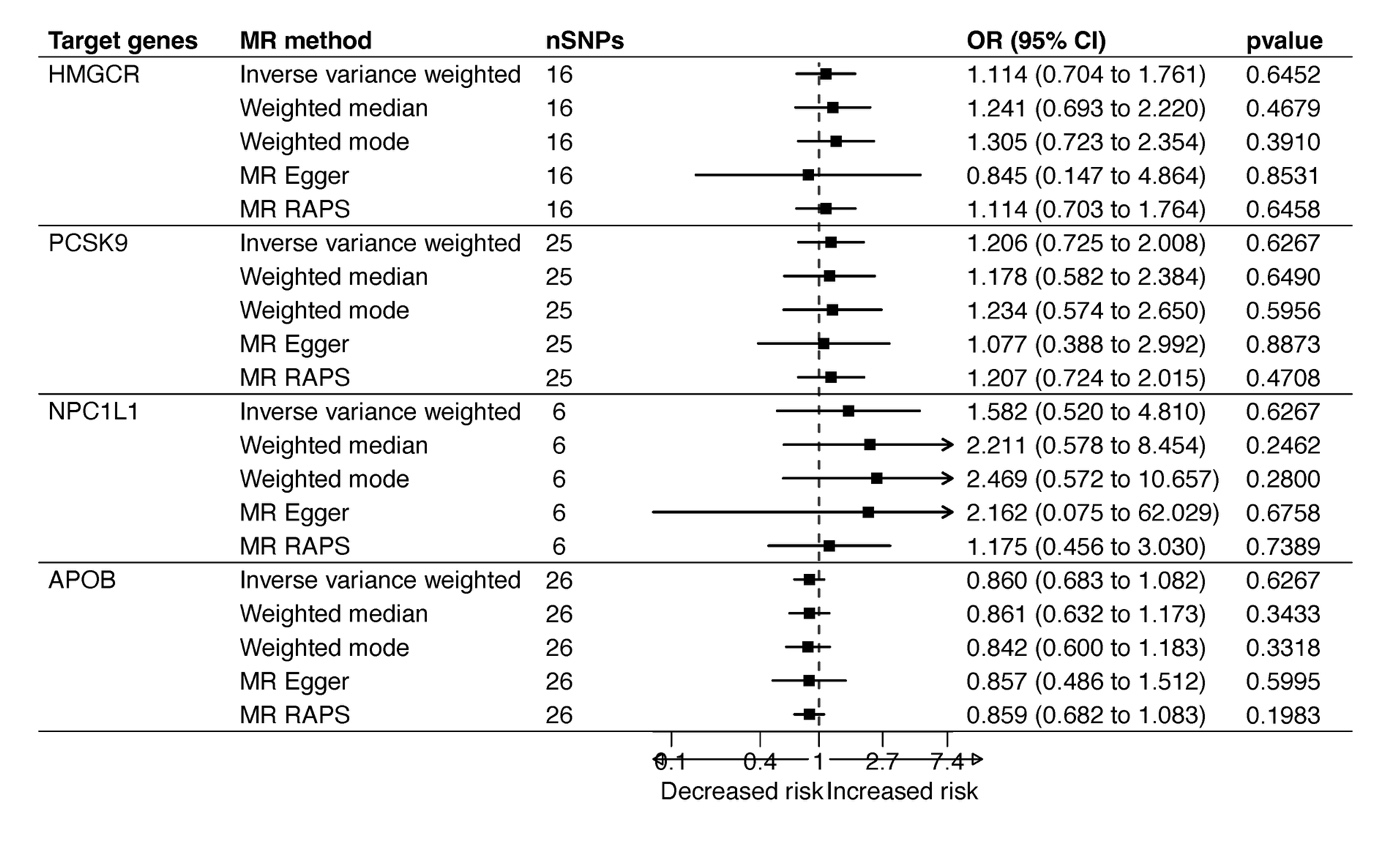


**Figure S44**. Univariate MR of the association of genetically proxied lipid-lowering drug targets with ALS survival. Forest plot of the association between a 1-SD change in the LDL-c levels of four lipid-lowering drug targets with ALS and FTD risk. An effect size of <1.00 suggests a decreased risk of disease associated with lipid-lowering drug treatment. Abbreviations: LDL-c, low-density lipoprotein cholesterol; OR, odds ratio; HR, hazard ratio; SNP, single-nucleotide polymorphisms; HMGCR, HMG-CoA reductase; NPC1L1, Niemann-Pick C1-like protein 1; PCSK9, proprotein convertase subtilisin/kexin type 9; APOB, Apolipoprotein B-100.
